# First modern human settlement recorded in the Iberian hinterland occurred during Heinrich Stadial 2 within harsh environmental conditions

**DOI:** 10.1038/s41598-021-94408-w

**Published:** 2021-07-26

**Authors:** M. Alcaraz-Castaño, J. J. Alcolea-González, M. de Andrés-Herrero, S. Castillo-Jiménez, F. Cuartero, G. Cuenca-Bescós, M. Kehl, J. A. López-Sáez, L. Luque, S. Pérez-Díaz, R. Piqué, M. Ruiz-Alonso, G.-C. Weniger, J. Yravedra

**Affiliations:** 1 Prehistory Area, University of Alcalá, Alcalá de Henares, Spain; 2 Atapuerca Foundation, Burgos, Spain; 3 Aragosaurus-IUCA, Department of Geosciences, University of Zaragoza, Zaragoza, Spain; 4 Institute of Geography, University of Cologne, Cologne, Germany; 5 Environmental Archeology Research Group, Institute of History, CCHS CSIC, Madrid, Spain; 6 Department of Geography, Urban and Regional Planning, University of Cantabria, Santander, Spain; 7 Department of Prehistory, Autonomous University of Barcelona, Barcelona, Spain; 8 Neanderthal Museum, Mettmann, Germany; 9 Department of Prehistory, Complutense University of Madrid, Madrid, Spain

**Keywords:** Stratigraphy, Archaeology, Palaeontology, Palaeoecology, Archaeology, Sedimentology

## Abstract

As the south-westernmost region of Europe, the Iberian Peninsula stands as a key area for understanding the process of modern human dispersal into Eurasia. However, the precise timing, ecological setting and cultural context of this process remains controversial concerning its spatiotemporal distribution within the different regions of the peninsula. While traditional models assumed that the whole Iberian hinterland was avoided by modern humans due to ecological factors until the retreat of the Last Glacial Maximum, recent research has demonstrated that hunter-gatherers entered the Iberian interior at least during Solutrean times. We provide a multi-proxy geoarchaeological, chronometric and paleoecological study on human–environment interactions based on the key site of Peña Capón (Guadalajara, Spain). Results show (1) that this site hosts the oldest modern human presence recorded to date in central Iberia, associated to pre-Solutrean cultural traditions around 26,000 years ago, and (2) that this presence occurred during Heinrich Stadial 2 within harsh environmental conditions. These findings demonstrate that this area of the Iberian hinterland was recurrently occupied regardless of climate and environmental variability, thus challenging the widely accepted hypothesis that ecological risk hampered the human settlement of the Iberian interior highlands since the first arrival of modern humans to Southwest Europe.

## Introduction

### The first modern human settlement of southwest Europe and the role of the Iberian hinterland

**Figure 1:**
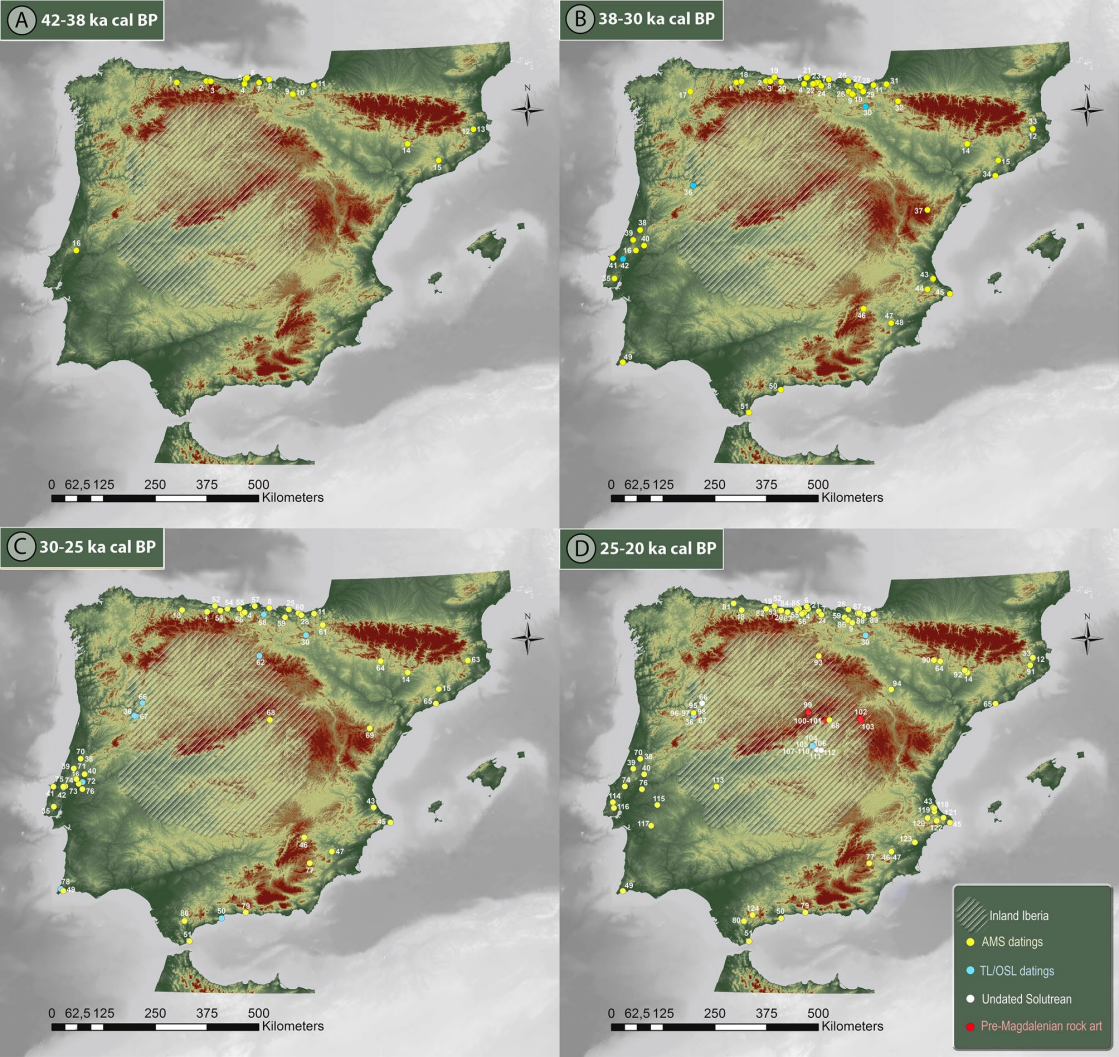
Process of peopling of the Iberian Peninsula by modern humans during the Upper Paleolithic. A: 42 – 38 ka cal BP, B: 38 – 30 ka cal BP, C: 30–25 ka cal BP, D: 25–20 ka cal BP (see Supplementary Text S3 for discussion and Supplementary Datasets 1–4 for full data). Maps generated with ArcGIS (ArcMap 10.3.1.) (https://www.arcgis.com/index.html) using ASTER Global Digital Elevation Model V0032019, distributed by NASA EOSDIS Land Processes DAAC, (10.5067/ASTER/ASTGTM.003).

The first appearance of anatomically modern humans in a given region of the world is always a contentious topic. In Western Europe, the Iberian Peninsula stands as the last region of the process of modern human dispersal into Eurasia, and hence is considered of key importance for understanding its cultural and natural constraints. However, the precise timing, ecological setting and cultural context of this process remains especially controversial when considering its spatiotemporal distribution within the different regions of the peninsula. Bearing aside the controversy on the makers of the Chatelperronian and other so-called transitional technocomplexes^1–3^, if we accept the Proto-Aurignacian as the first proxy for modern humans in Western Europe, these people were present in the Cantabrian and northern Mediterranean regions of Iberia at ∼42 ka cal BP^4,5^ (Fig. 1A), or even 43 ka cal BP^6^. This is a roughly similar time as recorded in other regions of Western and Central Europe^7,8^, although significantly younger than in Eastern Europe according to recent data^9,10^. However, in light of prevailing evidence, it was much later when Aurignacian cultures spread to the southern parts of Iberia, reaching the southwesternmost regions of Europe at around 36.5 ka cal BP^11^, probably matching the time of the Neandertals’ final demise^12–14^ (Fig. 1B). Significantly earlier occurrences of Proto-Aurignacian have been recently claimed for the sites of Bajondillo (∼43.0–40.8 ka cal BP)^15^ and Lapa do Picareiro (∼41.1–38.1 ka cal BP)^16^, in southern Spain and central Portugal respectively. However, published archaeological and chronostratigraphic data in the case of Bajondillo are disputable and have been strongly contested^14,17–19^.

**Figure 2:**
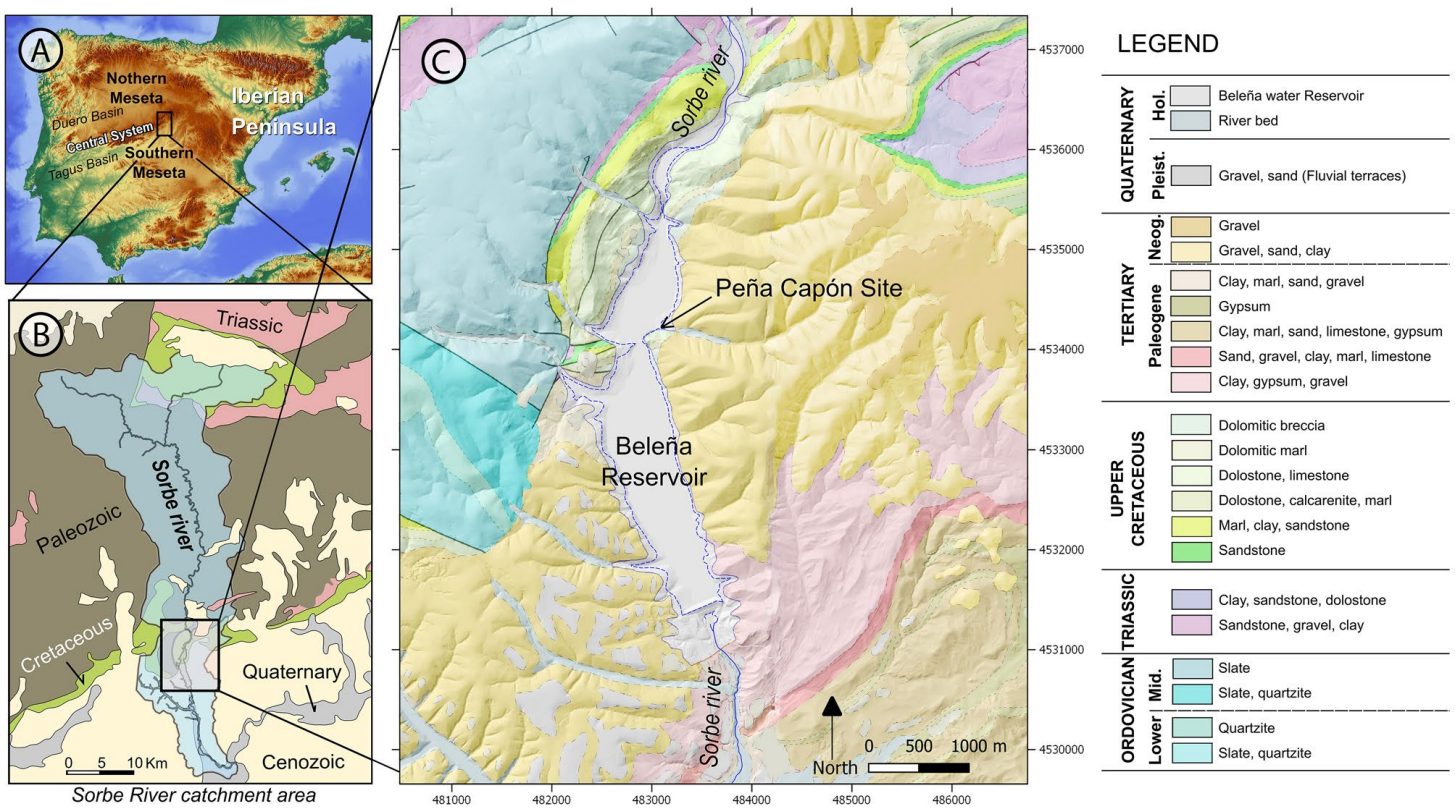
Geological maps showing the location of the Peña Capón rock shelter in the Iberian Peninsula and the Tagus basin (Guadalajara, Spain) (**A**), the Sorbe River basin (**B**) and at the shore of the Beleña water reservoir (**C**). Maps generated using QGIS Open Source Geographic Information System v. 3.4 (Madeira) (https://www.qgis.org/en/site/about/index.html) combined with Digital Terrain Models and slope maps from the Spanish National Centre for Geographic Information (CNIG) (https://www.ign.es/web/ign/portal/qsm-cnig) and geological maps from the Spanish Geological Survey (IGME) (https://www.igme.es/zaragoza/ingles/inicio.htm).

The vast hinterland territories of the Iberian Peninsula, an upland plateau divided into the Northern and Southern *Mesetas* by the Central System mountain range (Figs. 1 and 2), have been traditionally considered irrelevant in this process, as they have been regarded as a virtually “no-man's land” until the Late Upper Paleolithic^20^. At present, there is some sparse data between 33 and 28 ka cal BP in the western and northern borders of the Northern *Meseta*^14,21–24^ (Fig. 1C). However, to date, effective presence of modern human occupations in the central regions of Iberia is not found until ∼25.5 ka cal BP, as shown by preliminary evidence gathered at one single site: the Peña Capón rock shelter^25,26^ (Fig. 1C).

Reasons behind this odd population pattern have revolved around a potentially late-persisting Neandertal presence in the center and south of Iberia^12^ and, more prominently, the potentially harsh climatic and environmental conditions of the interior and upland regions of Iberia as opposed to the more favored environments of the peninsular coastal regions, long considered as refugia for flora, animals and humans [see^20,27^]. Recent reviews have roughly supported this latter picture, as they have limited the pre-Magdalenian human presence in most of inland Iberia to short-term or sporadic incursions during temperate intervals starting only in Solutrean times (i.e. between 25 and 20 ka cal BP)^11,27,28^. Furthermore, habitat suitability models based mainly on paleoclimate simulations^29–33^ have provided further support to the traditional model. These works describe the Iberian interior as an ecologically risky area for human settlement, especially during the Last Glacial Maximum (LGM) *sensu stricto* (i.e. 23–19 ka BP)^34^, due to its relatively high elevation, degree of climate variability and resource unpredictability (but see^35–37^ for significant differences concerning habitat suitability of the Iberian interior, due to the high number of variables and methods involved in modeling building). Other studies have recently pointed to arid and cold environmental conditions in central Iberia during ∼40–30 ka cal BP. They are based both on paleoecological^38,39^ and sedimentological^40,41^ data and provide further support to the idea that climate and environmental conditions somehow hampered the human occupation of these regions during the beginning of the Upper Paleolithic.

However, the idea of inland Iberia as either a desolate landscape or a mere crossing-area where human groups based elsewhere entered only sporadically during most of the Upper Paleolithic has been under attack in the last years^14,20,42^. Among the growing number of evidence suggesting a more relevant settlement of the Iberian interior not only during the Solutrean, but also before^20,43^, data recorded at the Peña Capón rock shelter has revealed crucial^25,26^ (Supplementary Text S1). Here we report results from new fieldwork and laboratory analyses, including geomorphology, sedimentology, micromorphology, radiocarbon dating, palynology, anthracology, zooarcheology, microvertebrate paleontology and analysis of lithic technology, with the main aim of providing new evidence on human–environment–climate interactions during the first settlement of the Iberian central regions by modern humans recorded to date. Under a theoretical framework that conceives cultural change, population dynamics and adaptive traits of hunter-gatherers as multifactorial responses to fluctuating social and natural parameters^44–46^, including climate and environmental change^47–49^, these results show relevant patterns concerning the timing, nature and ecological setting of this process. More specifically, considered in the context of recent research on the relations between population dynamics, settlement patterns and techno-cultural change in the Late Pleistocene of Iberia on one side, and rapid climate and environmental change on the other^27,29–33,50–56^, our results allow us to test the hypothesis that the first modern human settlement of inland Iberia occurred earlier than previously thought, and was not impeded by ecological variability.

### The Peña Capón rock shelter and its regional setting

**Figure 3:**
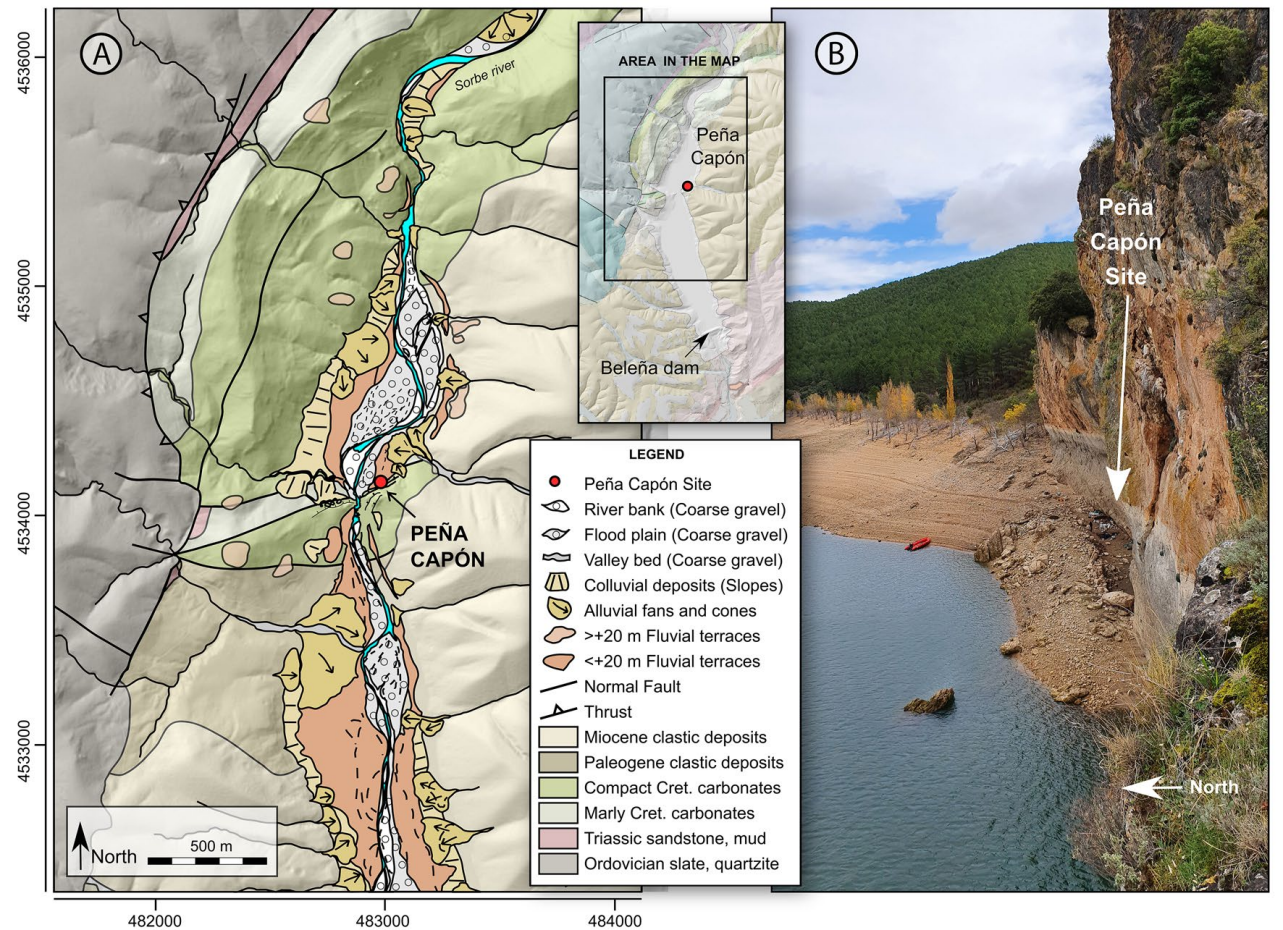
(**A**) Geomorphological map of the study area showing the position of Peña Capon at the foot of a dolomite cretaceous relief, and the distribution of the Quaternary deposits located in the area. (**B**) General view of the site from above. Map generated as explained in Fig. 2.

The Peña Capón rock shelter (Guadalajara province, Spain) is located near the left bank of the Sorbe River, which flows into the Henares, tributary of the Tagus, the main Iberian watercourse, crossing the Spanish Southern *Meseta* from E to W (Fig. 2). The Sorbe has its source in the highest part of Sierra de Pela, a mountain range located in the eastern limit of the Central System Range at 1,500 m above mean sea level (amsl). The river runs southwards, first crossing gentle Paleozoic reliefs (schist, quartzite and slate) and marine Mesozoic carbonates before meeting the alluvial terrains of the Tertiary sediment infill of the Tagus basin, and finally joining the Henares River at a height of 710 m amsl. The archaeological site is located at an altitude of 826 m amsl and 11 to 13.5 m above the current riverbed, under a dolostone rock cliff, in an area where the Sorbe valley widens and the Quaternary fluvial and alluvial deposits become more frequent (Figs. 2 and 3B). The Quaternary deposits are described in the geological maps of the region^57,58^ and mainly consist of fluvial terraces, alluvial cones and slope deposits (Fig. 3A). According to these geological maps, there are fourteen Quaternary fluvial terraces, from + 6 to + 180–190 m above the current riverbed. Those below + 20 m are generally considered Upper Pleistocene and Holocene in other nearby areas of the Tagus basin and Duero basins^59,60^.

The Peña Capón site is located under an east–west oriented, 42 m high rock cliff, formed by Upper Cretaceous marine dolostone layers dipping to the south. The dolostone outcrops as part of a long hogback relief oriented to the NW–SE that surrounds the Paleozoic shales, schists and quartzites, as well as the Lower Triassic Buntsandstein facies, located to the north and west. The archaeological site is located 80 m away from the current riverbed, close to a narrowing of the valley excavated in the dolostone relief. Due to its location, the site is flooded by the Beleña reservoir waters for most of the year since a dam was constructed in 1982 (Supplementary Figs. S1–S2, S4–S7 and Supplementary Video 1).

## Results

### Stratigraphy, sedimentology and micromorphology

The geomorphological analysis of the site indicates that the accommodation space where the Upper Pleistocene sediments were deposited, at the foot of the rock wall, was created due to (1) the high slope angle of the dolostone running west to east and crossing this sector of the valley and (2) the differential erosion of a less compact marly and finely laminated layer at the base of the compact dolostone formation. Vertical dolostone layers parallel to the rock wall and several large gravitational blocks could have generated a sort of corridor where mostly fine-grained deposits accumulated and were protected from erosion, as it is also observed on the opposite riverbank (Supplementary Figs. S5–S6). The geomorphological map shows that both fluvial floods of Sorbe River and surface runoff on nearby alluvial fans could have played a role in the formation of the deposits (Fig. 3A).

The Peña Capon archaeological site covers a 30 m long and 5 to 8 m wide area of about 150 sq m along the foot of the dolostone rock wall (Fig. 3B; Supplementary Fig. S5). The deposit slopes 4.5° to the west, perpendicular to the direction of the valley. The maximum thickness of the deposit recorded in the archaeological excavations is 0.95 m.

**Figure 4:**
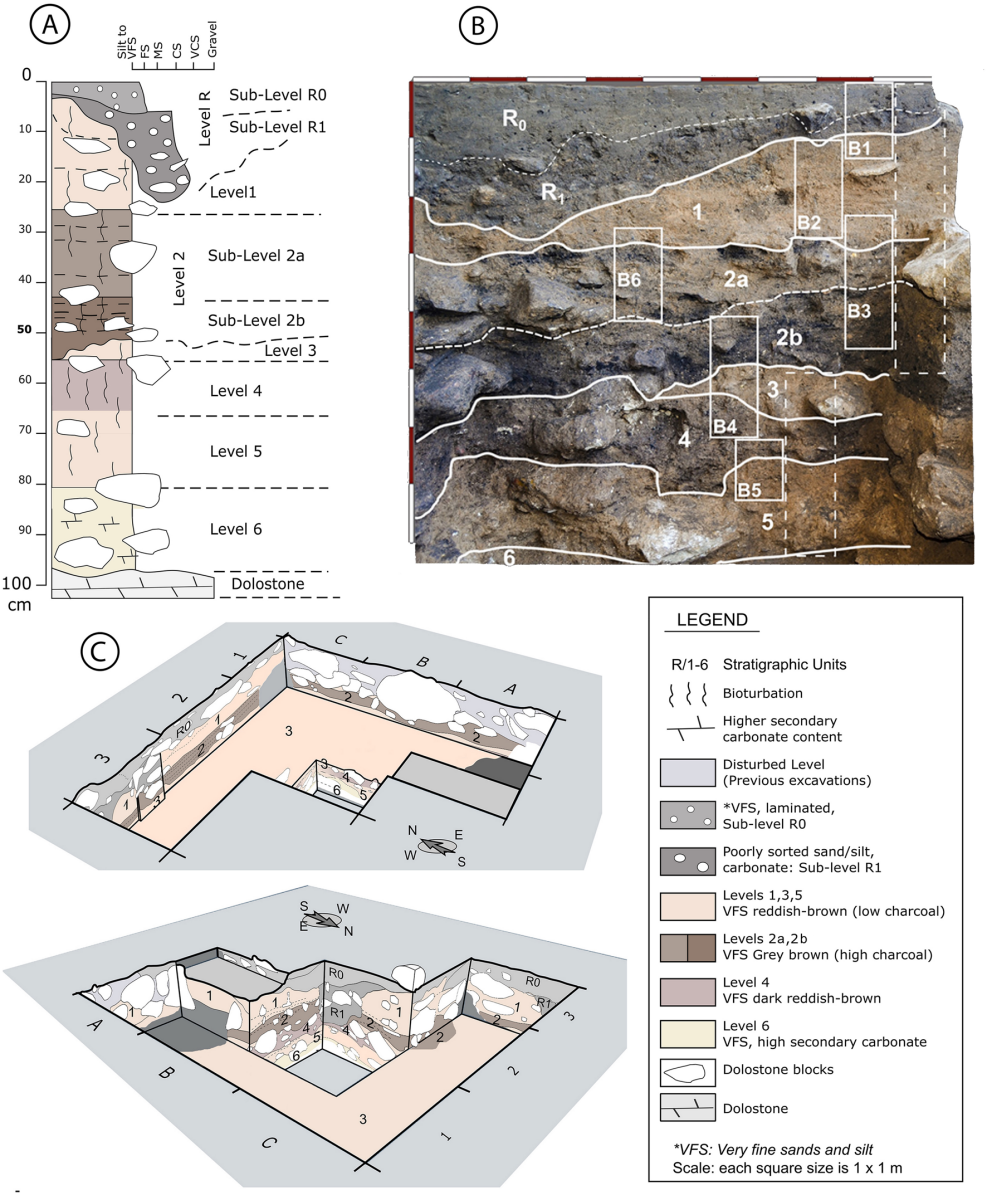
(**A**) Stratigraphic units defined in the Peña Capón site. Note that the main differences between layers are due to variations in organic matter and secondary carbonate content. (**B**) Stratigraphic sequence recorded in the western profile of square 2B showing sample location for micromorphology (B1–B5) and sedimentology (dashed-lines rectangles). (**C**) Views of the excavation profiles at Peña Capón showing the different distribution and geometry of the stratigraphic units defined in the site.

From a sedimentological point of view, the archaeological deposit consists of fine sand and silt intermixed with rock fragments showing a mixture of natural and anthropogenic sediment components, including varying amounts of lithics, charcoal and bones. Rock fragments consist of subangular dolostone derived from the rock shelter wall and sub-rounded to rounded quartzite and fine siltstone gravel. Few small angular pieces of chert, rock crystal and quartzite represent by-products of tool production^61^. The stratigraphic sequence is composed of six different sedimentological units containing archaeological remains (Levels 1 to 6), defined mainly on the basis of sediment color variation (Fig. 4). An overlying sedimentary unit of heterogeneous dark grey sandy loam, containing mixed archaeological remains including few pottery sherds, unconformably covers the Pleistocene archaeological deposit. This layer has been named R and can be roughly subdivided into a darker (Munsell Color Code 0,7Y 5/2), more coarse-grained lower one (R1) and the lighter-colored (0,4Y 5/2), finer-grained upper one (R0). The layers of the archaeological deposit are quite homogenous, sharing many common sedimentological and micromorphological features but varying in color due to their different content in burned components and carbonate. Levels 1, 3, 5 and 6 are light orange-brown to reddish-brown (9YR 5/3), while levels 2 and 4 are darker grey-brown (10YR 5/2, 10YR 5/3). Levels 1 and 3 are very similar, corresponding to mainly homogeneous fine sand and silt layers. Level 1, thicker, shows some lamination in its lower part. Level 3 thins eastward and has a sharp contact with the overlying level 2 and diffuse transition to the underlying level 4. Level 2 is divided into two sub-units, 2a and 2b. 2a on top is grey-brown and contains charcoal-rich lenses with abundant bone fragments; 2b below, is darker and shows higher contents of charcoal and charred organic matter. Its lower contact is irregular. Level 2b includes some small pits and a possible micromammal burrowing. The color of level 4 ranges from reddish brown to a more intense reddish tone (10YR 5/2 to 5YR 5/3), suggesting rubefaction processes. Levels 5 and 6, in the lower part of the sequence, are lighter in color (10YR 7/3) and richer in secondary carbonate.

**Figure 5:**
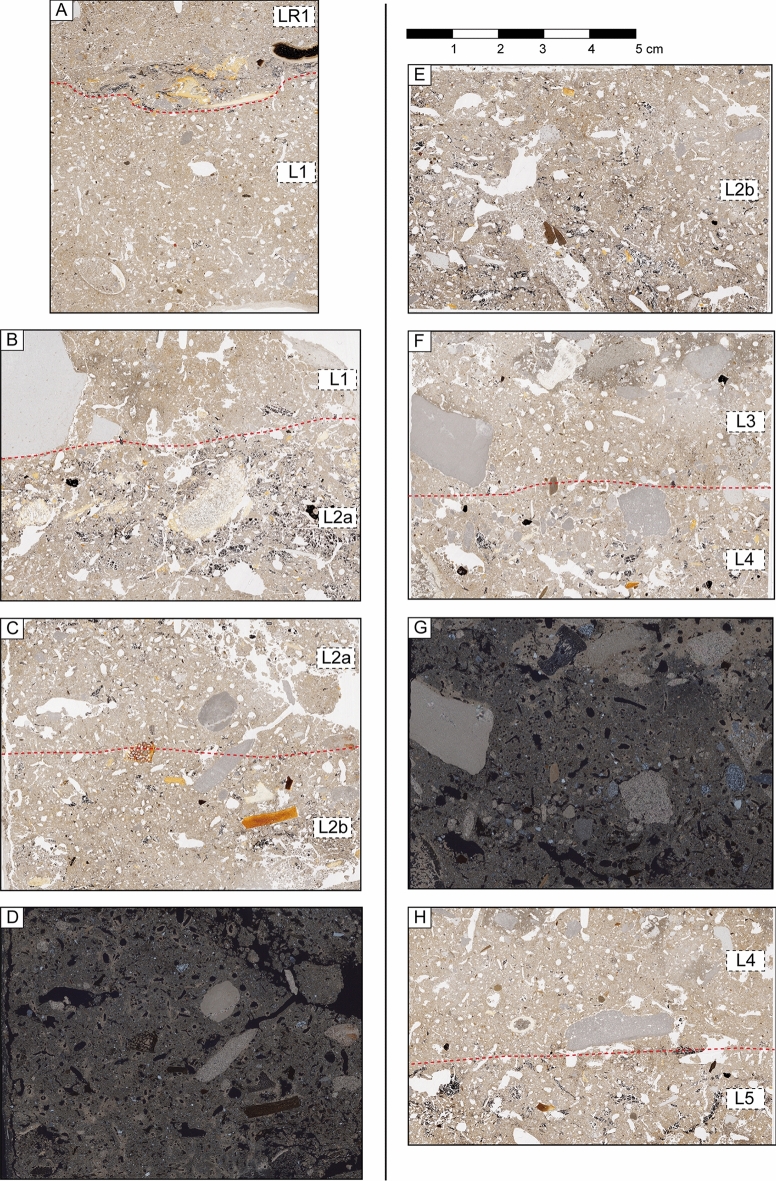
Flatbed scans (1200 dpi) of selected thin sections from the sediment sequence at Peña Capón. (**A**) Erosional contact between level R1 (LR1) characterized by strong compaction and a platy microstructure and level 1 (L1) showing less compaction and a channel microstructure. At the interface, a sediment lens rich in charcoal and bone is present (thin section PCPN 1.2). The dashed line is the boundary between the two levels. (**B**) Thin section PCPN 6.1 with the interface between levels L1 and L2a. At the sampling location, the upper part of L2a consists of a 2 cm thick band rich in charcoal and bone fragments. (**C**) Thin section PNCP 3.2 showing the gradual transition between levels 2a and 2b. (**D**) Same as C but captured under XPL. Note abundant calcite hypocoatings around biopores. (**E**) Thin section PNCP 4.1 from level L2b with several large biopores partly refilled with granules. (**F**) The interface between levels L3 and L4, which is delineated by a thin layer of fine gravel. The light-colored L3 shows diffuse impregnation with secondary calcite. (**G**) Same as E, but captured under XPL. Secondary carbonate is indicated by high birefringence. (**H**) Thin section PNCP 3.2 showing the transition from level 4 to level 5.

The granulometric analyses of the sediment sequence display a homogeneous textural composition dominated by poorly sorted very fine sand and coarse silt, with a low proportion of clay-size particles (maximum of 10%) (Fig. S19). The carbonate content ranges from 10 to 32% with an increase in the lower levels. Total Organic Carbon (TOC) values range from 0.3 to 2.9% and match with the color change, indicating that this is mainly related to variation in organic matter content. Thin sections show that charcoal with well-preserved cell structures is common in dark-colored levels, where it occurs together with amorphous charred organic matter of unknown origin (Fig. 5). High values in magnetic susceptibility, χlf, also occur in the dark-colored, organic matter-rich levels R1, 2b and 4, i.e. layers with high amounts of charred organics. Sediment generally has a low degree of compaction, related to presence of abundant pores consisting of biogenic channels and burrows formed by roots or soil-dwelling mesofauna^62^ creating bioturbation on a microscale. Geometry and thickness of the stratigraphic levels are represented in Fig. 4, and detailed results of sedimentology and micromorphology are included in Supplementary Texts S4 and S5 and Supplementary Table S2.

### Radiocarbon dating and Bayesian modeling

The chronological setting of the Peña Capón sequence is based on radiocarbon dating. Selected samples are faunal remains with anthropogenic modifications (mostly cut marks) (n = 21) and charcoal fragments (n = 12) recovered from secure stratigraphic contexts and covering the whole sequence excavated thus far. These samples were first identified to taxon (when possible) and then sent to two different laboratories for cross-checking results: 15 samples were sent to the CologneAMS centre at the University of Cologne and 18 samples to the Oxford Radiocarbon Accelerator Unit (ORAU) at the University of Oxford. Out of 33 samples we obtained 22 AMS reliable determinations and found no significant differences between results provided by both labs. One charcoal sample showed a modern age, while eight bones and two charcoals, mostly from levels 4 to 6, failed due to low, very low or no yield (Supplementary Table S3).

**Figure 6:**
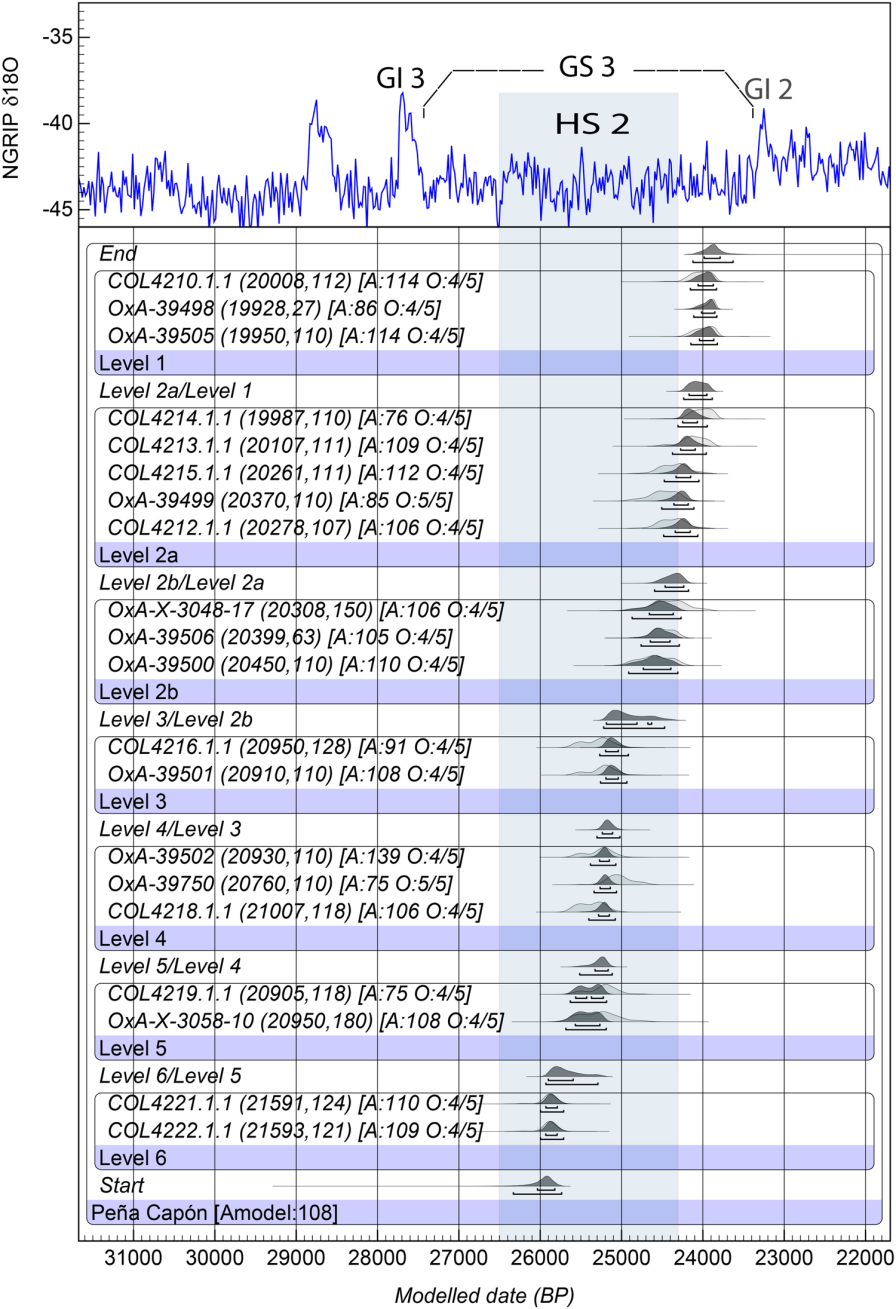
Bayesian Final Model (2) for the Peña Capón sequence showing Probability Distribution Functions (PDFs) for all radiocarbon determinations and boundaries between archaeological levels. Results are plotted against the δ18O record of the NGRIP ice core, indicating Greenland Interstadials 3 and 2 (GI 2 & GI 3), Greenland Stadial 3 (GS 3)^67^, and the chronology of Heinrich Stadial 2 (blue bar)^68^. 14C dates are shown in parentheses, and Agreement indexes and Outliers’ prior and posterior probabilities are shown in square brackets. Calibration of dates and Bayesian modeling were calculated using OxCal 4.4 online software (https://c14.arch.ox.ac.uk/oxcal.html).

With the aim of building a strong probabilistic framework for the sequence of human occupations recorded at Peña Capón we constructed a Bayesian model based on obtained radiocarbon determinations. As lengthy applied and discussed in the last years, if properly devised, Bayesian modeling is an accurate and informative way to integrate radiometric data with stratigraphically recorded archaeological evidence to create reliable chronological models, and thus improve chronometric precision, mitigating uncertainties and eliminating outliers^63–66^. A preliminary model (Model 1), containing all radiocarbon dates, showed an A_model_ of 53.2, thus pointing to potential problems in the relation between the prior and posterior distributions (i.e. between the unmodeled dates and their location within the sequence on the one hand, and the modeled calibrated results on the other). One date from level 3 (COL4217.1.1) and one from level 5 (OxA-39749) showed agreement indexes < 60% and posterior probabilities of being outliers of 81% and 14% respectively (Supplementary Table S4 and Fig. S21). Hence, these dates were not included in the final model (Model 2). This model, composed of 19 radiocarbon dates, is presented in Fig. 6 and Supplementary Table S5, and shows individual agreement values ranging between 139.3 and 74.7, an A_model_ of 107.6, and posterior probabilities of ≤ 5% for all determinations. The consistency of Model 2 is very high, as it shows an excellent degree of agreement between prior information, radiocarbon determinations and posterior probabilities, thus confirming geoarchaeological interpretation on the site’s stratigraphic integrity based on sedimentology and micromorphology.

The model results allow us to place the start boundary of the Peña Capón sequence excavated thus far (base of level 6) between 26.3 and 25.7 ka cal BP at 95.4% probability, and the end boundary (top of the known sequence) at 24.1–23.6 ka cal BP. However, when considering the results of applying the ‘date’ command in OxCal, the Probability Distribution Function (PDF) for the time span of human occupation at the site is constrained between 26.1 and 23.9 ka cal BP. The calendar age estimates for each level are shown in Table 1 and their associated PDFs are provided in Supplementary Fig. S22 (complete data and boundaries between levels are shown in Supplemaentary Table S5).

**Table 1:** Calendar age estimates for each archaeological level at Peña Capón, based on the Bayesian Model 2 (Fig. 6) as calculated by the ‘date’ command in OxCal 4.4 online software (https://c14.arch.ox.ac.uk/oxcal.html).

Level (phase)	Duration (cal BP)			
	68.2% probability		95.4% probability	
*Peña Capón*	*25,770*	*24,110*	*25,954*	*23,874*
1 (Solutrean)	24,064	23,868	24,190	23,784
2a (Solutrean)	24,330	24,092	24,460	23,968
2b (Solutrean)	24,792	24,370	25,046	24,282
3 (Solutrean)	25,210	24,970	25,264	24,668
4 (Pre-Solutrean)	25,280	25,140	25,406	25,068
5 (Pre-Solutrean)	25,616	25,260	25,798	25,192
6 (Pre-Solutrean)	25,962	25,722	26,130	25,448

Associated PDFs are shown in Fig. S22.

These results confirm the high sedimentation rate of the deposit^25,26^, where few more than 2,000 years are recorded in 95 cm. This explains the overlap between some dates (both unmodeled and modeled) obtained in adjacent levels, which is hence not related to post-depositional mixing—as also shown by sedimentology and micromorphology—but to the standard deviations of radiocarbon dates.

Concerning the cultural sequence, the Solutrean at Peña Capón is first recorded at ∼25.3 ka BP and lasts until ∼23.8 ka BP. In turn, the first modern human presence recorded thus far, associated in level 6 to pre-Solutrean assemblages (either Gravettian or Proto-Solutrean), is detected at ∼26.1 ka BP. Both episodes started and developed during Greenland Stadial 3 (GS 3), a stadial phase within the LGM^69^ and, more significantly, both were triggered during the rapid cooling of Heinrich Stadial 2 (HS 2) (Fig. 6). In fact, the whole pre-Solutrean occupation, and most of the Solutrean one, were developed during HS 2. This bears relevant implications for understanding human-climate-environment interactions during the first settlement of the Iberian interior by modern humans, as will be discussed below.

### Pollen

**Figure 7:**
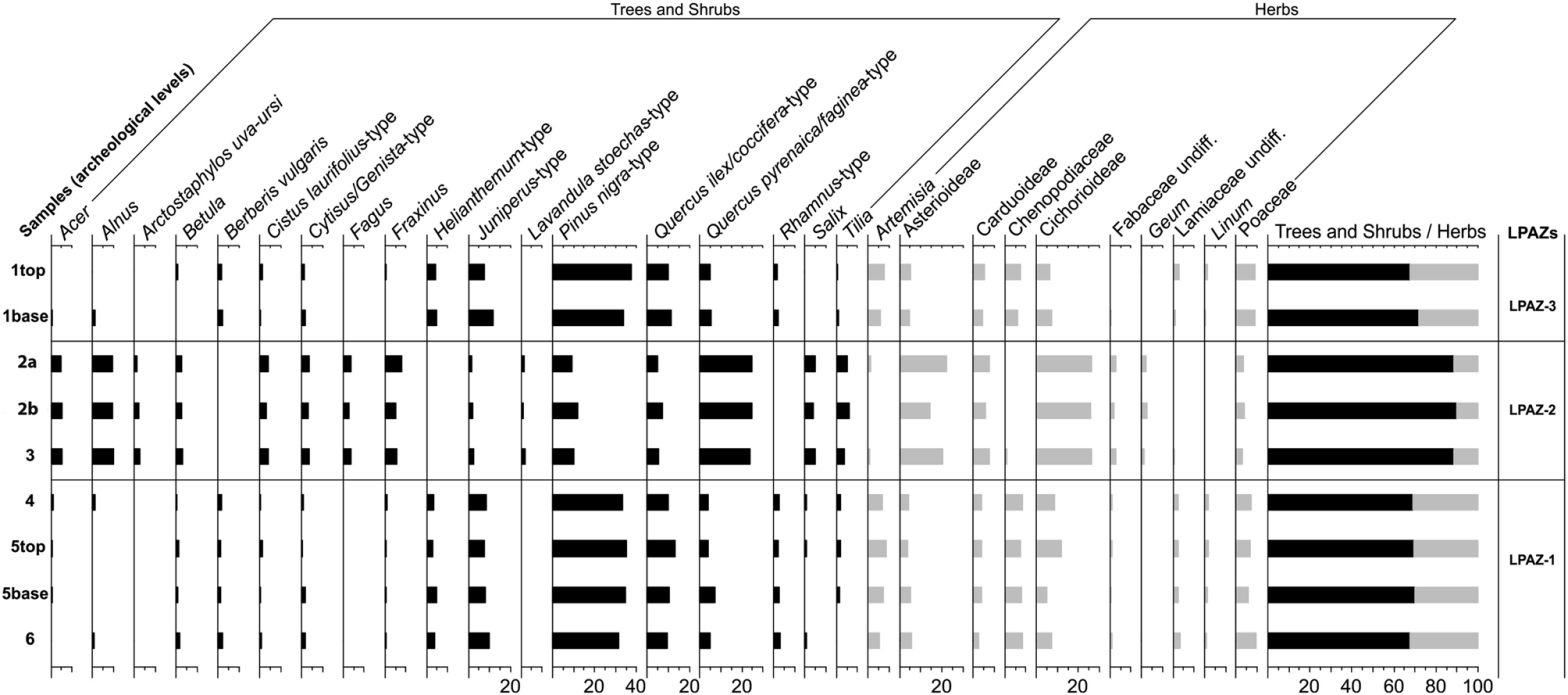
Percentage pollen diagram from the Peña Capón sequence.

For the Peña Capón sequence, 9 pollen spectra were analyzed and 28 taxa were identified. To facilitate description and interpretation of the pollen diagram with respect to vegetational changes, three Local Pollen Assemblage Zones (LPAZs) were established (Fig. 7). These zones denote significant changes in the pollen composition and represent major changes in vegetation. LPAZ-1 is largely dominated by *Pinus nigra* (31.5–35.2%) indicating a Spanish black pine forest in the vicinity^70–72^, which represents trees that, nowadays, grow in the higher mountains of Mediterranean central Iberia under supramediterranean climatic conditions (mean annual temperature of 8.13 °C and 400–1000 mm of annual rainfall)^73,74^. *Quercus ilex/coccifera* (9.4–13.1%) and *Juniperus* (7.4–9.9%) show continuous high values, concomitant with those of *Berberis vulgaris* (1.1–2.2%), *Linum* (1.1–1.7%) and *Rhamnus* (2.3–3.3%), suggesting the regional presence of holm oak (*Quercus ilex* subp. *rotundifolia*) and juniper (*Juniperus thurifera*) woodlands^75,76^. Mesophilous trees such as *Quercus pyrenaica/faginea*, *Acer*, *Alnus*, *Betula*, *Fraxinus*, *Salix* and *Tilia* are usually low (< 5%) and beech (*Fagus*) is absent. This zone shows the highest percentages of herbs (30.1–32.6%), mainly represented by cryoxerophytic taxa (*Artemisia* 5.5–8.5%, Chenopodiaceae 7.4–8.5%) and heliophilous/cryophilous herbs (Poaceae 5.9–9.4%), probably indicating cold and dry conditions, as also shown by cryoxerophytic shrubs, such as *Helianthemum* (2.8–4.6%). Anthropogenic-zoophilous pollen taxa (Asterioideae, Carduoideae, Cichorioideae) present relatively low values that do not exceed 12%.

LPAZ-2 is characterized by synchronous increases of mesophilous taxa such as *Quercus pyrenaica/faginea* (23.8–25%), *Acer* (5–5.5%), *Alnus* (9.3–9.8%), *Betula* (2.7–3%), *Fraxinus* (5.3–7.9%), *Salix* (4–5%) and *Tilia* (3.7–6%), and the first appearance in the pollen diagram of *Fagus sylvatica* (2.7–3.7%). These results suggest (i) denser deciduous oak woodlands (trees and shrubs values between 88.4 and 90%), and (ii) the first arrival of beech near the site as a result of climatic warming and increased humidity^77^. Thus, the Iberian Central System is confirmed as a refuge zone for this taxon during much of the Late Pleistocene, including the LGM^78–80^, as has been also documented in other mountains in northern Iberia^81,82^. This finding represents the first record of beech from the LGM in the Iberian Central System. *Pinus nigra*, *Quercus ilex/coccifera* and *Juniperus* decrease, as well as cryoxerophytic (*Artemisia*, Chenopodiaceae) and heliophilous/cryophilous (Poaceae) taxa, while *Helianthemum*, *Berberis vulgaris*, *Linum* and *Rhamnus* disappear. Shrub and herb taxa percentages related to deciduous oak woods (*Arctostaphylos uva-ursi* 1.4–2.4%, *Cistus laurifolius* 3.3–4.3%, *Cytisus/Genista* 3.3–3.7%, *Geum* 1.2–1.7%, *Lavandula stoechas* 1.3–1.8%) experience an increasing tendency, while anthropogenic-zoophilous pollen taxa (Asterioideae 14.7–22.1%, Carduoideae 6–7.9%, Cichorioideae 26–26.4%) are much more abundant.

Finally, LPAZ-3 shows a similar pattern to that of LPAZ-1. *Pinus nigra* is at its maximum in this pollen sequence (37.3%). Other components increase such as *Quercus ilex/coccifera* (10.1–11.6%), *Juniperus* (7.7–11.6%), *Berberis vulgaris* (1.8–2.2%), *Rhamnus* (1.8–2.2%), *Artemisia* (6.1–7.7%), Chenopodiaceae (6.1–7.7%), *Helianthemum* (4.1–5%), *Linum* (0.6–1.1%) and Poaceae (8.8–8.9%), while some tree or shrub taxa (*Acer*, *Alnus*, *Betula*, *Cistus laurifolius*, *Cytisus/Genista*, *Fraxinus*, *Quercus pyrenaica/faginea*, *Tilia*) abruptly decrease and some even disappear (*Fagus*, *Salix*), as do *Arctostaphylos uva-ursi*, *Lavandula stoechas* and *Geum*. Anthropogenic-zoophilous taxa (Asterioideae, Carduoideae, Cichorioideae) also decrease.

### Wood charcoal

Charcoal remains were mostly scattered throughout the excavated area, besides some concentrations, including a fireplace in level 2a (Supplementary Figs. S12–S13). In general terms, carbonized wood found at Peña Capón is scarce in relation to the number of fragments recovered. Even in the flotation samples, where all charcoal remains present in the sediment are recovered, the number of identified fragments is low, limited in many cases to just one fragment, being most of them smaller than 2 cm. These samples mostly included black and ash-gray sediment devoid of charcoal input.

Among the 154 identified wood fragments, taxonomic diversity is low (Table 2). Most of the fragments have been assigned to either *Salix* sp. or *Juniperus* sp., being the rest taxa (*Alnus* sp., *Cistus* sp., *Fraxinus* sp., Leguminosae, *Rhamnus/Phillyrea*, Rosaceae) represented by a low number of fragments. Moreover, a large number of remains could only be identified as angiosperms dicotyledons or gimnosperms (conifers) due to poor preservation or small size. A total number of 52 remains were unidentifiable.

**Table 2:** Wood charcoal identified in Peña Capón.

Taxon/level	1	2a	2b	3	4	5	6
*Alnus* sp			1				
*Cistus* sp	1	1					
cf. *Cistus*	2		2				
*Fraxinus* sp	1		4				
*Juniperus* sp	2		5			4	25
Leguminosae		1					
*Rhammus/Phillyrea*							1
Rosacea tp*maloidea﻿*							1
cf. Rosaceae (tp *maloidea*)						1	
*Salix* sp	7	1	3		11	31	
cf. *Salix*						3	
Ang. no id	13	6	9	2		1	1
Gim. no id		2	1		1		1
Total	26	11	25	2	12	40	29
Unidentif.	5	3	15		1	1	27

Unlike other proxies studied at the site –especially pollen– wood charcoal analysis do not show significant environmental differences throughout the sequence. Thus, charcoal results point to a recurrent pattern of wood procurement from level 5 to 1, being level 6 the only episode where a different behavior is attested (Table 2). This level has shown the higher number of *Juniperus* sp. (*juniper*/*sabina*) fragments, together with one remain of *Rhamnus/Phillyrea* and one of Rosaceae (*Maloideae* type), while *Salix* is totally absent. In contrast, in levels 5 and 4 *Salix* wood is the most represented, and *Juniperus* is represented only by 4 fragments in level 5.

It is noteworthy that thicker and more extensively excavated levels (1, 2a, 2b and 3) (Fig. 4) present relatively lower amounts of charcoal remains compared to levels excavated only in the 1 sq meter test pit (4, 5 and 6). Thus, in these levels, corresponding to the Solutrean human occupations, the number of identified fragments is scarce, and a relevant number of them have been assigned to unidentifiable angiosperms, mostly due to their small size and their deformed and altered conditions. However, levels 1, 2a and 2b show the greatest diversity within the sequence, including taxa such as *Alnus*, *Cistus*, *Fraxinus* or Leguminosae (although limited in most cases to isolated fragments) together with *Salix* and *Juniperus* (Table 2).

Although the low number of identified charcoals calls for some caution concerning the palaeoenvironmental interpretation of these results, some conclusions can be drawn from them. *Juniperus* sp, present in levels 1, 2b, 5 and especially 6, is a little tree or shrub typical of open landscapes, and some species within this genus, such as *sabina*, cope well with low temperatures and rocky floors. The rest of identified taxa are mainly riverbank species, including *Salix*, *Alnus* and *Fraxinus*, which could point to a greater availability of these species in the surrounding area. Lastly, *Cistus*, Leguminosae and *Juniperus* point to shrub-dominated landscapes.

### Microvertebrates

**Figure 8:**
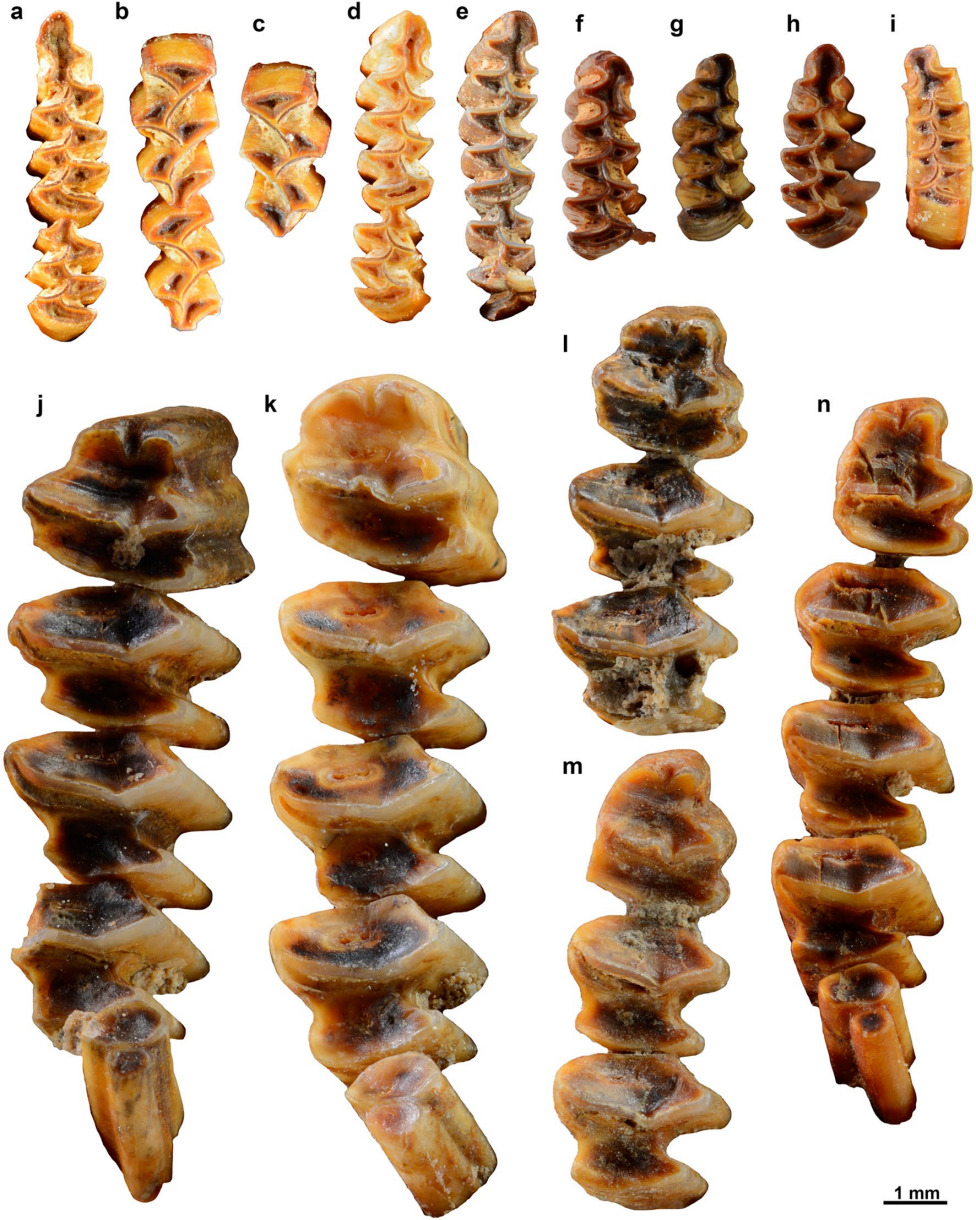
Photographs of the occlusal surface of molars from selected species of small mammals collected at Peña Capón levels 1–6. (**a**) left m1, m2 of *Microtus arvalis* (level 1); (**b**) M1, M2 of *Microtus arvalis*-*M. agrestis* (level 2a); (**c**) upper M1 of *Arvicolinae* (level 2a); (**d**) right m1, m2 of *Microtus agrestis* (level 2a); (**e**) right m1, m2 of *Microtus agrestis* (level 2a); (**f**) m1d of *Microtus arvalis* (level 2a); (**g**) m1d of *Microtus agrestis*, juvenile (level 2a); (**h**) *Microtus arvalis* (level 2b); (**i**) *Microtus arvalis* (level 3); (**j**)–(**n**) lower molars of lagomorphs (**j**–**l** level 1; **m** level 3; **n** level 5).

The small vertebrate assemblage identified at Peña Capón accounts for one of the few microfossil collections associated to the LGM time span recorded to date in Iberia^83–85^, being the only one in the whole Iberian hinterland. A total of 226 samples, collected throughout the whole sequence of human occupation at the site, have been analyzed. Of them, 205 contained identifiable remains, among which we determined fishes, amphibians, snakes, birds, insectivores, lagomorphs and rodents. Only lagomorphs and rodents have been determined to the species level (Table 3 and Fig. 8). Among the lagomorphs, the species *Oryctolagus cuniculus*, the field rabbit or European rabbit, dominates (see also Macrovertebrates section below). The dominant species of rodents is *Microtus arvalis*, the common vole, while other species are poorly represented. These are *Eliomys quercinus*, the dormouse; indeterminate species of the genera *Apodemus*, the field mouse; *Terricola*, the “microtus” group of species with pitymyan rhombus; and *Microtus agrestis*, the field vole or short-tailed vole. The complete microvertebate taxonomic association per stratigraphic level is presented in Table 3 according to the NISP (number of identified specimens).

**Table 3:** Microfossil remains (NISP) identified in Peña Capón.

Taxon/level	1	2a	2b	3	4	5	6
Fishes		5	1	2			
Amphibians	1	5		1			
Serpentes		1				1	
Birds	11	14	2	3			2
Insectivores		2					
Rodentia indet	2	5		4			
*Eliomys quercinus*	1						
*Apodemus*	2						
*Microtus*	2	8		1	1		
*Microtus arvalis*		6	8			2	
*Microtus agrestis*	2	1	1	1			2
*Terricola*			7				
#Taxa (S)	7	9	5	6	1	2	2
#Samples	50	72	45	20	9	20	10

“#Samples” refers to the number of plastic bags collected for the study. “#Taxa (S)” is the number of taxa identified in the total number of samples for each level.

*Microtus arvalis* is an Arvicolinae (Cricetidae) that currently lives in temperate Europe and some regions of western Asia. It is a strictly herbivorous species inhabiting open meadows, and it is well adapted to cold conditions, as shown by its survival to the climatic changes of the LGM. Local extinctions of *M. arvalis* have been recorded in northern and central Europe, as well as in Britain, due to post-LGM reforestation, which replaced the dominant open grassland areas present during the LGM^86^. Therefore, the significant presence of *M. arvalis* in levels 2a, 2b and 5 of Peña Capón points to environmental conditions that favored open and humid meadows, the species' favorite habitat.

More broadly, although samples from levels 4 to 6 have been only gathered in a 1 sq-meter test pit, thus biasing the sampling throughout the sequence (Figs. S24 & S25), the distribution of taxa and the biodiversity recorded in levels 1 to 6 at Peña Capón, points to levels 1, 2a and 3 as those presenting better climatic and habitat conditions for Mediterranean species and open spaces, such as wet meadows. In turn, the lower biodiversity found in levels 4 to 6, as measured by the low number of taxa (Table 3), and in general the lesser representation of micromammals in these layers, could point to harsher environments, as shown by the presence of *Microtus agrestis* in level 6.

### Macrovertebrates

Most of the 17,275 analyzed bone fragments throughout the Peña Capón sequence present a high degree of fragmentation, and hence only 2.85% of them were identified to taxon (Table 4). However, although 97.15% of remains are thus indeterminate, 11.9% of them could be assigned to either small, medium or large-sized animals (Table 4).

**Table 4:** Taxonomic representation of macrovertebrates remains at the Peña Capón sequence.

Taxon/level	1	%	2a	%	2b	%	3	%	4	%	5	%	6	%	Total
*Bos/Bison*		0.0	2	0.0	1	0.0		0.0		0.0		0.0		0.0	3
*Equus caballus*	7	0.2	39	0.6	20	0.4	11	0.8	2	1.3	13	0.9	4	6.8	96
*Cervus elaphus*	2	0.1	20	0.3	5	0.1	12	0.9	2	1.3		0.0		0.0	41
*Capreolus capreolus*	1	0.0		0.0		0		0.0		0.0		0.0		0.0	1
*Capra pyrenaica*	9	0.3	17	0.3	5	0.1	3	0.2		0.0	1	0.1		0.0	35
*Rupicapra pyrenaica*	2	0.1	8	0.1	1	0		0.0		0.0		0.0		0.0	11
*Meles meles*		0.0	4	0.1		0		0.0		0.0		0.0		0.0	4
*Felix silvestris*		0.0	1	0.0		0		0.0		0.0		0.0		0.0	1
Carnivore indet		0.0	1	0.0		0		0.0		0.0		0.0		0.0	1
*Oryctolagus cuniculus*	128	3.6	97	1.6	28	0.6	51	3.7	4	2.6	13	0.9	4	6.8	325
Indet. large size	43	1.2	59	1.0	35	0.8	24	1.8	16	10.6	4	0.3		0.0	181
Indet. medium size	89	2.5	144	2.3	77	1.7	70	5.1	7	4.6	13	0.9	1	1.7	401
Indet. small size	382	10.7	551	8.9	291	6.5	213	15.6	7	4.6	34	2.3	1	1.7	1479
Indetermined	2899	81.3	5218	84.6	4033	89.7	984	71.9	113	74.8	1400	94.7	49	83.1	14,696
Total	3562	100.0	6161	100.0	4496	100.0	1368	100.0	151	100.0	1478	100.0	59	100.0	17,275

Among the identified species, rabbit is the most abundant throughout the sequence, except in levels 5 and 6, where horse is equally represented (Tables 4 and 5). Concerning macrovertebrates, horse is the best-represented taxon according to the MNI, and it also dominates the NISP in levels 2a, 2b, 5 and 6. Iberian ibex is the most represented with respect to NISP in level 1, while red deer dominates level 3 and shows similar values to horse in level 4 (Table 5). Other identified species are the large bovid in levels 2a and 2b, roe deer in level 1, chamois in levels 1, 2a and 2b, and badger and wildcat in level 2a (Table 5). The three best-represented macrovertebrates –horse, red deer and Iberian ibex– are related to different landscapes, being horse mostly adapted to open environments, deer pointing to woods and ibex to rocky areas. However, the three of them are generalist animals, capable of adapting to a range of climatic conditions and are not typical of either cold or temperate environments. Thus, in the absence of dental wear and stable isotope analyses (both to be conducted soon), macromammal evidence is still uninformative in terms of reconstructing surrounding environments, mainly due to the low number of remains still collected for layers 3 to 6. However, when considered together with pollen and micromammal results, macrovertebrate evidence is consistent with the interpretation of level 2a and 2b as showing temperate and humid environments. This is supported by the presence of roe deer, wildcat and badger in these levels, as these species are adapted to wooded environments. Likewise, although available data for levels 5 and 6 is still sparse, the relatively higher presence of horse, together with the decrease in the number of rabbits, is consistent with the existence of dry environments and open landscapes as shown by the pollen and micromammal analyses for these levels.

**Table 5:** Taxonomic representation of macrovertebrates remains at the Peña Capón sequence according to NISP and MNI.

NISP/level	1	%	2a	%	2b	%	3	%	4	%	5	%	6	%	Total
*Bos/Bison*		0.0	2	1.0	1	1.8		0.0		0.0		0.0		0.0	3
*Equus caballus*	7	4.7	39	20.1	20	36.4	11	14.3	2	25.0	13	48.1	4	50.0	96
*Cervus elaphus*	2	1.3	20	10.3	5	9.1	12	15.6	2	25.0		0.0		0.0	41
*Capreolus capreolus*	1	0.7		0.0		0.0		0.0		0.0		0.0		0.0	1
*Capra pyrenaica*	9	6.0	19	9.8	3	5.5	3	3.9		0.0	1	3.7		0.0	35
*Rupicapra pyrenaica*	2	1.3	8	4.1	1	1.8		0.0		0.0		0.0		0.0	11
*Meles meles*		0.0	4	2.1		0.0		0.0		0.0		0.0		0.0	4
*Felix silvestris*		0.0	1	0.5		0.0		0.0		0.0		0.0		0.0	1
Carnivore indet		0.0	1	0.5		0.0		0.0		0.0		0.0		0.0	1
*Oryctolagus cuniculus*	123	85.9	97	51.5	25	45.5	51	66.2	4	50.0	13	48.1	4	50.0	325
Total	144	100.0	191	100.0	55	100.0	77	100.0	8	100.0	27	100.0	8	100.0	518

MNI	1	%	2a	%	2b	%	3	%	4	%	5	%	6	%	Total
*Bos/Bison*		0.0	1	4.8	1	9.1		0.0		0.0		0.0		0.0	2
*Equus caballus*	1	7.7	3	14.3	3	27.3	2	33.3	1	33.3	1	33.3	4	80.0	15
*Cervus elaphus*	1	7.7	2	9.5	1	9.1	1	16.7	1	33.3		0.0		0.0	6
*Capreolus capreolus*	1	7.7		0.0		0.0		0.0		0.0		0.0		0.0	1
*Capra pyrenaica*	1	7.7	1	4.8	2	18.2	1	16.7		0.0	1	33.3		0.0	6
*Rupicapra pyrenaica*	1	7.7	4	19.0	1	9.1		0.0		0.0		0.0		0.0	6
*Meles meles*		0.0	1	4.8		0.0		0.0		0.0		0.0		0.0	1
*Felix silvestris*		0.0	1	4.8		0.0		0.0		0.0		0.0		0.0	1
*Oryctolagus cuniculus*	8	61.5	8	38.1	3	27.3	2	33.3	1	33.3	1	33.3	1	20.0	22
Total	13	100.0	21	100.0	11	100.0	6	100.0	3	100.0	3	100.0	4	100.0	60

Overall, these patterns are consistent with those indicated by the study of faunal assemblages from level II (Solutrean) and level III (Proto-Solutrean) as defined in the 1972 excavation (Supplementary Text S1), where the preferred hunted animals were also horses, red deer and ibex^25,87^. Stable isotopes obtained from herbivore teeth from those levels pointed to warm climate and temperate environments around ∼24 ka cal BP^87^, which also fits current palaeoenvironmental evidence for levels 2a and 2b, but not for level 1 as especially shown by pollen remains.

Mortality patterns and taphonomic analysis of the macrofaunal assemblages are provided in the Supplementary Information (Supplementary Text S7, Tables S6–S9, Fig. S26).

### Archaeological assemblages

**Figure 9:**
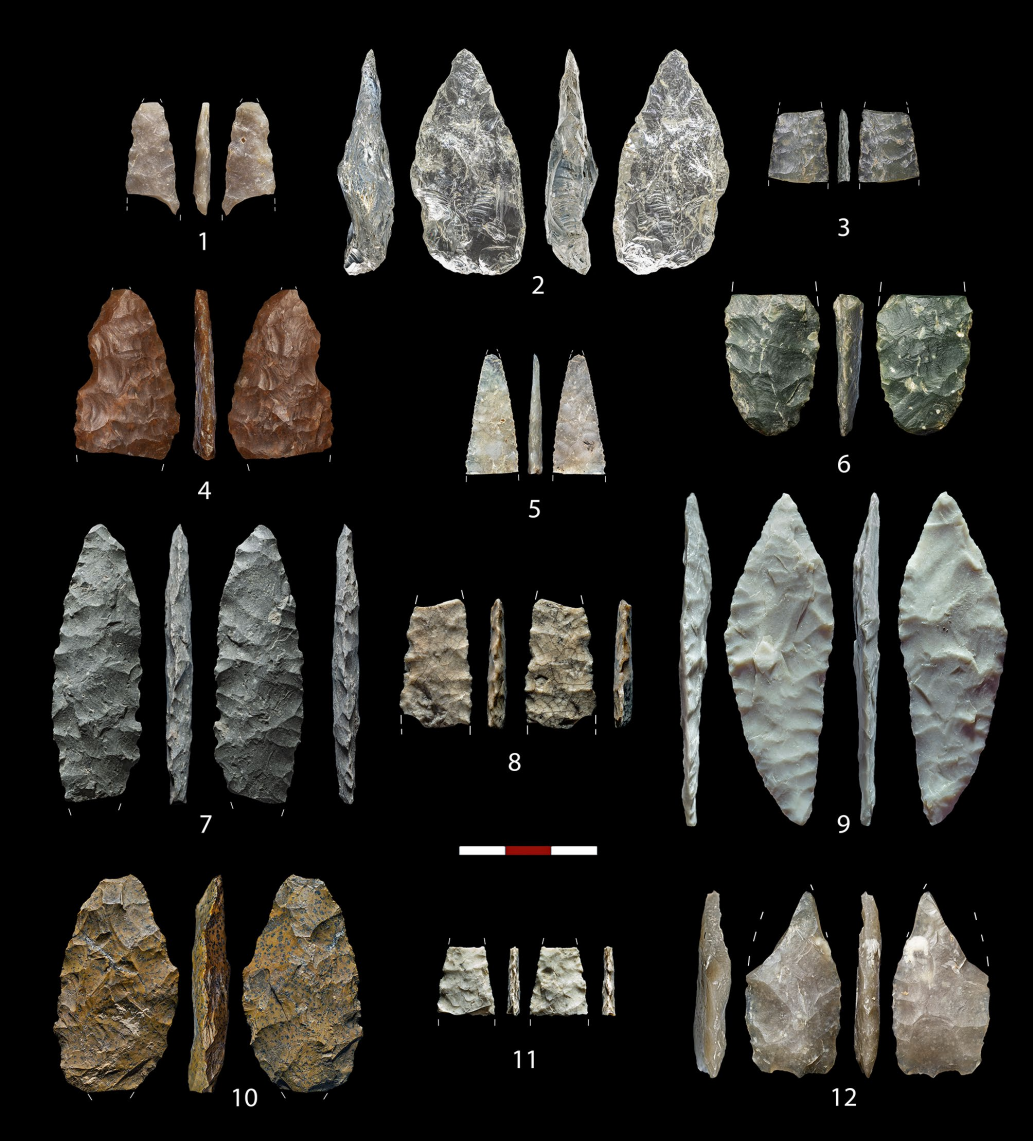
Solutrean lithic assemblages. Level 1: 1–3. Level 2a: 4–6. Level 2b: 7–9. Level 3: 10–12.

As previously reported^20,25,26,88^, Peña Capón hosts a sequence of recurrent occupations of hunter-gatherers bearing Solutrean and pre-Solutrean technocomplexes which, to date, has no parallel in the whole Iberian *Meseta*^43^. Together with anthropized faunal remains, a limited number of bone tools, ocher fragments and at least two fireplaces in levels 2a and 2b, lithics account for the most abundant archaeological material (Supplementary Figs. S8–S18). Foliate lithic armatures obtained through bifacial and unifacial invasive flat retouch are found throughout levels 1 to 3 (Fig. 9). Considering the classic typology-based chronological framework of the Solutrean^50,89–93^, the presence in all these levels of bifacial laurel leaf points and the absence of typically Upper Solutrean types, such as shouldered or barbed-and-tanged points (only recorded at Peña Capón as surface finds), enable us to place them in the Middle Solutrean (Supplementary Dataset 5). The dating of levels 1, 2a and 2b between 23.8 and 25.0 ka cal BP is roughly coherent with a Middle Solutrean attribution. However, level 3, dated between 24.7 and 25.3 ka cal BP, best fits the initial phase of the Solutrean. This partial incoherence supports the questioning of the traditional type-fossils as precise temporal markers^64,94,95^. Anyhow, typically Lower Solutrean types, such as *pointes à face plane*, are not found in the Peña Capón sequence, a circumstance that parallels the nearly absence of this phase/facies in central and southern Portugal, where the Proto-Solutrean is followed by the Middle Solutrean^64,93,96^.

**Figure 10:**
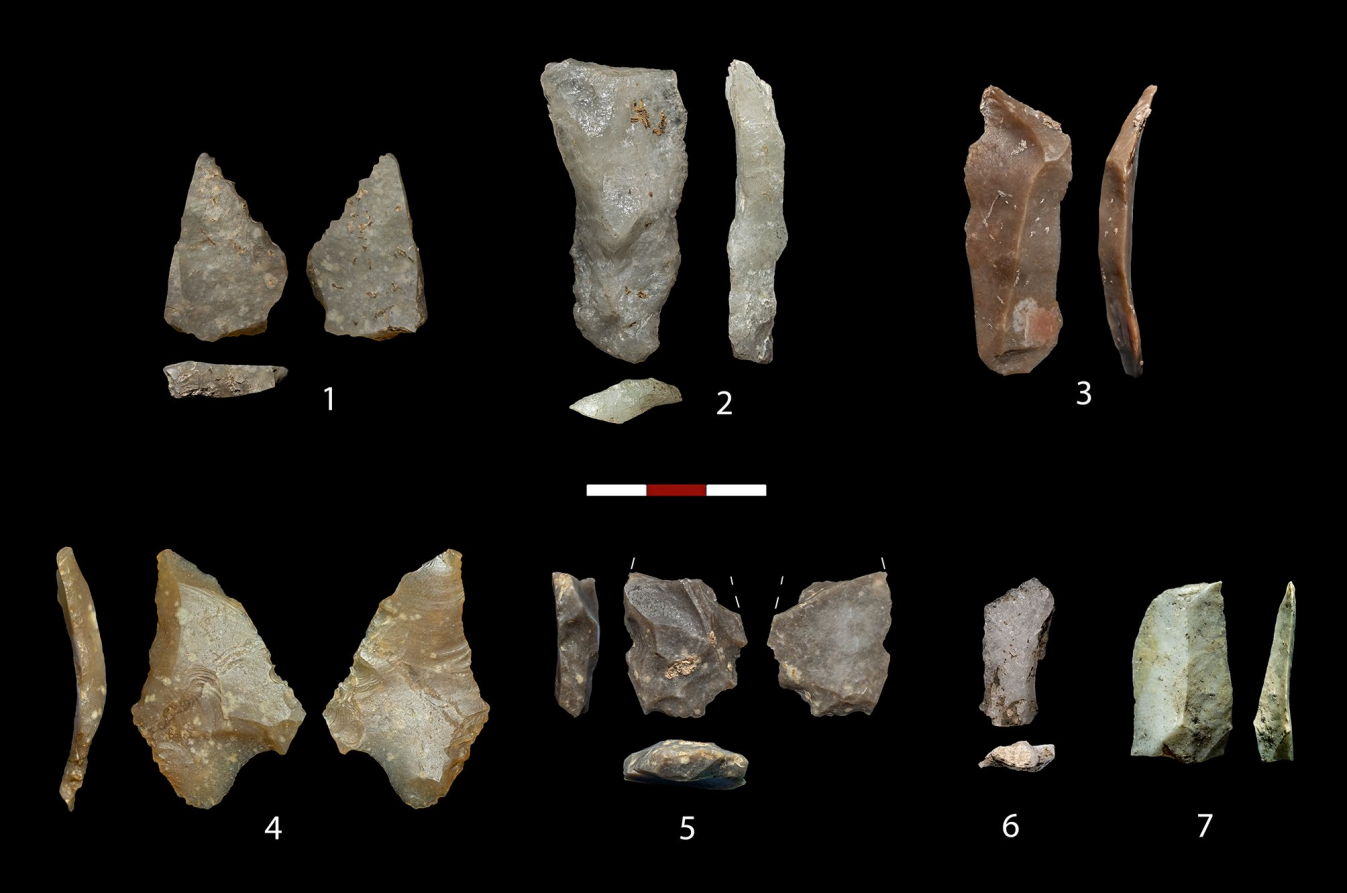
Pre-Solutrean lithic assemblages. Level 4: 1–3. Level 5: 4–5. Level 6: 6–7.

Since levels 4–6, dated between 25.1 and 26.1 ka cal BP, have been excavated in only 1 sq meter, their archaeological assemblages are still scarce. Although no index fossils or significant technological strategies have been recognized in these levels, the absence of foliate armatures, the higher presence of quartz and bladelets as compared to levels 1–3 (Supplementary Dataset 5), and their chrono-stratigraphic position, allow us to securely relate them to pre-Solutrean human occupations (Fig. 10). Based on the assemblages from the 1972 excavation at the site (Supplementary Text S1), a Proto-Solutrean component with Vale Comprido points (level III) (Supplementary Fig. S3), and a potentially Gravettian occupation (level IV) were described^20,25^. According to new radiocarbon determinations, these two levels (III and IV as defined in 1972) are currently dated to 25.6–24.9 ka cal BP and 26.3–25.7 ka cal BP (Supplementary Table S1), thus fairly mirroring levels 4–5 and level 6 of the new excavations respectively (Table 1 and Fig. 6). However, a fine-grained correlation between levels from the old and new excavations has not been possible to date, since no clear Proto-Solutrean or Gravettian traits have been identified in either level 4, 5 or 6, or a combination of them. Therefore, we prefer to provisionally describe archaeological assemblages of levels 4–6 just as “pre-Solutrean”. Yet, the presence of a Proto-Solutrean component at Peña Capón remains without doubt, and is now reinforced by obtained radiocarbon dates in levels 4–6 matching the Proto-Solutrean time span, as recorded in Portugal, between ∼26 and 25 ka cal BP^53,64,96^. Furthermore, this dating supports the triggering of the Proto-Solutrean as related to the rapid climate and environmental changes caused by HS 2, and especially to a decrease in vegetation cover and forest diversity^52–54^, as also reinforced by palaeoecological data obtained at Peña Capón.

## Discussion

### Site formation processes

The site has been previously interpreted as a result of the contribution of fluvial sedimentation and fallen blocks from the roof^26^. Fluvial origin is confirmed because of the homogeneity in grain size of the fine sediments, which suggests the lack of influence of debris flows coming from closer alluvial fans or slope deposits. The few sub-rounded to rounded quartzite and fine siltstone gravel found are consistent with the accumulation by fluvial transport. However, the downslope geometry of the deposit suggests that it is not that much a classic flat flood plain terrace deposit but the deposition during floods on the hill slope. The in situ preservation of the deposits can be explained by protection from reworking and erosion by the corridor formed between the rock wall and verticalized dolostone layers and big gravitational blocks in the external side of the site. The sediment low heterogeneity observed is just due to variations in organic matter and secondary carbonate content and not to changes in the sedimentary environment. The variation in χlf over some of the layers makes it more likely that the higher magnetic signal of the darker ones has been induced by burning. Besides fluvial deposition, physical disintegration of the rock shelter wall and gravitational processes have provided limited amounts of fines and frost-shattered debris contributing to accumulation of the archaeological sequence.

Apart from the erosion of the uppermost levels of the archaeological sequence, micromammal burrowing and local sediment disturbing in the top of level 3 and the lower part of level 2b, the main post-depositional processes are just the dissolution of primary carbonate grains and the intergranular precipitation of secondary carbonate, sometimes forming small nodules. These calcitic pedofeatures testify the lixiviation or carbonate leaching of larger blocks and fine sediments. The dominating channel and burrow microstructure testifies to intensive rooting and burrowing activity of mesofauna but only on a microscale. This bioturbation may have destroyed the primary depositional fabric of the fines but no signs of mixing between levels have been recorded. Overall, levels 5 to 1 rapidly accumulated over a period of about 2,000 years and represent an excellently preserved sediment sequence.

### Human–environment interactions and population dynamics during the HS 2 in the Iberian hinterland

Although the whole sequence of human occupation recorded to date at Peña Capón occurred during a stadial phase (GS-3), it included periods of both cold and relatively warm climates, corresponding to arid and more humid environments respectively. The warmest period corresponds to the bulk of the Solutrean occupations between 25.3 and 24.0 ka cal BP (levels 2a, 2b and 3), and is characterized by deciduous oak groves enriched with numerous mesophilous trees, including beech (LPAZ-2), and the presence of animals well-adapted to wooded environments, such as roe deer, wildcat and badger. This picture partially supports previous data from stable isotopes pointing to warm climate and temperate conditions at the site^87^, but it is now clear that this period was limited to part of the Solutrean (and not the Proto-Solutrean) and occurred well within GS-3 (and not during GI-2), including part of HS 2.

**Figure 11:**
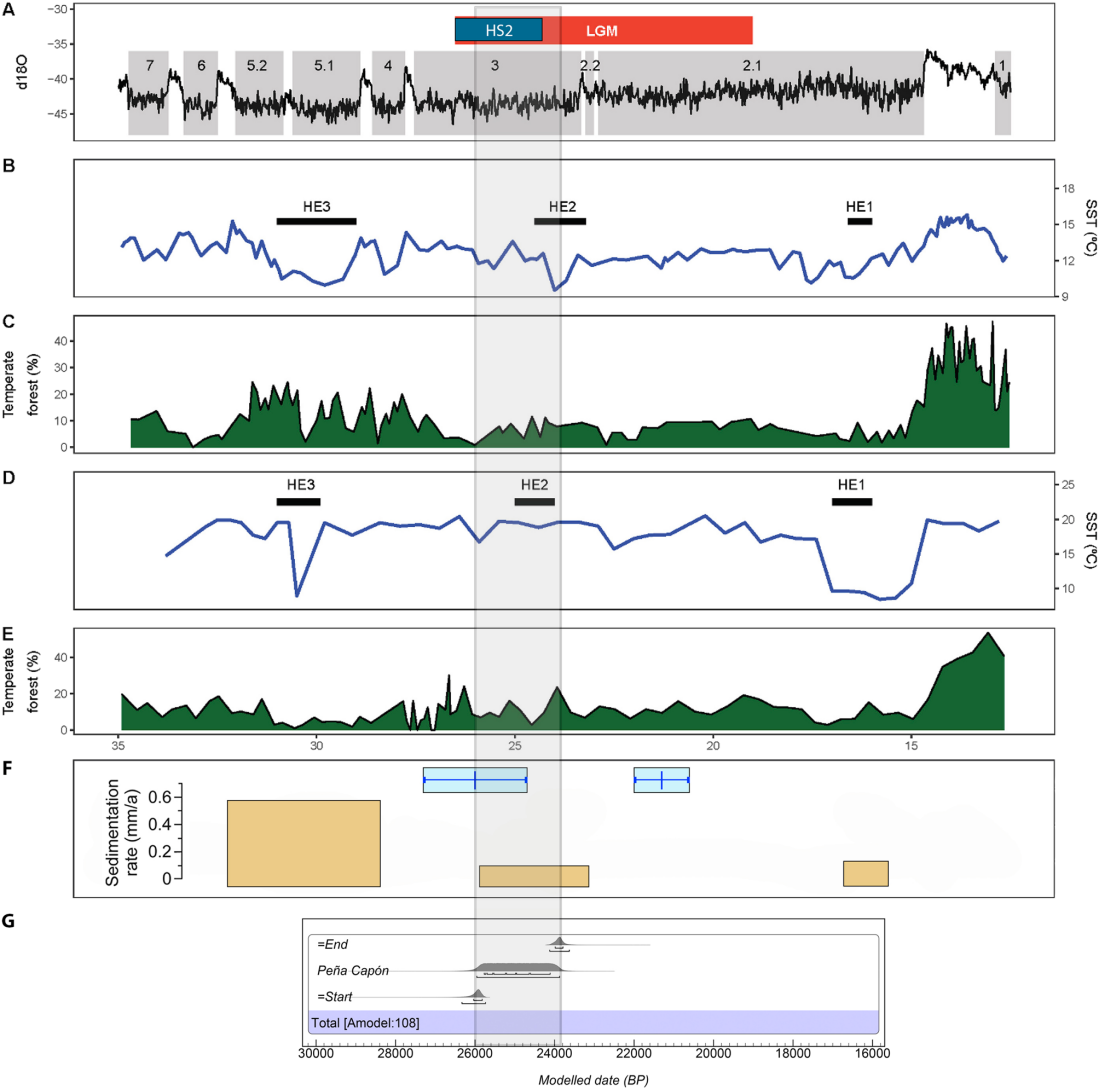
Global and regional Iberian climatic/environmental proxies from ∼35 to 12.5 ka cal BP (modified from [^43^: fig. 1] and [^40^: fig. 3] in relation to the modeled sequence of human occupation recorded at Peña Capón. A: δ18O record of the NGRIP ice core, with numbers and grey bars referring to Greenland Stadials^67^, and indication of the LGM and HS2 chronology^68,69^. B: Sea Surface Temperature reconstructions of marine drilling core MD95-2043 (Alborán Sea)^99^, and Heinrich Events detected in the same core. C: Percentage of temperate forest pollen in core MD95-2043^100^. D: Sea Surface Temperature reconstructions of marine cores MD95-2042 and SU81-18 (Atlantic)^101^ and Heinrich Events detected in the same cores. E: Percentage of temperate forest pollen in cores MD95-2042 and SU81-18. F: Main loess deposition periods and their sedimentation rates recorded in the Upper Tagus Basin (ochre bars)^40^ and Maximum extension stages of glaciers recorded in the Iberian Central System Range (blue bars)^102^. G: Estimated duration of the human occupation recorded at Peña Capón, based on the Bayesian Model 2 (Fig. 6) as calculated by the ‘date’ command in Oxcal.

The Peña Capón site hosts the oldest Upper Paleolithic presence recorded so far in central Iberia, starting at 26.1 ka cal BP as dated in level 6. This presence, currently accounting for the first peopling of the deep interior of the peninsula by modern humans, occurred during the central moments of HS 2, and hence during a cold and arid global period^68^. This harsh climate/environment is associated with the pre-Solutrean occupations of Peña Capón (levels 4, 5 and 6), dated between 26.1 and 25.0 ka cal BP. At these levels, data point to the presence of pine forests at higher altitudes and evergreen oak and juniper woodlands at lower ones, as well as to shrub and herb communities dominated by cryoxerophytic and heliophilous/cryophilous elements (LPAZ-1). Faunal associations, dominated by horse, show a generalized decrease in biodiversity as compared to levels 2a, 2b and 3, and include the presence of the cold-adapted *Microtus agrestis.* This paleoecological framework is consistent with the general context of increasing aridity and decrease in vegetation cover for the HS 2 in Iberia, as recorded both in continental^97,98^ and coastal archives^99–101^ (Fig. 11). Furthermore, it poses a marked contrast with the warm/humid conditions recorded in the Solutrean levels, except for level 1, which shows again a cold/arid landscape (LPAZ-3) between 24.1 and 23.8 ka cal BP.

Additionally, regional climatic and environmental proxies documented in inland Iberia point to harsh conditions during the time in which Peña Capón was occupied by hunter-gatherers. A pronounced period of loess deposition recorded in the Upper Tagus basin between 25.9 ± 2.4 ka and 23.2 ± 1.6 ka BP, interpreted as evidence of arid conditions^40,41^, matches the time span of the human presence at Peña Capón (Fig. 11). Also matching the first occupation of the site by humans, the maximum extent of glaciers recorded in the Iberian Central System range, dated at 26.1 ± 1.3 ka BP and backed by a local speleothem record, points to high precipitation rates within a cold period^102,103^ (Fig. 11). Further south, pollen data from the Fuentillejo maar lacustrine record and the TD core at *Tablas de Daimiel* National Park point to a cold period at ∼25–23 ka BP, characterized by Juniperus and xeric vegetation dominance^104,105^. All these data demonstrate that the first human settlement of this area of central Iberia occurred during a cold and mostly arid period.

These results confront traditional views and recent models positing that harsh climatic and environmental conditions hampered the human occupation of inland Iberia during most of the Upper Paleolithic. Geoarchaeological and paleoecological evidence gathered at Peña Capón demonstrate that this area of the Iberian hinterland was recurrently occupied during both temperate/humid and cold/arid periods, and thus regardless of climatic and environmental variability. Furthermore, although no other Solutrean or pre-Solutrean sites have been clearly identified thus far in the surrounding area of the site, the nearby caves of El Reno, El Cojo (both 9 km away), and Los Casares (76 km away) (Fig. 1D and Supplementary Fig. S1), were probably occupied by humans at the same time as Peña Capón. These sites host pre-Magdalenian rock art depictions, including cold-adapted fauna, which have been related to Solutrean or Gravettian times based on stylistic grounds and superimpositions with other images^43,106^. This, together with the nearby (but still poorly dated) Solutrean cluster of the Madrid basin^107^ strongly suggests that the human occupation sequence recorded at Peña Capón was not the product of isolated occasional visits to this region. Rather, it is increasingly clear that it was part of an organized settlement, established perhaps throughout the Tagus basin, and covering a prolonged sequence of time during the LGM, which in Peña Capón started at least in the HS 2 (Fig. 1C,D). Results of lithic raw material sourcing showing mobility between some Gravettian sites of the Côa valley (inner northeastern Portugal) and inner areas of the Northern and Southern *Mesetas*^23,108,109^, reinforces this hypothesis.

However, besides Peña Capón, pre-Solutrean occupations in inland Iberia remain absent for the central area of the peninsula, and they are very sparse in the rest of the plateau. Only at the western limit of the Northern *Meseta* there is sound evidence of Upper Paleolithic prior to 26 ka cal BP. Here, several Gravettian sites at the Côa valley are dated around 28 ka cal BP^23^, and the single site of Cardina-Salto do Boi have recently yielded an OSL date as old as 33.6 ± 2.0 ka cal BP associated to Aurignacian assemblages^14^ (Fig. 1B,C). Although these dates shorten the gap of human occupation after the Neandertal disappearance for the western margins of the plateau, in the rest of the Iberian hinterland, and especially in the very center, there is still a hiatus of ∼16,000 years devoid of human populations between ∼42 and 26 ka cal BP^40,110,111^.

## Conclusions

Given the current archaeological record, the argument that climate and environmental variability hampered the settlement of the Iberian hinterland by modern humans remains a valid hypothesis for the first phases of the Initial Upper Paleolithic in most of the inland territories. Yet, the fact that the first modern human presence recorded thus far in the deep interior occurred precisely during a cold and arid period of Heinrich Stadial 2, allows us to keep working on the hypothesis that the first settlement of these regions occurred earlier and regardless of climatic and environmental variability. As systematic fieldwork campaigns are still few in inland Iberia, ongoing and future research is very much needed to keep testing this and other competing hypotheses. Anyhow, the results presented in this paper show that the inland Iberian highlands were not an exception to the wide variety of landscapes to which Paleolithic hunter-gatherers were capable of adapting. Although it is well documented that large regions of Europe remained depopulated during long periods of the Last Glacial, it has also been thoroughly demonstrated that, given a certain availability of herbivore and plant resources, humans were always willing to expand and prosper anywhere, worldwide^112–117^.

## Methods

Methods for excavation, spatial recording and sampling are presented in Supplementary Text S2. Methods concerning geomorphology, sedimentology and micromorphology are provided in Supplementary Text S4.

### Radiocarbon dating and Bayesian modeling

In CologneAMS, bone samples were processed by collagen extraction and charcoals were AAA (Acid–Alkali–Acid extraction) processed according to sample preparation described by Rethemeyer et al.^118^. At ORAU, extraction, purification, and dating of bone collagen were carried out following ultrafiltration methods^119^, while dating of charcoal was undertaken using an ABOx-SC pretreatment^120^.

For Bayesian modeling we used the OxCal 4.4 online software^121^ and the most recent terrestrial radiocarbon curve, IntCal20^122^, to combine the radiocarbon likelihoods with the stratigraphic position of all samples. Since each sample was three-dimensionally recorded during excavation, relationships between samples and levels (including depth within a given level) were included within the Bayesian model as prior information. We used a General t-type Outlier Model^123^ with a resolution of 20 years and assigned 5% chances for each determination to be an outlier, as it is commonplace in recent research. In OxCal, commands and parameters are written in a C++ CQL (Command Query Language)^121^, and Model CQL codes are provided in Supplementary Text S6. The commands used to constrain the dated events in chronological order, group them within a given stratigraphic level, and calculate a start and end boundary (Probability Distribution Functions or PDFs) to bracket each archaeological episode, have been ‘*sequence’*, ‘*phase’* and ‘*boundary’* respectively^121^. Furthermore, we used the ‘*date’* command to query further on the accuracy of the time spans of each archaeological layer and the whole sequence of human occupation at Peña Capón.

In order to best identify outliers in the sequence, we run the model in two stages following the agreement index method as described by Bronk Ramsey^123^. Thus, after running a Preliminary Model (1) including all obtained radiocarbon measurements, a Final Model (2) was constructed by removing results with less than 60% agreement (matching those with posterior probabilities of > 5%), interpreted as potential outliers. Both models were run 3 times at > 3 million iterations and yielded no significant variation in their posterior results, thus showing that they were reproducible and the convergence values were high.

### Palynological analysis

During the 2015 season, eight sediment samples of 5 square cm were extracted for pollen analysis from the southern profile of square 2B (levels 1top, 1base, 2a, 2b, 4, 5top, 5base and 6) (Supplementary Fig. S23) and one more from the western profile of the same square (level 3) (Supplementary Fig. S20). Extraction followed standardized techniques for archeological sites^124,125^. The nine collected samples were prepared for pollen analysis (10 g per sample) at the CSIC labs (Madrid) following standard methods in archaeopalynology^124^, using treatment with HCl, 10% KOH, HF and concentration with Thoulet liquor, although acetolysis was not carried out to allow the identification of any contamination by modern pollen. The final residue was suspended in glycerin and counted until a pollen sum of 250 pollen grains was reached. Counting was undertaken using a Nikon Elipse 50i light microscope at × 400 magnification. Pollen grains were identified according to Moore et al.^126^ and Reille^127^ at the lowest currently possible taxonomical level. *Pinus nigra*-type pollen grains were categorized following measurements in Desprat et al.^128^. Pollen percentages were calculated using a pollen sum excluding indeterminable pollen grains (i.e., those that were broken, concealed, corroded, crumpled or degraded), as well as Asterioideae, Carduoideae and Cichorioideae with possible zoophily^125^, and presented as bars in a pollen diagram (Fig. 7). To establish the zonation of the pollen sequence, we tested several divisive and agglomerative methods with the program IBM SPSS Statistics 21. Based on the ecological meaning of the obtained zones, three local pollen assemblage zones (LPAZ-1 to LPAZ-3) were constructed on the basis of agglomerative constrained cluster analysis of incremental sum of squares (Coniss) with square root transformed percentage data^129^. The number of statistically significant zones was determined by using the broken-stick model^130^. Tilia and TGView^131,132^ and CorelDraw software were used to plot the pollen diagram.

### Anthracological analysis

Charcoal remains were sampled by hand during fieldwork and by flotation in the laboratories of the University of Alcalá and the Museo Nacional de Ciencias Naturales (Madrid). 131 samples from levels 1 to 6 have been studied in the Archaeobotanical laboratory of the Autonomous University of Barcelona and the Environmental Archeology laboratory of the CSIC (Madrid). A total of 197 fragments of carbonized wood were localized, of which 145 were identified to taxon. Identification of taxa was carried out following standard procedures. The anatomical patterns of each wood species were observed along three sections (transversal, longitudinal tangential, and longitudinal radial) using a reflected light microscope equipped with light field/dark field and objectives of 50 ×, 100 ×, 200 × and 500 ×. Archeological samples have been compared with modern woods as well as with wood anatomy atlases^133–135^.

### Microvertebrate analysis

In order to collect all small bone and teeth fragments from the fossil assemblages, all excavated sediments, bagged by sector, layer and stratigraphic level, were water-screened using superimposed 1.5 mm, 1.0 mm, and 0.5 mm-mesh screens, both at the site and in the labs of the University of Alcalá and the Spanish National Natural Sciences Museum (Madrid). A total of 226 bags (∼3–1 kg each) were wet-screened and the resulting concentrates were examined by naked eye as well as by optical microscopes. Microfauna and other small fragments of large fossils were separated by picking up the elements. The resulted collections of fossils were then sent to the Department of Earth Sciences of the University of Zaragoza, where assemblages were examined, photographed and stored. Additional washing with micro-mesh techniques and 10% HCl, and/or H2O2 was used when the surfaces of the molars, especially the enamel-dentine junction on the occlusal surface, were covered with particles of sediment that impeded the visual analysis. This anatomical region is needed pristine for the good classification and the morphometric analysis of small mammals. Drawings were made after photographs taken with an Olympus SZ61 microscope with a camera attached to it. Images and measurements were taken with the camera and the LCMicro software provided for the Olympus equipment.

Classification of small mammals into species was based on the morphology and biometry of the occlusal surface of the molars, following general criteria of systematic paleontology^136–138^. In each sample, we counted the number of skeletal elements, mainly dentition, and calculated the minimum number of Identified species (NISP). Interpretation of the microvertebrate assemblages in palaeoenvironmental terms is based on the taxonomic association present in each archaeological layer and their ecological preferences, as well as their temperature and humidity limitations as a whole assemblage.

### Zooarcheology

17,275 faunal remains from levels 1 to 6 of Peña Capón, coming mostly from the 2015 season, were subject to zooarcheological and taphonomic analyses. Studied remains included both identifiable and unidentifiable fragments and their taxonomic identification were based on reference material held at the Prehistory Department of the Complutense University of Madrid (Spain). When the identification was not feasible, epiphyses, axial and shaft fragments were assigned to three animal weight/size classes: 1) small-sized carcasses, < 100 kg (e.g. *Capra pyrenaica, Rupicapra rupicapra*), 2) medium-sized carcasses, > 100–300 kg (e.g. *Cervus elaphus*) and 3) large-sized carcasses, > 300 kg (e.g. *Equus ferus, Bos primigenius*) [see^110^].

The estimation of NISP (Number of Identified Specimens) and MNI (Minimum Number of Individuals) were used to quantify the faunal remains and determine the most appropriate features of the faunal taxonomic distribution. NISP determination follows Lyman^139^, whereas MNI is based on Brain’s^140^ method, which uses bone laterality and estimated age. Furthermore, skeletal profiles and MNI consider shaft thickness, section shape and medullar surface properties^141^. In this way, bones were divided into four anatomical regions: 1) cranial (antlers-horn, skull, mandible and dentition), 2) axial (vertebrae, ribs, pelvis and scapula, sensu^142^), 3) upper appendicular limbs (humerus, radius, ulna, femur, patella and tibia) and 4) lower appendicular limbs (metapodial, carpals, tarsals, phalanges and sesamoideal).

### Lithic analysis

After manual cleaning and removal of adhering concretion (Supplementary Fig. S17), lithic artifacts were studied at the Prehistory Laboratory of the University of Alcalá. We followed the *chaîne opératoir*e or ‘operational sequence’ approach [e.g.^143,144^], combined with the ‘Technological organization’ approach aimed at examining links between technology and paleoenvironmental change [e.g.^145–147^]. We assigned each lithic artifact to one of the three *chaîne opératoire* stages commonly recognized in the literature (Supplementary Dataset 5). Thus, cortical flakes, preparation products and tested cores were assigned to the initialization stage or phase I; raw blanks, core maintenance by-products, thinning flakes and productive cores to the exploitation stage or phase II; and retouched blanks, retouching flakes and exhausted cores to the consumption and abandonment stage or phase III. The study of bifacial reduction sequences aimed at the production of foliate armatures followed methods described in Alcaraz-Castaño et al.^107^ and were ultimately backed in experimental flintknapping works^148–150^.

## Supplementary Information

Supplementary Dataset 1.

Supplementary Dataset 2.

Supplementary Dataset 3.

Supplementary Dataset 4.

Supplementary Dataset 5.

Supplementary Information 6.

Supplementary Video 1.

## Data Availability

The authors declare that all data supporting this research are available within the paper, its Supplementary Information and Supplementary Data files. The Peña Capón archaeological assemblages are housed in the History and Philosophy Department (Prehistory Area) of the University of Alcalá and the *Museo de Guadalajara* (Guadalajara, Spain). Both repositories are accessible for all researchers upon request.
