# Supplementary material for: First modern human settlement recorded in the Iberian hinterland occurred during Heinrich Stadial 2 within harsh environmental conditions
Source: Sci Rep. 2021 Jul 26;11:15161. doi: 10.1038/s41598-021-94408-w (PMC8313528; doi:10.1038/s41598-021-94408-w)
Supplement: Supplementary file 6 — Supplementary Information 6. [file 41598_2021_94408_MOESM6_ESM.pdf]

First modern human settlement recorded in the Iberian hinterland occurred during Heinrich Stadial 2 within harsh environmental conditions

M. Alcaraz-Castaño [(1,)], J. J. Alcolea-González [(1)], M. de Andrés-Herrero [(1)], S. Castillo-Jiménez [(1)], G. Cuenca-Bescós [(2)], F. Cuartero [(3)], M. Kehl [(4)], J. A. López-Sáez [(5)], L. Luque [(1)], S. Pérez-Díaz [(6)], R. Piqué [(7)], M. Ruiz-Alonso [(5)], G.-C. Weniger [(8)], J. Yravedra [(9)]*

(1) Prehistory Area, University of Alcalá (Spain). *manuel.alcaraz@uah.es
(2) Aragosaurus-IUCA, Department of Geosciences, University of Zaragoza (Spain).
(3) Atapuerca Foundation (Spain)
(4) Institute of Geography, University of Cologne (Germany).
(5) Environmental Archeology Research Group, Institute of History, CCHS CSIC (Spain).
(6) Department of Geography, Urban and Regional Planning. University of Cantabria (Spain).
(7) Department of Prehistory, Autonomous University of Barcelona (Spain).
(8) Neanderthal Museum (Germany).
(9) Department of Prehistory, Complutense University of Madrid (Spain).

Supplementary information

Content

SI.1 – History of research of the Peña Capón site 3

Text S1. History of research on the Peña Capón archaeological site 3

Figure S1. Location of the study area in the Iberian Peninsula (A), the Guadalajara province (B) and the upper stretches of the Sorbe and Jarama River valleys (C) 4

Figure S2. General views of the Peña Capón rock shelter 5

Figure S3. Lithic assemblages from levels II and III of Peña Capón as defined in the 1972 excavation 6

Table S1. Sampled materials and radiocarbon dates from the Peña Capón sequence as defined in the 1972 excavation 7

S1.2 – Fieldwork at Peña Capón during the 2015 and 2019 seasons 7

Text S2. Field methods: excavation, spatial recording and sampling 7

Figure S4. Progressive lowering of the water level at the Beleña reservoir from spring to autumn, in 2015 8

Figure S5. Views and plan of the Peña Capón archaeological deposit 9

Figure S6. Views of The Peña Capón rock shelter and surroundings showing the verticalized dolostone layers that likely protected the deposit from reworking and erosion 10

Figure S7. Landscapes surrounding the Peña Capón rock shelter 11

Figure S8. Excavation and recording methods 12

Figure S9. Excavation process at the top of the sequence (level 1) and the base of level 3 13

Figure S10. Plan of level 1 showing recorded archaeological items > 2cm 14

Figure S11. (A) Excavation area during the excavation of level 2a (layer 1). (B) Excavation area during the excavation of level 2b (layer 10) 15

Figure S12. Plan of level 2a showing recorded archaeological items > 2cm 16

Figure S13. (A) Fireplace recorded in square 2B of level 2a (layer 3). (B) Detail. (C) Excavation process of the fireplace 17

Figure S14. (A) Excavation area during the excavation of level 2b (layer 6; end of the 2015 season). (B) Excavation area during the excavation of level 3 (layer 2; end of the 2019 season) 18

Figure S15. Plan of level 2b showing recorded archaeological items > 2cm 19

Figure S16. Orthomosaic showing the base (layer 12) of Level 2b 20

Figure S17. Preform of foliate point in an advanced stage of reduction from level 2b 21

Figure S18. 3D Model of the excavation area at the base (layer 12) of Level 2b 22

S1.3 – The modern human settlement of the Iberian Peninsula 22

Text S3. Criteria for considering radiometric dates and archaeological sites as valid markers for human presence at a given time 22

SI.4 – Gemorphology, sedimentology and micromorphology 23

Text S4. Methods: geomorphology, sedimentology and micromorphology 23

Text S5. Detailed sedimentological and micromorphological description of the archaeological deposit 24

Figure S19. Sediment properties of the western profile of square 2B 27

Table S2. Micromorphological description of thin sections from different levels at Peña Capón 28

Figure S20 Western profile of square 2B showing sediment blocks reinforced with gypsum bandages prior to their extraction for micromorphological study 29

SI.5 – Radiocarbon dating and Bayesian modeling 30

Table S3. Sampled materials and radiocarbon dates from the Peña Capón sequence 30

Table S4. Detailed results of Bayesian Model 1 (Preliminary model) 32

Figure S21. Bayesian Preliminary Model (1) for the Peña Capón sequence 33

Table S5. Detailed results of Bayesian Model 2 (Final model) 35
Figure S22. PDFs for the estimated duration of all archaeological levels at Peña Capón, based on the Bayesian Model 2 as calculated by the 'date' command 36
Text S6. CQL Codes for Bayesian analyses 37

SI.6 – Pollen 43
Figure S23. Stratigraphic sequence recorded in the southern profile of square 2B showing sample location for pollen analysis 43

SI.7 – Microvertebrates 44
Figure S24. Distribution of small vertebrates (NISP) throughout the Peña Capón sequence 44
Figure S25. Relation between collected sediment samples and identified taxa of small vertebrates at Peña Capón 44

SI.8 – Macrovertebrates 45
Text S7. Mortality patterns and taphonomic analysis 45
Table S6. Mortality patterns among macrovertebrates at the Peña Capón sequence 45
Table S7. Bone surface modifications recorded at the Peña Capón macrovertebrates assemblages 46
Figure S26. Examples of cut-marked shafts from level 1, level 2a and level 3 47
Table S8. Frequencies of cut marks recorded at the Peña Capón macrovertebrates assemblages 47
Table S9. Frequencies of percussion marks recorded at the Peña Capón macrovertebrates assemblages 48

Acknowledgements 48

Supplementary references 49

SI.1 – History of research and excavation of the Peña Capón site

Text S1. History of research on the Peña Capón rock shelter

Peña Capón was discovered in 1970 and excavated in 1972 during two field seasons. Fieldwork was conducted by a team led by J. Villasenín-Gómez and directed by J. Martínez Santa-Olalla (Alcaraz-Castaño et al. 2013, 2019; Alcaína et al. 2018). However, results of these excavations were never published, and it was only in the late 1990s that the archaeological assemblages, diaries, and photos from the field seasons were gathered and analyzed in a preliminary paper on the site by Alcolea-González et al. (1997). Since this first publication, in which a preliminary archeological sequence was presented, the relevance of this site was evident: in no other location on the whole Iberian plateau have Solutrean and pre-Solutrean assemblages been described within a multi-layered sequence (see Alcaraz-Castaño, 2015). However, those assemblages, including lithic and faunal remains, were recovered in a poorly-recorded excavation lacking any stratigraphic, chronometric or paleoenvironmental data, besides a few photographs and oral records (Alcolea-González et al., 1997). Therefore, the position that many researchers adopted where the relevance of Peña Capón was concerned, was one of caution until more robust evidence was presented (e.g. Delibes and Díez, 2006: 16, Mosquera et al., 2007: 151 and Cacho et al., 2010: 117).

The first attempt aimed at solving the methodological inconsistencies and the lack of sound data at Peña Capón, was carried out by Alcaraz-Castaño et al. (2013). Until then, an excavation of the site had not been possible due to the difficulties of accessing the site, which is often under water due to the construction of a

dam in 1982 (Figs. S1, S2, S4-S7). Thus, these scholars limited their work to an in-depth study of the lithic and faunal assemblages and the radiocarbon dating of a single tooth per level, as preliminarily defined according to the data of the 1972 fieldwork (Table S1). This confirmed that an occupation sequence around the time of the LGM (*sensu* Clark et al. 2009) was indeed present at Peña Capón, and that this included at least Upper Solutrean (Level I), Middle Solutrean (Level II) and Proto-Solutrean assemblages (Level III), as well as a potential Gravettian component (Level IV (Fig. S3) [note that in publications prior to Alcaraz-Castaño et al. (2019), levels from the 1972 excavation were numbered in Arabic numbers (1, 2, 3, 4) (see Alcaraz-Castaño, 2015: fig. 4). However, to make a clear distinction between them and levels from the recent excavations, levels from the 1972 fieldwork are now referred using Roman numbers (I, II, III, IV)]. While zooarcheological and taphonomic studies have been conducted for levels II and III, including stable isotope analyses on the teeth of herbivores (Alcaraz-Castaño et al., 2013; Yravedra et al., 2016), analyses of lithic assemblages have only been thoroughly published for the Proto-Solutrean level (III) (Alcaraz-Castaño et al., 2013; see Alcolea-González et al. 1997).

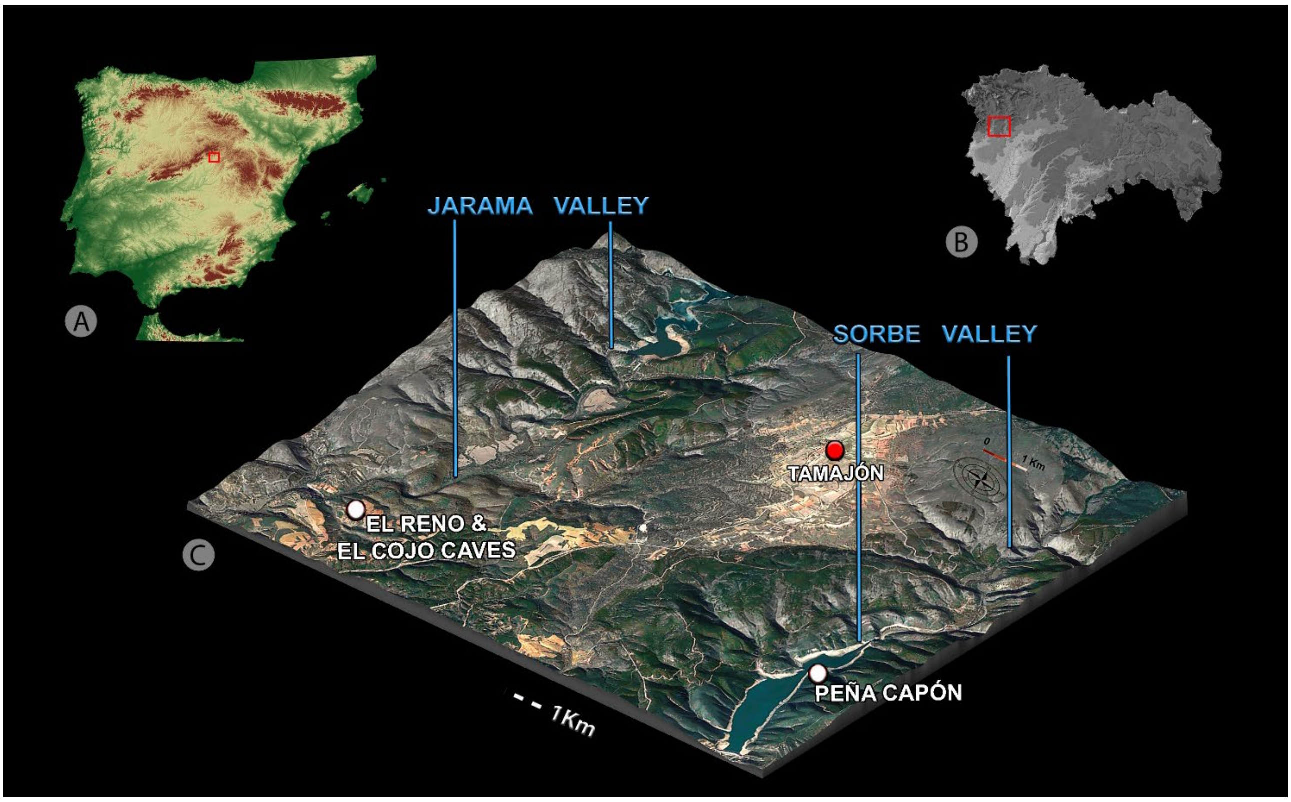

Figure S1. Location of the study area in the Iberian Peninsula (A), the Guadalajara province (B) and the upper stretches of the Sorbe and Jarama River valleys (C). Map A generated with ArcGIS (ArcMap 10.3.1.) (https://www.arcgis.com/index.html) using ASTER Global Digital Elevation Model V0032019, distributed by NASA EOSDIS Land Processes DAAC, (https://doi.org/10.5067/ASTER/ASTGTM.003). Maps B and C generated with 3D Map Generator –Atlas v.1.3 *(https://www.3d-map-generator.com/3d-map-generator-atlas/) and Adobe Photoshop v. 22.4.2 (https://www.adobe.com/es/products/photoshop.html).*

The archaeology of the Proto-Solutrean level (III), as defined by workers of the old excavation, is characterized by the presence of Vale Comprido points, which are made on relatively thick triangular blanks with dorsally thinned butts (Fig. S3). Together with a high presence of endscrapers and scaled pieces (*écailles*), other typical Proto-Solutrean features (Zilhão & Aubry 1995; Zilhão 1997; Renard 2011; Belmiro et al. 2020), such as the production of bladelets obtained from carinated cores and retouched marginally or left unretouched, are also found. However, given that excavation and study methods for the old fieldwork were most probably biased towards the largest products and the flint objects (Alcaraz-Castaño et al. 2013), a more fine-grained techno-typological characterization of this and the other assemblages defined in 1972 is not possible.

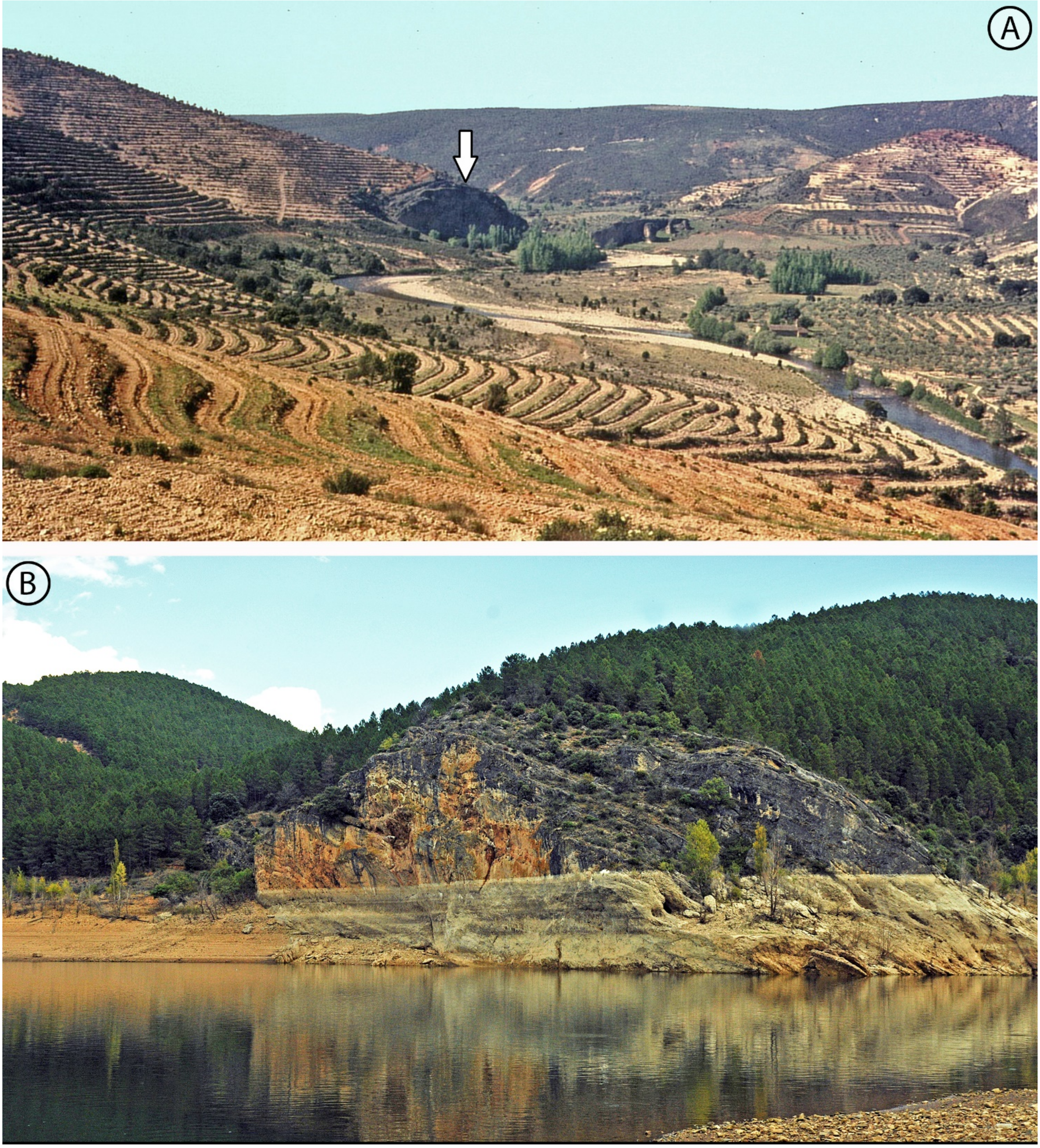

Figure S2. General views of the Peña Capón rock shelter (A) View from the northeast in 1980, before the construction of the dam. (B) View from the northwest in October 2012.

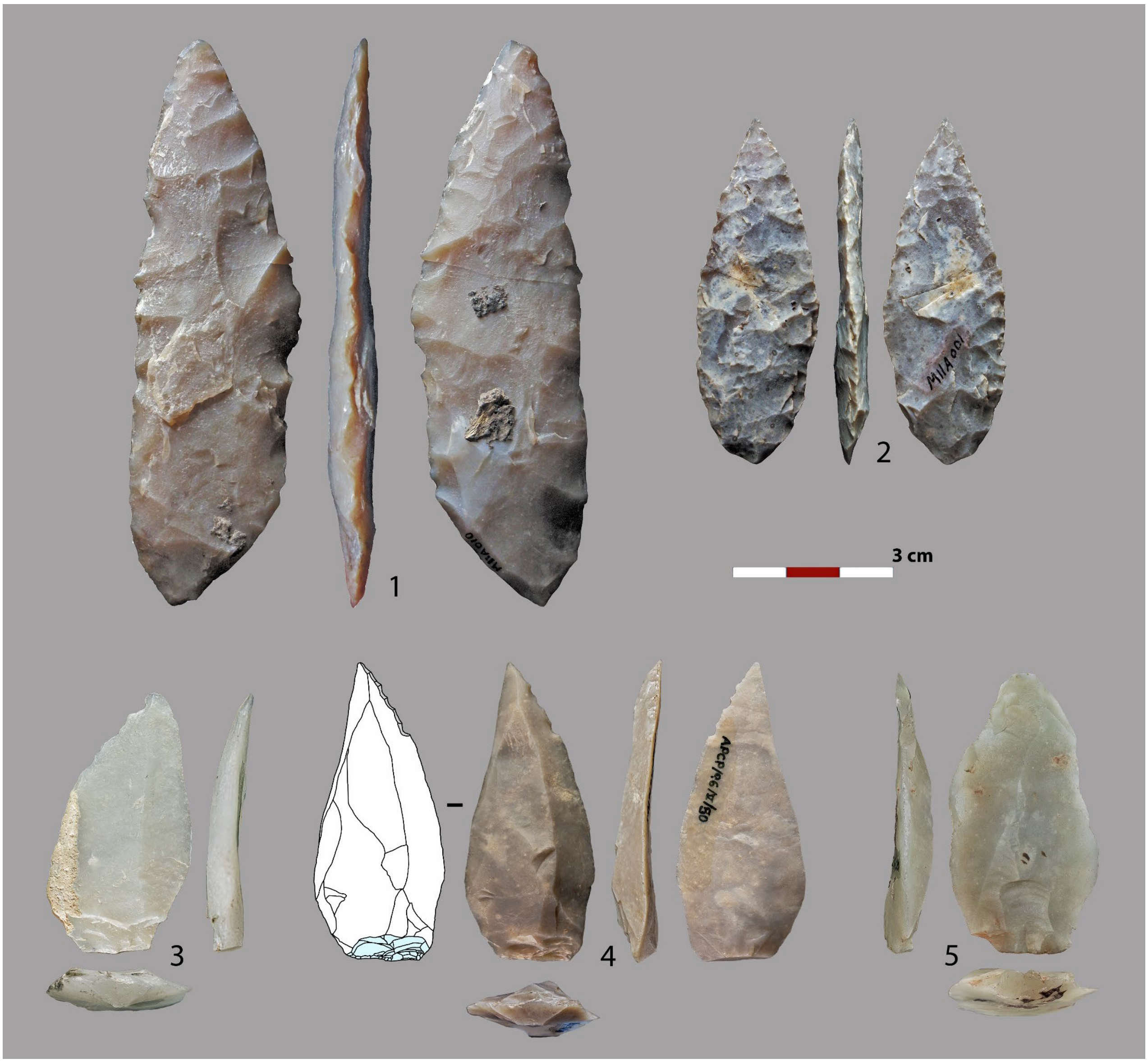

Figure S3. Lithic assemblages from levels II and III of Peña Capón as defined in the 1972 excavation*. 1-2: Laurel leaf points from the Middle Solutrean (level II). 3-5: Vale Comprido points from the Proto-Solutrean (level III).*

Although the radiocarbon dates obtained in 2013 at Beta Analytic for the 1972 assemblages (Alcaraz-Castaño et al. 2013) confirmed a LGM chronology for the whole sequence recorded at Peña Capón, the virtually identical results obtained for the Solutrean (Beta – 246880) and the Proto-Solutrean (Beta – 246879) levels posed a problem (Table S1). This incoherence between the stratigraphic sequence (as described by the 1972 excavators) and the radiocarbon age of these samples could be interpreted as a result of the potential mixing of materials, either within the site or after the excavation. The new radiocarbon measurements obtained at the CologneAMS laboratory favor the second hypothesis, since a significant older age has been obtained for a cut-marked bone assigned to the Proto-Solutrean level (Table S1). Thus, the reality of the archaeological sequence described in 1972 can be accepted in general terms, although a more precise stratigraphy has been recorded in the recent fieldworks (see main text). Anyhow, although the new radiocarbon determinations for the Solutrean (COL4223.1.1) and Proto-Solutrean (COL4224.1.1) levels of the 1972 excavation sufficiently mirror those obtained for the Solutrean (levels 1-3) and pre-Solutrean (levels 4-6) of the new excavations, we decided to keep both contexts aside, given the impossibility of establishing a secure correlation between stratigraphic units of both excavations.

Level	Sample ID & material	Lab-ID	C^{14} BP	δ13C (‰)	Age cal BP (95,4%)
II (Solutrean)	PCP Bone 16-5	COL4223.1.1	20204 ± 108	-15.2	24270 - 23812
	Tooth B-5	Beta - 246880	19930 ± 110	-20.2	24213 - 23780
III (Proto-Solutrean)	PCP Bone 16-6	COL4224.1.1	20863 ± 116	-15.3	25596 - 24853
	Tooth B-4	Beta - 246879	19980 ± 110	-20.4	24239 - 23800
IV (Gravettian?)	PCP Bone 16-7	COL4225.1.1	21647 ± 123	-17,8	26258 - 25726
	Tooth B-3	Beta - 246878	21220 ± 120	-20.5	25819 - 25251

Table S1. Sampled materials and radiocarbon dates from the Peña Capón sequence as defined in the 1972 excavation. All bone samples were cut-marked. C^{14} dates were calibrated with OxCal 4.4 (Bronk Ramsey 2009a) using IntCal20 (Reimer et al. 2020).

As for the purportedly Gravettian level (IV) as defined in 1972, a new radiocarbon measurement (COL4224.1.1) has also shown a slightly older result than the previously provided by Beta, which is also in consonance with new age determinations obtained both at CologneAMS and ORAU for the base of the sequence (see main text). Yet, the Gravettian nature of this level, which was based not only on its radiocarbon age and chrono-stratigraphic position, but also in the high presence of quartz in the recorded assemblage, remains as a hypothesis. Although relatively large amounts of quartz have been recorded for levels 4-6 of the new excavation (Supplementary Dataset 5), thus fulfilling one of the main features of the so-called Terminal Gravettian in Portugal (Almeida 2000; Belmiro et al. 2020), no other typological features or technological strategies supports a clear techno-cultural attribution for these levels. Thus, in the main text levels 4 to 6 are provisionally defined as pre-Solutrean, until a precise chrono-cultural attribution, either Gravettian or Proto-Solutrean, could be proposed for each of them.

S1.2 – Fieldwork at Peña Capón during the 2015 and 2019 seasons

Text S2. Field methods: excavation, spatial recording and sampling

As previously mentioned, the Peña Capón site is often underwater due to the construction of a dam in 1982, just 2.7 km downstream, close to the village of Beleña de Sorbe (Fig. S1). Thus, the site is currently well within the Beleña reservoir, and usually during most of the year is completely covered by water. However, depending on rainfalls the site is accessible during autumn and early winter in dry years (Fig. S4). This was the case in 2015 and 2019, when the only two campaigns carried out by our team to date, were developed at Peña Capón.

Fieldwork during the 2015 and 2019 seasons was based on the excavation of six square meters at a central location in the rock shelter (Fig. S5), potentially very close to the area excavated in 1972. Excavation of sediments followed standard methods in Paleolithic Archeology. Within stratigraphic units we used small excavation tools (trowels, dental tools, wooden sticks and brushes) for developing artificial layers of 3cm thick (Figs. S8, S13). Reworked areas, mostly corresponding to the 1972 excavation (Fig S9), were identified and removed until *in situ* sediment was found. In the last season (2019) we reached a maximum depth of ~65 cm in five of the squares, while in one of them (square 2B) we conducted a test pit up to a depth of ~95 cm. At this height, we reached a dolostone surface covering the whole area of the test pit (Fig. 4 in the main text and Figs S14-S16). Whether this dolostone surface accounts for the bedrock of the rock shelter or for a large block. is still to be confirmed in future field seasons.

Artificial layers, stratigraphic units, the grid, and every archeological object or feature larger than 2 cm (lithics, bones, charcoal fragments, ocher remains and human-made structures) were georeferenced and three-dimensionally recorded using a Leica TS02 Total Station and a Leica Topographic GPS (GS16/ICG70), and orientation and dip of elongated bone and lithic products were registered. Before individually bagging archaeological items, all excavation layers were digitally photographed with a Nikon D5100+Sigma 17-70 mm Macro f 2.8-4 (2015 season) and a Pentax K-1 II + Pentax FD24-70mm f2.8 (2019 season), and then recorded

using a Microsoft Surface touchscreen-based PC. 3D photogrammetric models and orthomosaic plans were produced using Agisoft Metashape Professional v.1.5.2. and Adobe Creative Cloud (Photoshop v. 22.4.2. and Bridge v.11.1). The spatial distribution of items was analyzed using ArcGIS (ArcMap 10.3.1.). Stratigraphic profiles and some excavation plans were also hand-drawn. Every square meter was divided into four sectors of 0,25 sq m and sediment and items <2 cm were packed accordingly. This sediment was later floated and wet-screened at 2-, 1- and 0,5 mm mesh sieves both in the field and the laboratories of the University of Alcalá and the *Museo Nacional de Ciencias Naturales (Madrid)*, where most of the lagomorphs, microvertebrates, charcoals and lithic debris were acquired. Samples for micromorphology and pollen analyses were also collected during excavation.

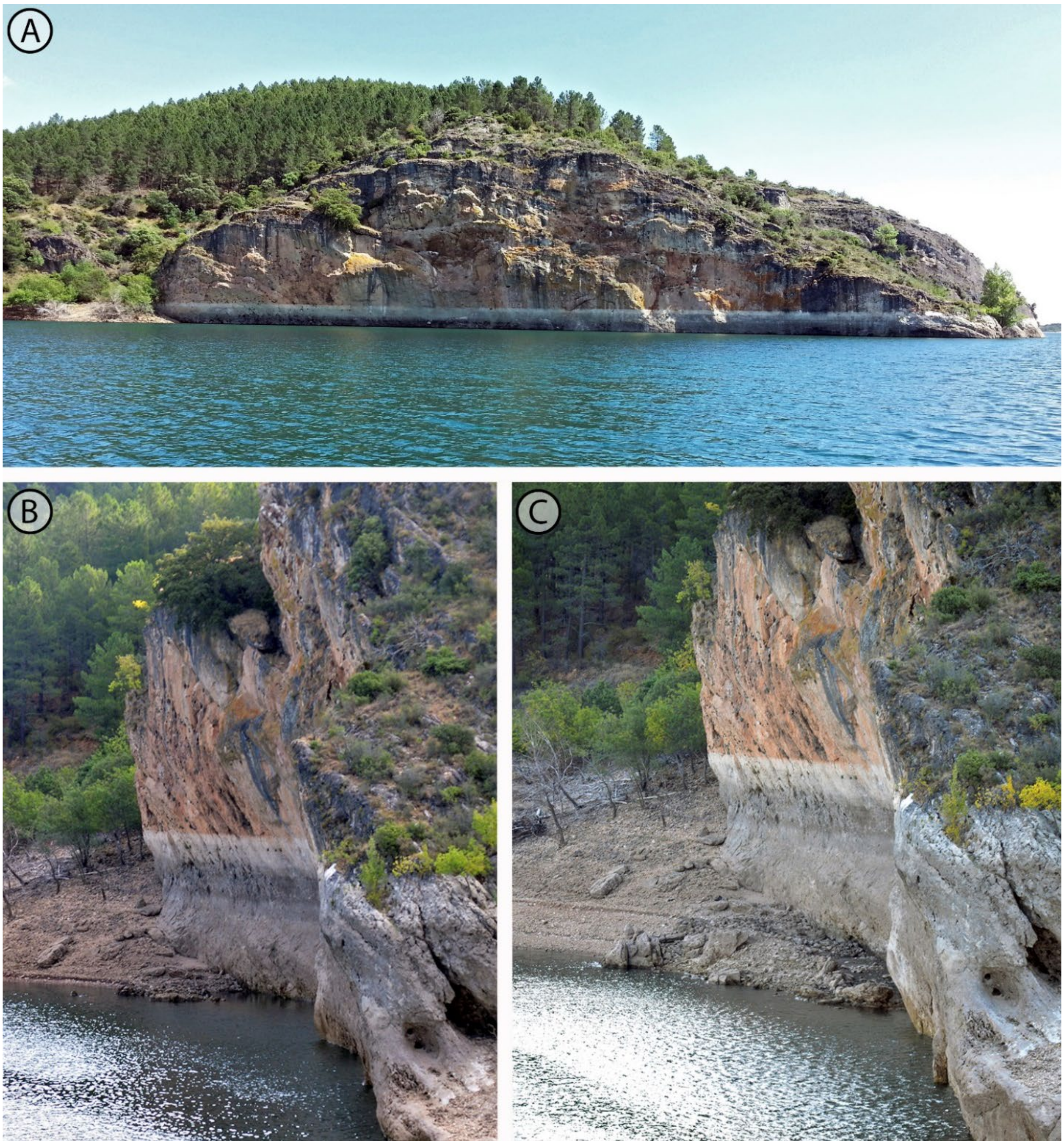

Figure S4. Progressive lowering of the water level at the Beleña reservoir from spring to autumn, in 2015. (A) June 4th, 2015: Peña Capón completely covered by the reservoir waters. (B) September 2nd, 2015: the highest area of the deposit emerges. (C) September 25th, 2015: part of the deposit is uncovered, and the site is nearly available for excavation.

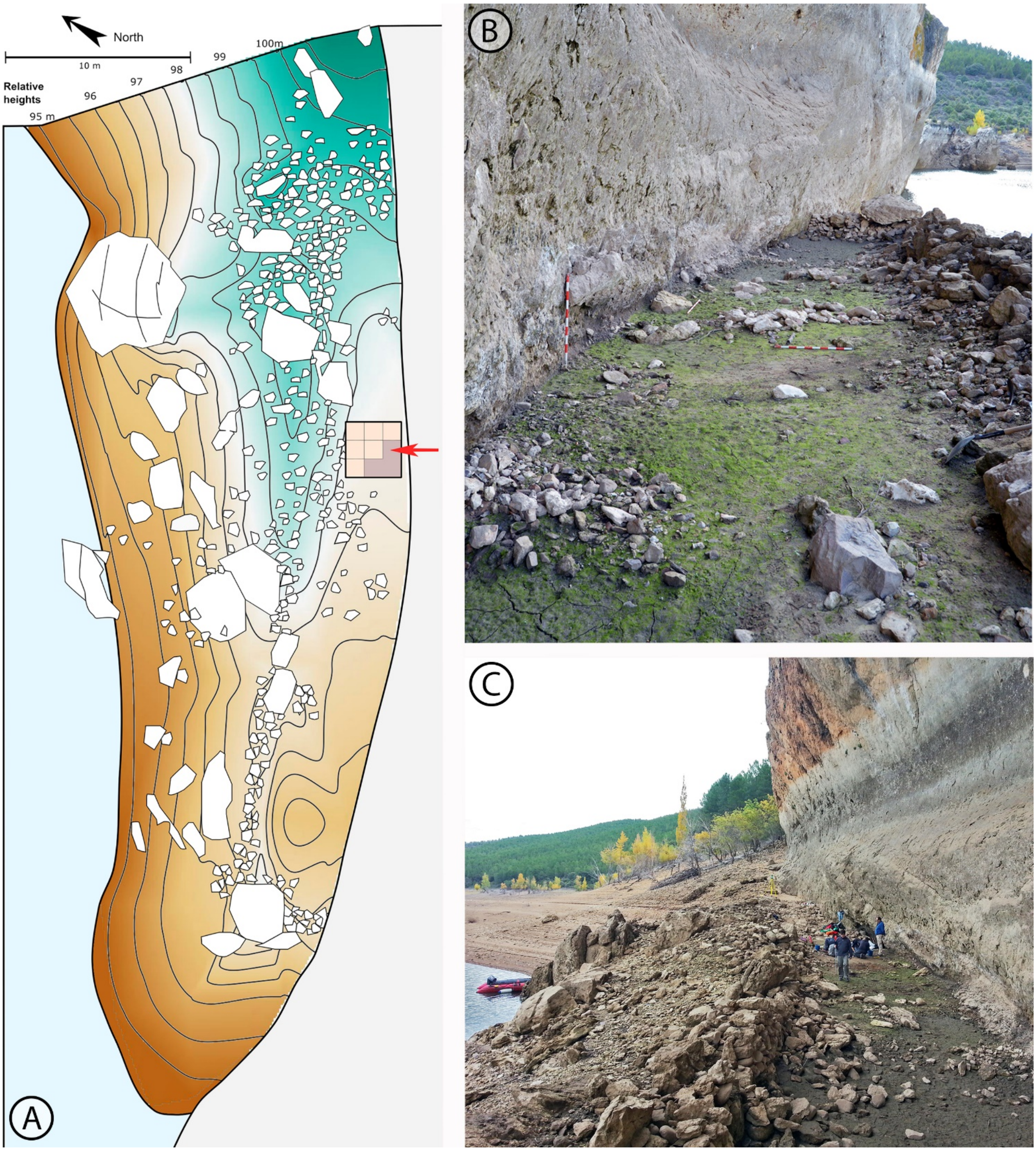

Figure S5. Views and plan of the Peña Capón archaeological deposit. (A) Topographic plan showing the excavation area (red arrow). (B) View from the east in 2015, before the excavation season. (C) View from the west.

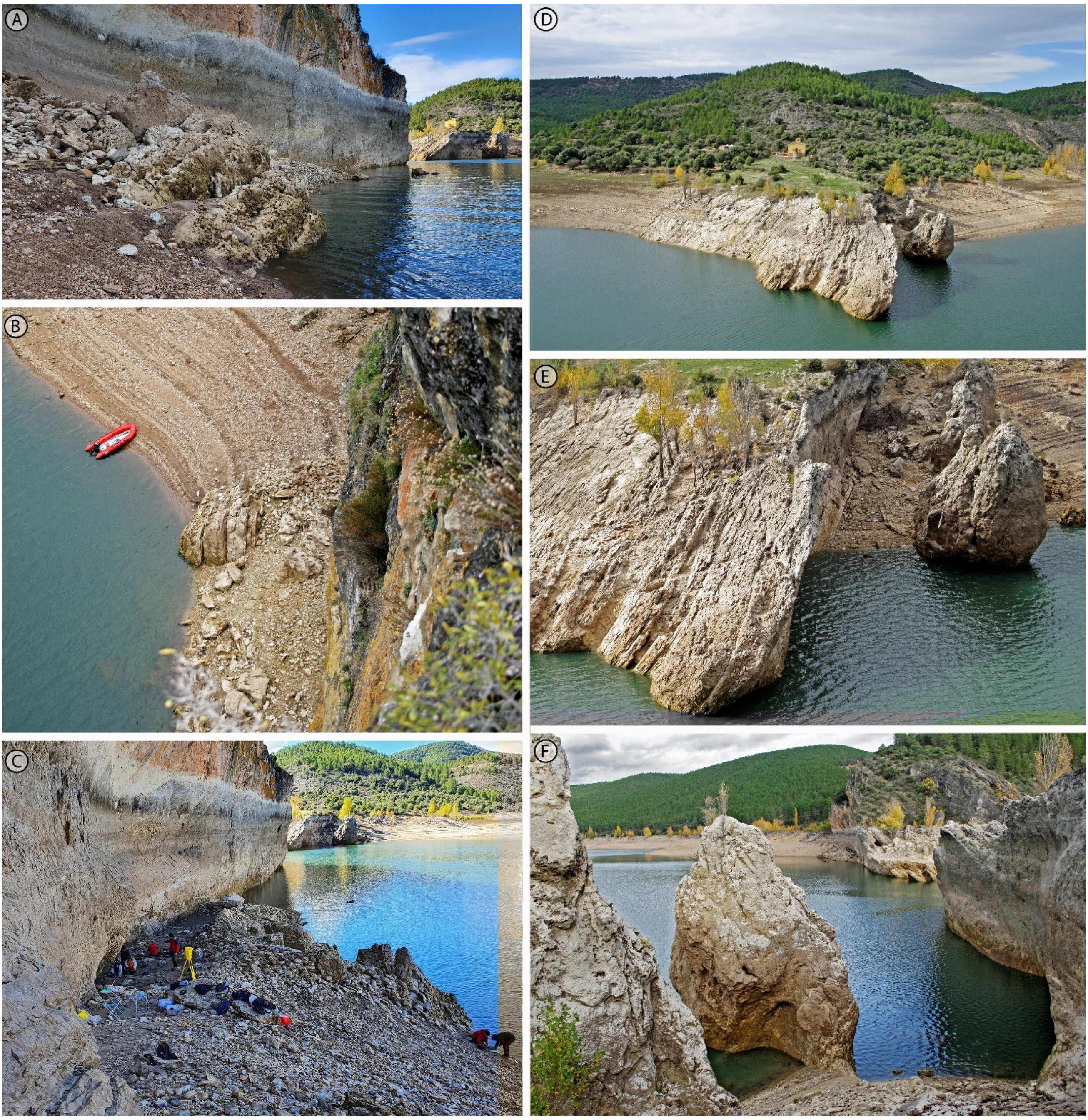

Figure S6. Views of The Peña Capón rock shelter and surroundings showing the verticalized dolostone layers that likely protected the deposit from reworking and erosion. (A) View from the north, at the foot of the site. (B) View from above the rock shelter. (C) View from the east. (D) Verticalized dolostone layers in the opposite bank, aligned with those at the foot of the archaeological deposit (see view A). (E) Detailed view of the same layers. (F) View of the rock shelter from the opposite bank.

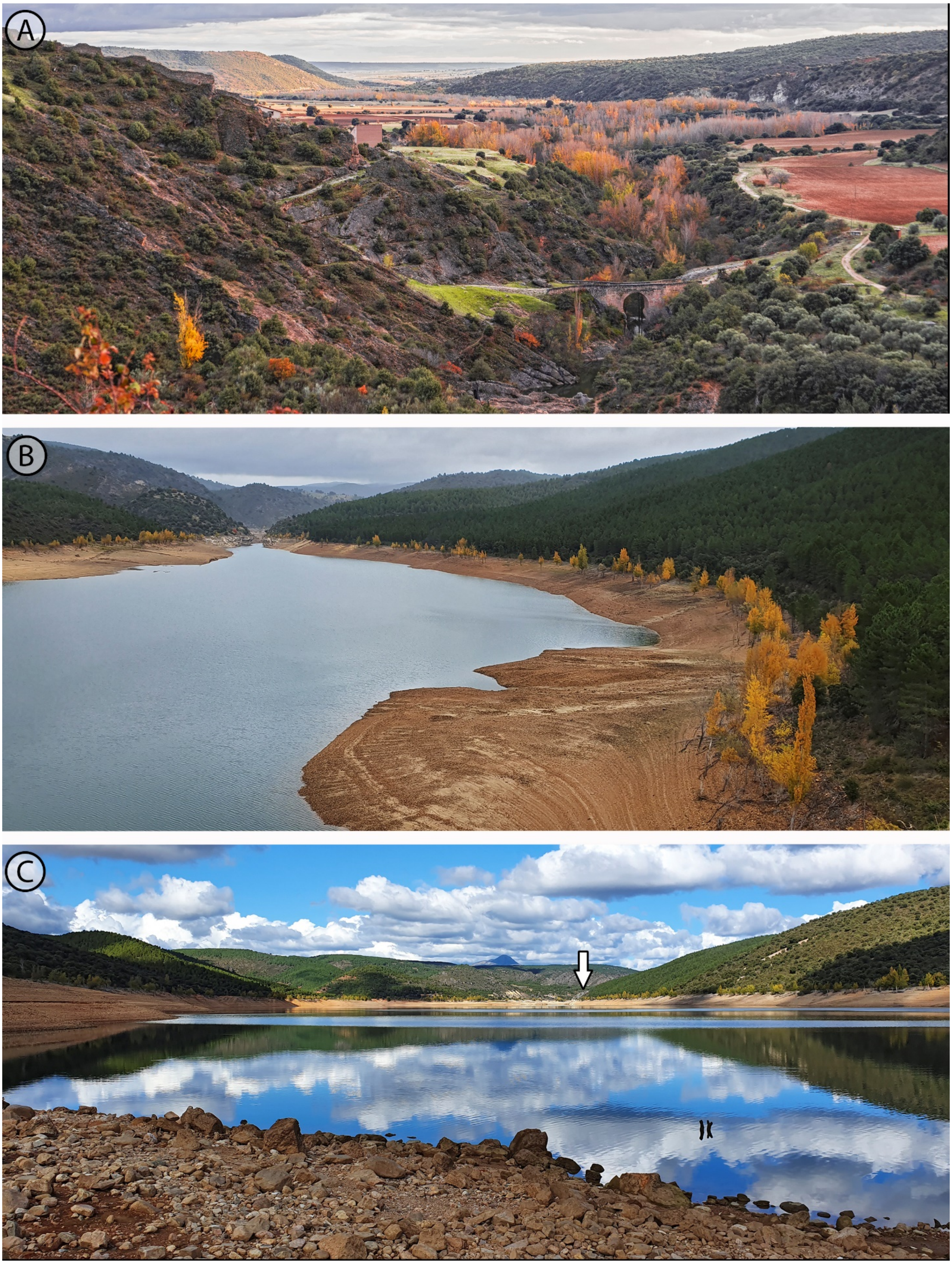

Figure S7. Landscapes surrounding the Peña Capón rock shelter. (A) View of the Sorbe River Valley from the Beleña dam, 2.7 km downstream from Peña Capón. View of the Sorbe Valley (as within the reservoir) to the north, from the top of the rock shelter. (C) View from the dam to the north, with the Ocejón peak (Ayllón Mountains, Iberian Central System Range) in the distance. The site is marked by a white arrow.

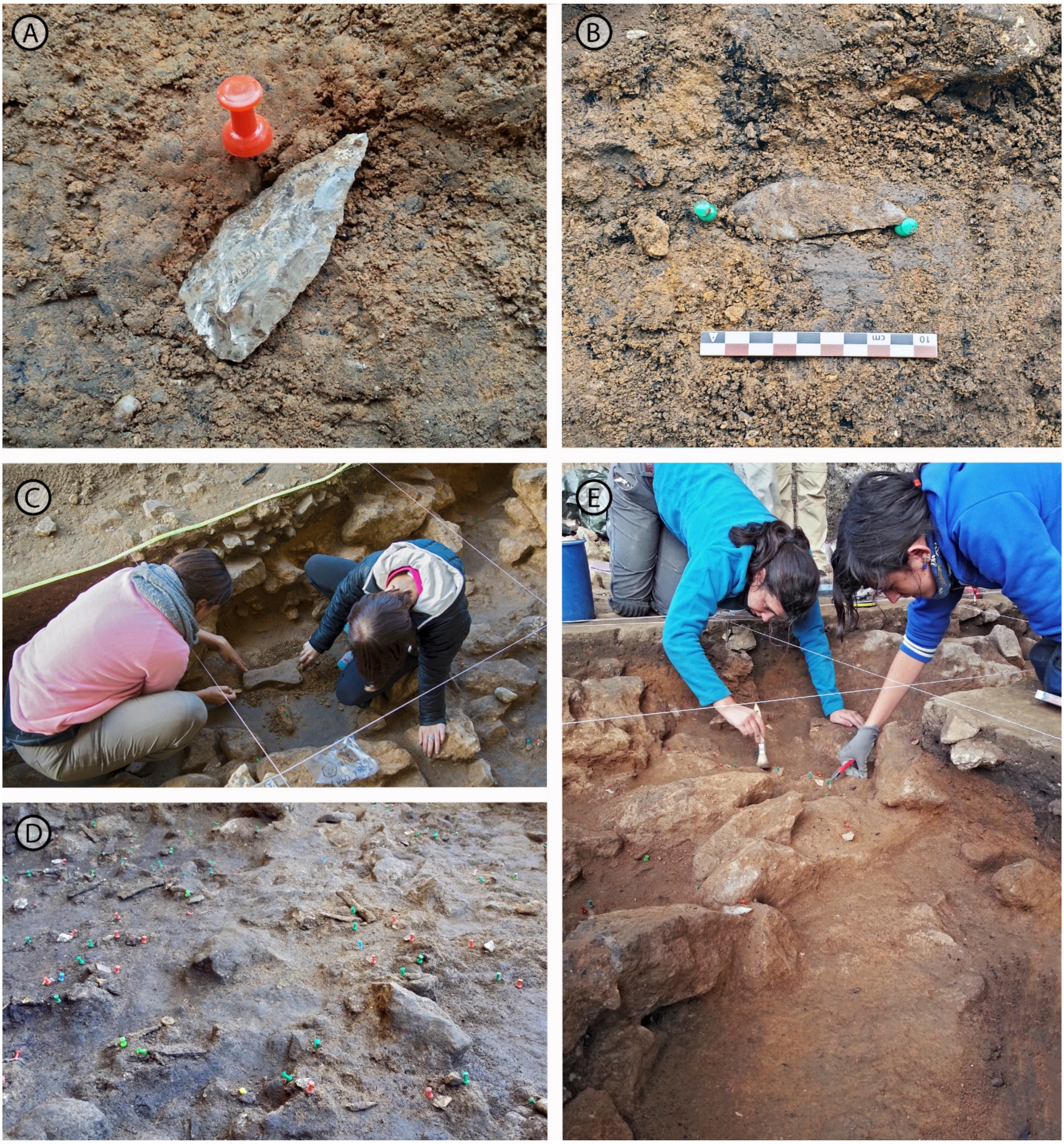

Figure S8. (A) Excavation and recording methods. (A) Rock crystal point recorded in level 1 after first cleaning (see Fig. 9.2 in the main text). (B) Foliate point recorded in level 2b (see Fig. 9.9 in the main text). (C) Excavation of a horse scapula from level 2a. (D) Close view of square 2B during the excavation of level 2b (layer 1). (E) Excavation of level 1 (layer 4)

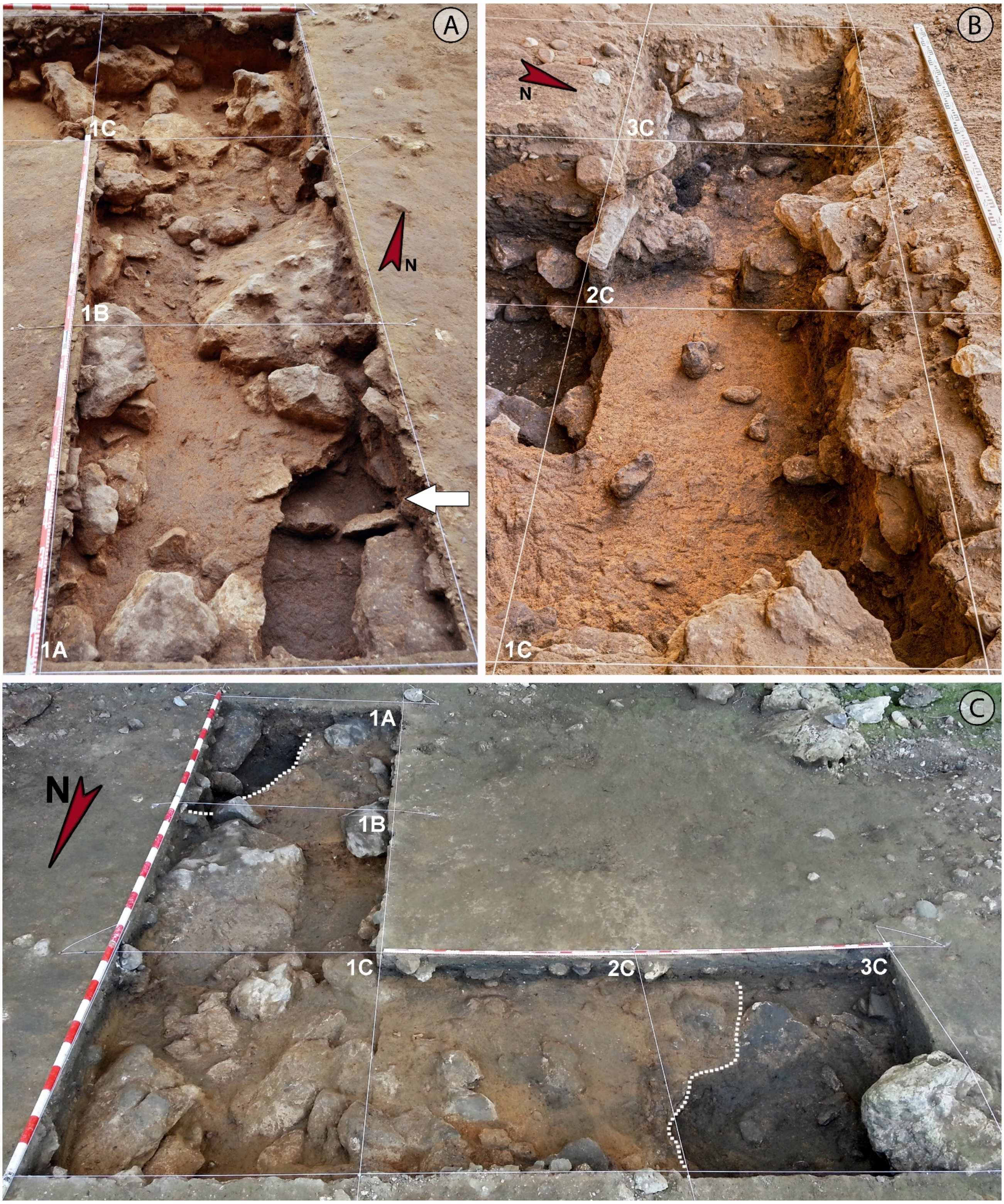

Figure S9. Excavation process at the top of the sequence (level 1) and the base of level 3. (A) Level 1 during excavation, showing a disturbed area corresponding to the 1972 excavation in square 1A. (B) Top of Level 3. (C) General view of the excavation area at the top of level 1, showing reworked areas in squares 1A and 3C.

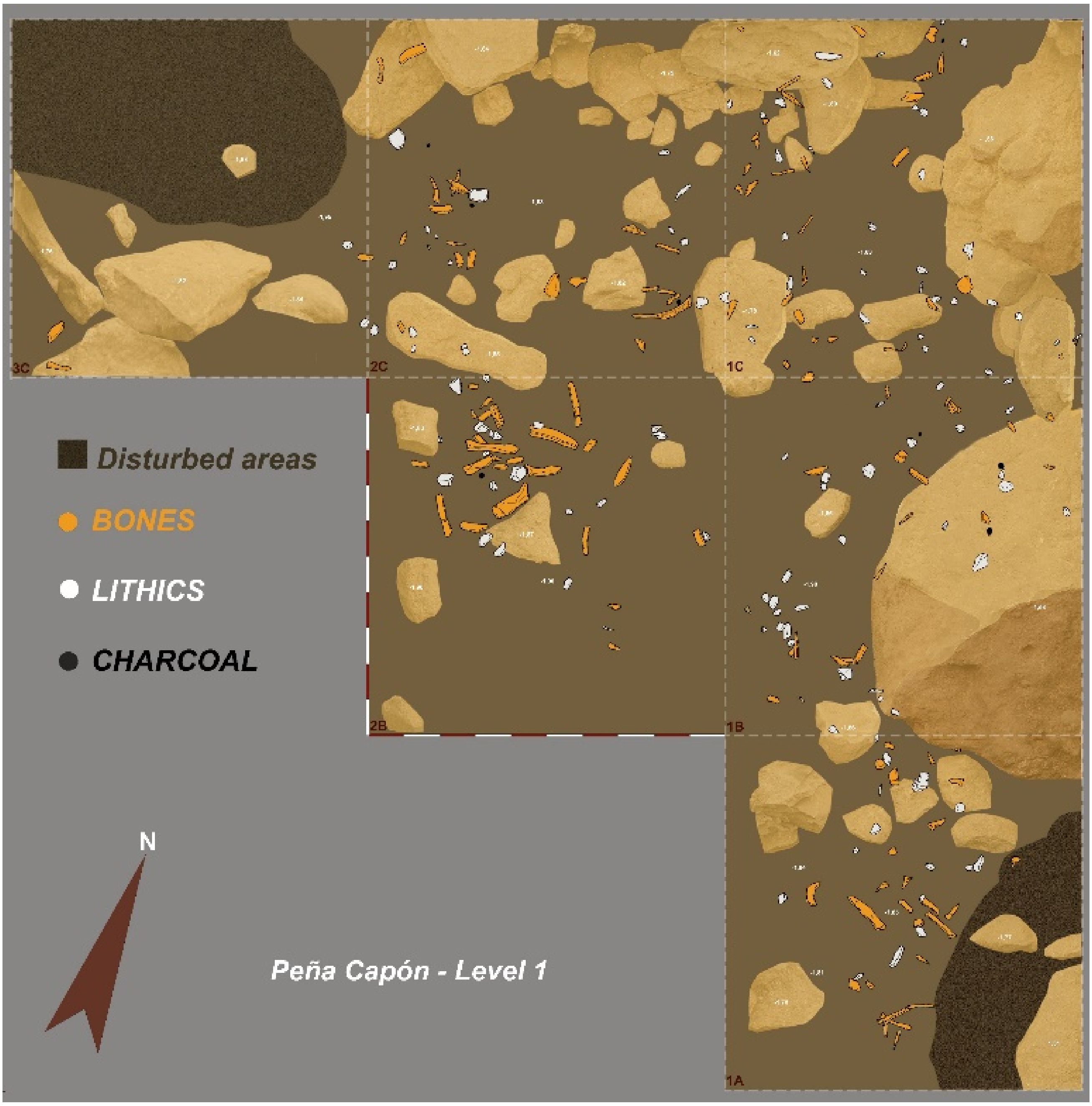

Figure S10. Plan of level 1 showing recorded archaeological items > 2 cm.

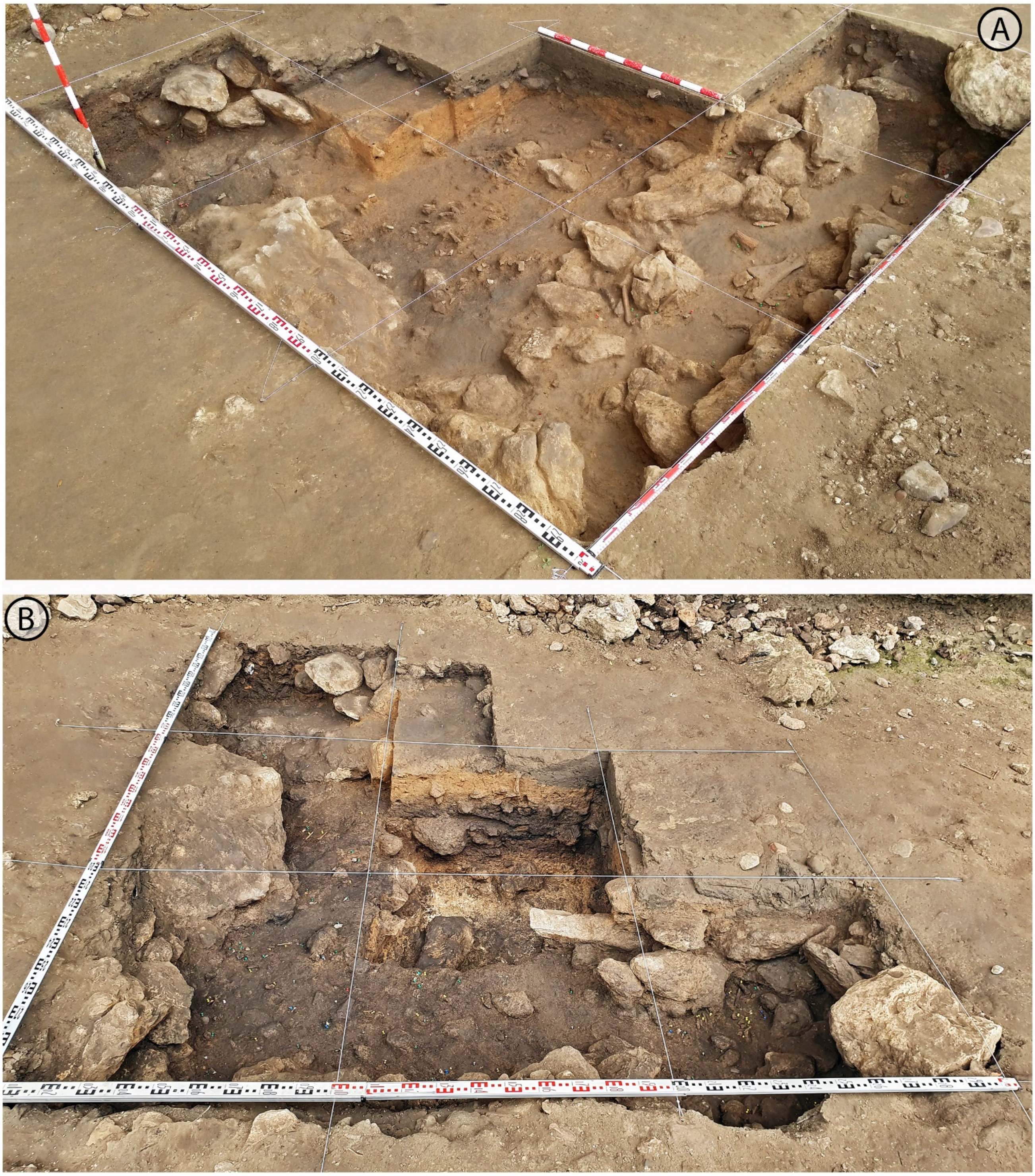

Figure S11. (A) Excavation area during the excavation of level 2a (layer 1). (B) Excavation area during the excavation of level 2b (layer 10).

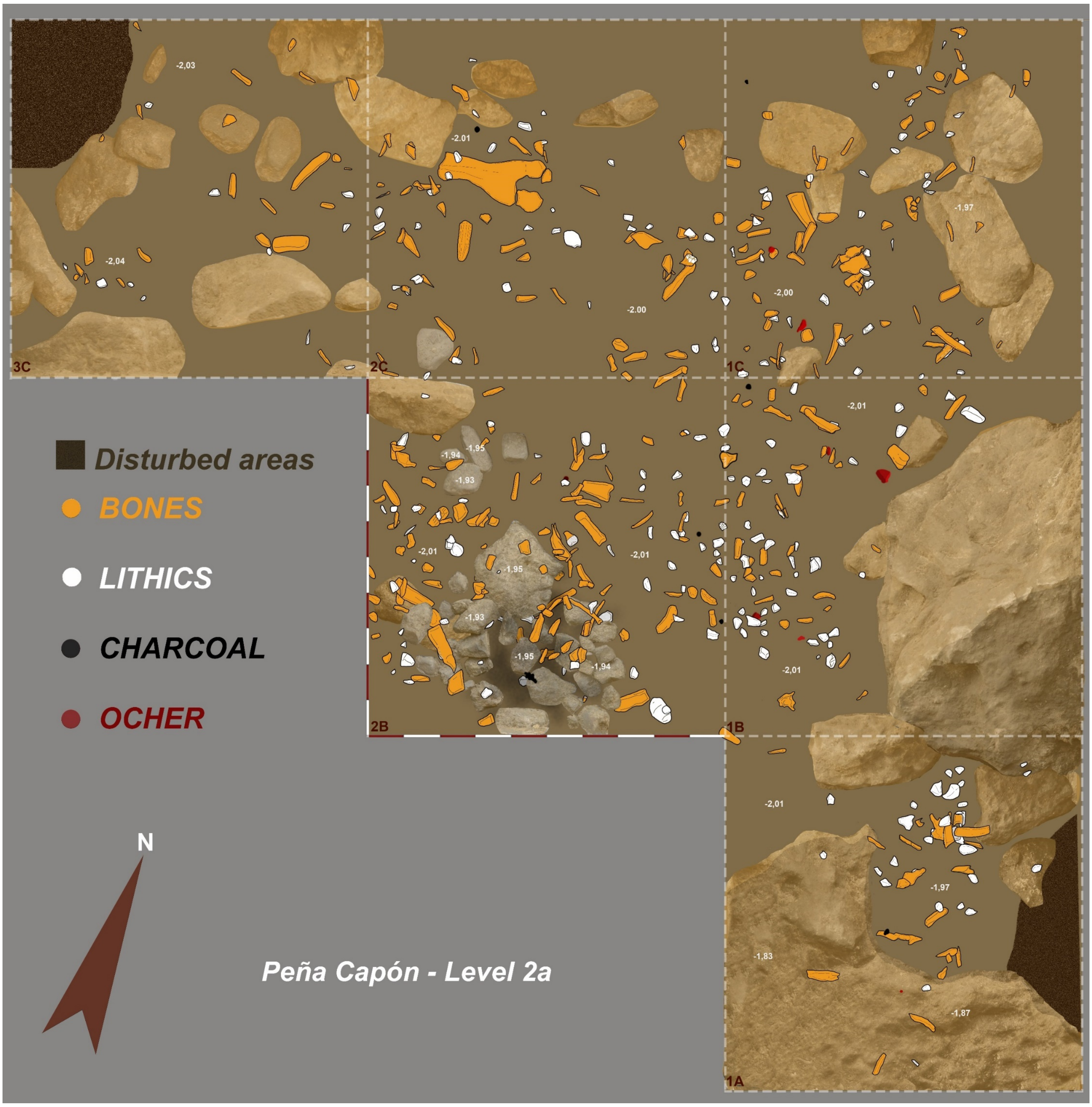

Figure S12. Plan of level 2a showing recorded archaeological items > 2 cm.

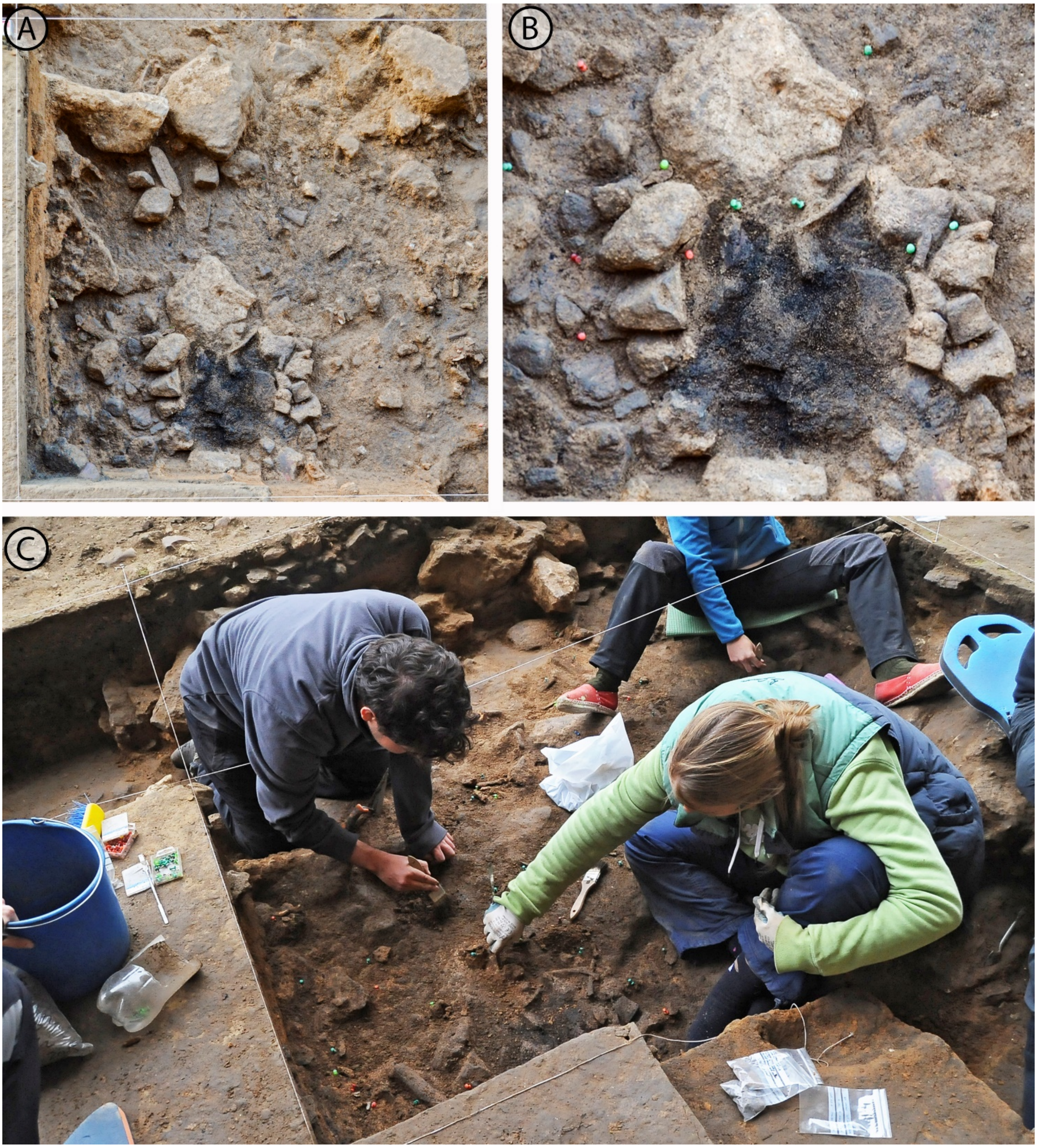

Figure S13. (A) Fireplace recorded in square 2B of level 2a (layer 3). (B) Detail. (C) Excavation process of the fireplace.

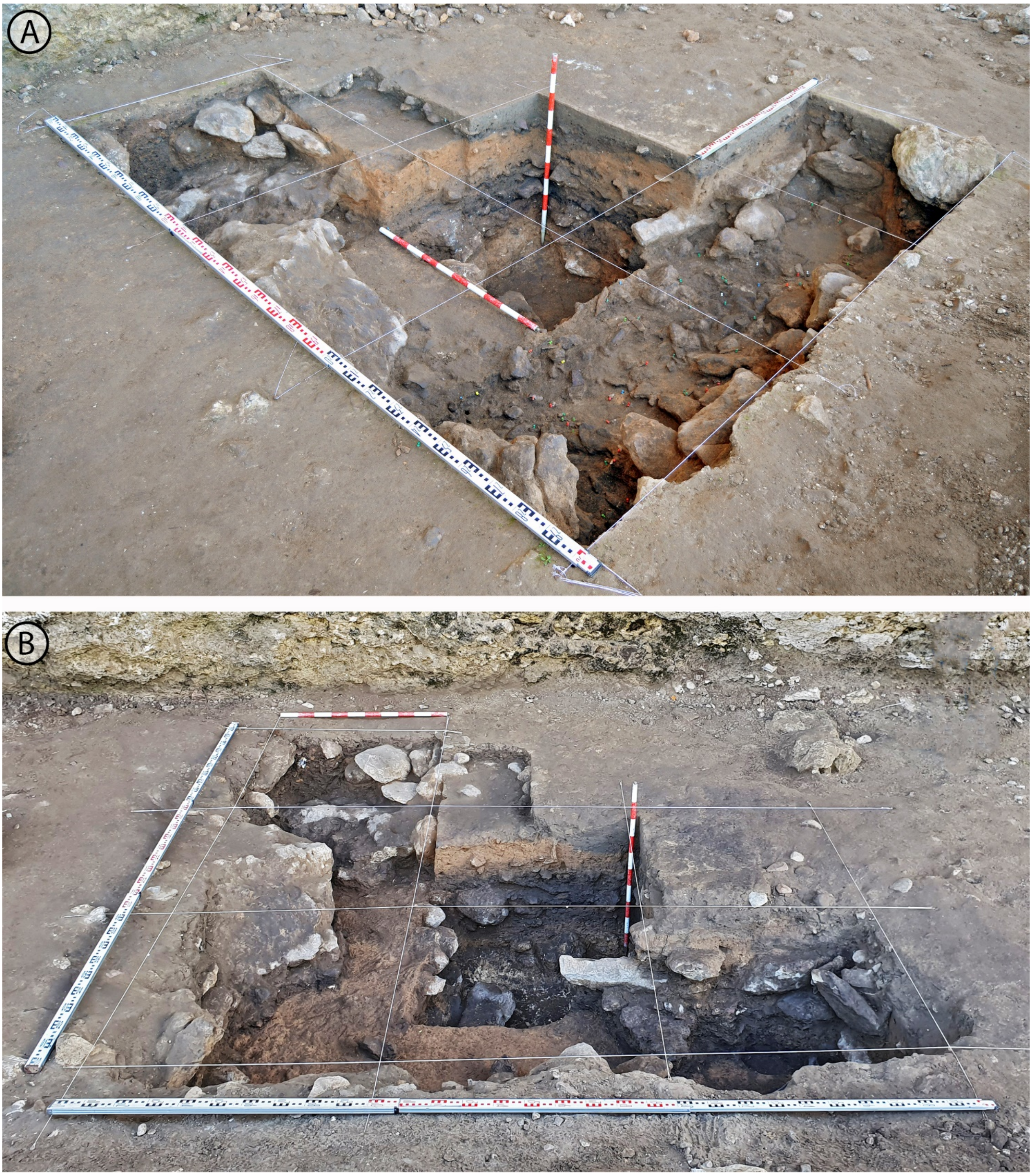

Figure S14. (A) Excavation area during the excavation of level 2b (layer 6; end of the 2015 season). (B) Excavation area during the excavation of level 3 (layer 2; end of the 2019 season).

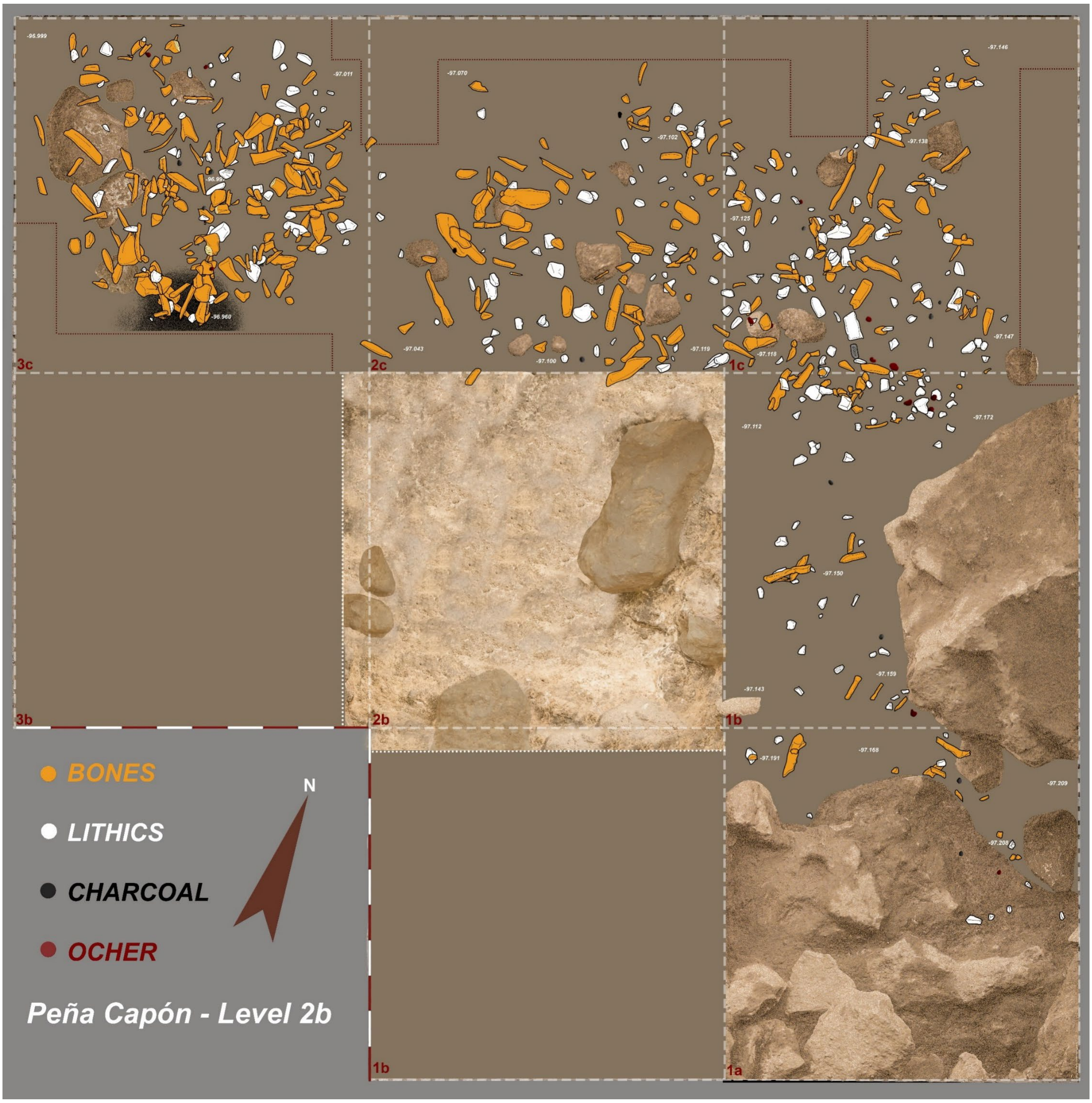

Figure S15. Plan of level 2b showing recorded archaeological items > 2 cm. Red dashed lines mark unexcavated areas due to the presence of stones and boulders within the profiles (see Fig. S16). In square 2B, a test pit was conducted up to level 6 in 2015. In 2019, the southern and western profiles of the test pit were refreshed, thus getting into squares 2A and 3B.

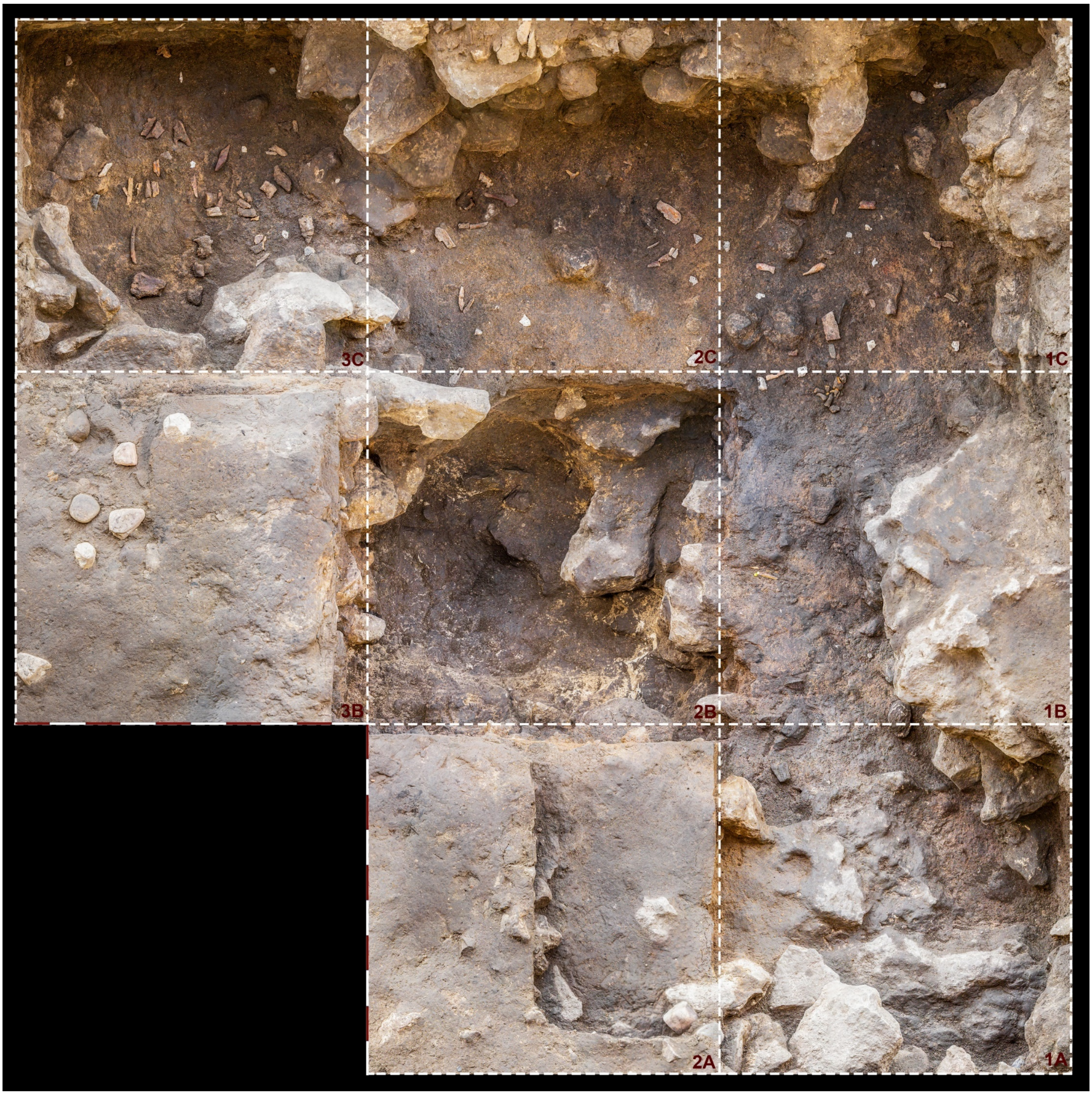

Figure. S16. Orthomosaic showing the base (layer 12) of Level 2b. Orthomosaic generated using Agisoft Metashape Professional v.1.5.2. (https://www.agisoft.com/) and Adobe Creative Cloud (Photoshop v. 22.4.2. and Bridge v.11.1) (https://www.adobe.com/es/creativecloud.html).

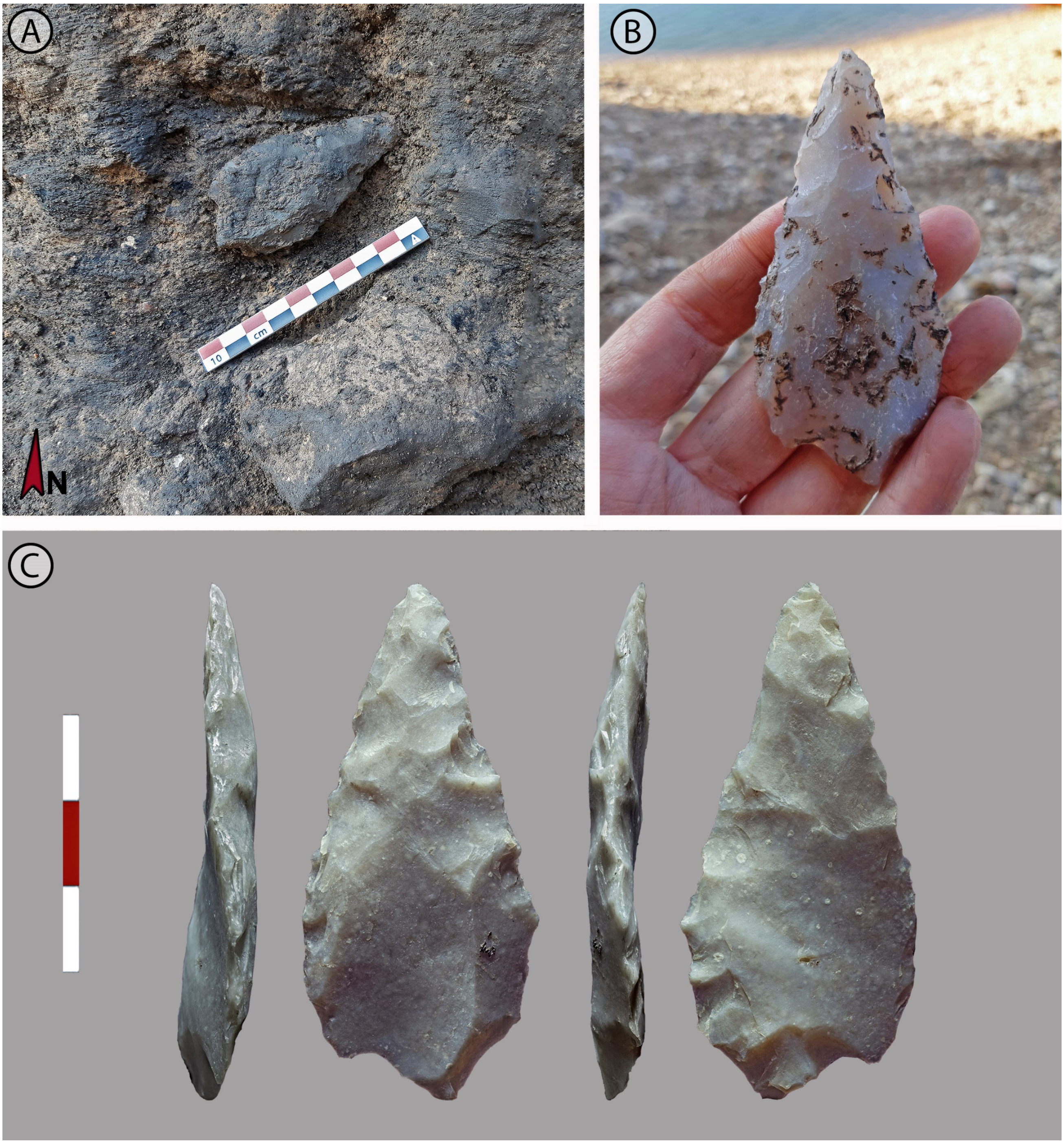

Figure S17. Preform of foliate point in an advanced stage of reduction from level 2b. (A) View during the excavation process of layer 8 of level 2b. (B) View after first cleaning at the site. (C) View after removal of adhering concretions and photography at the lab.

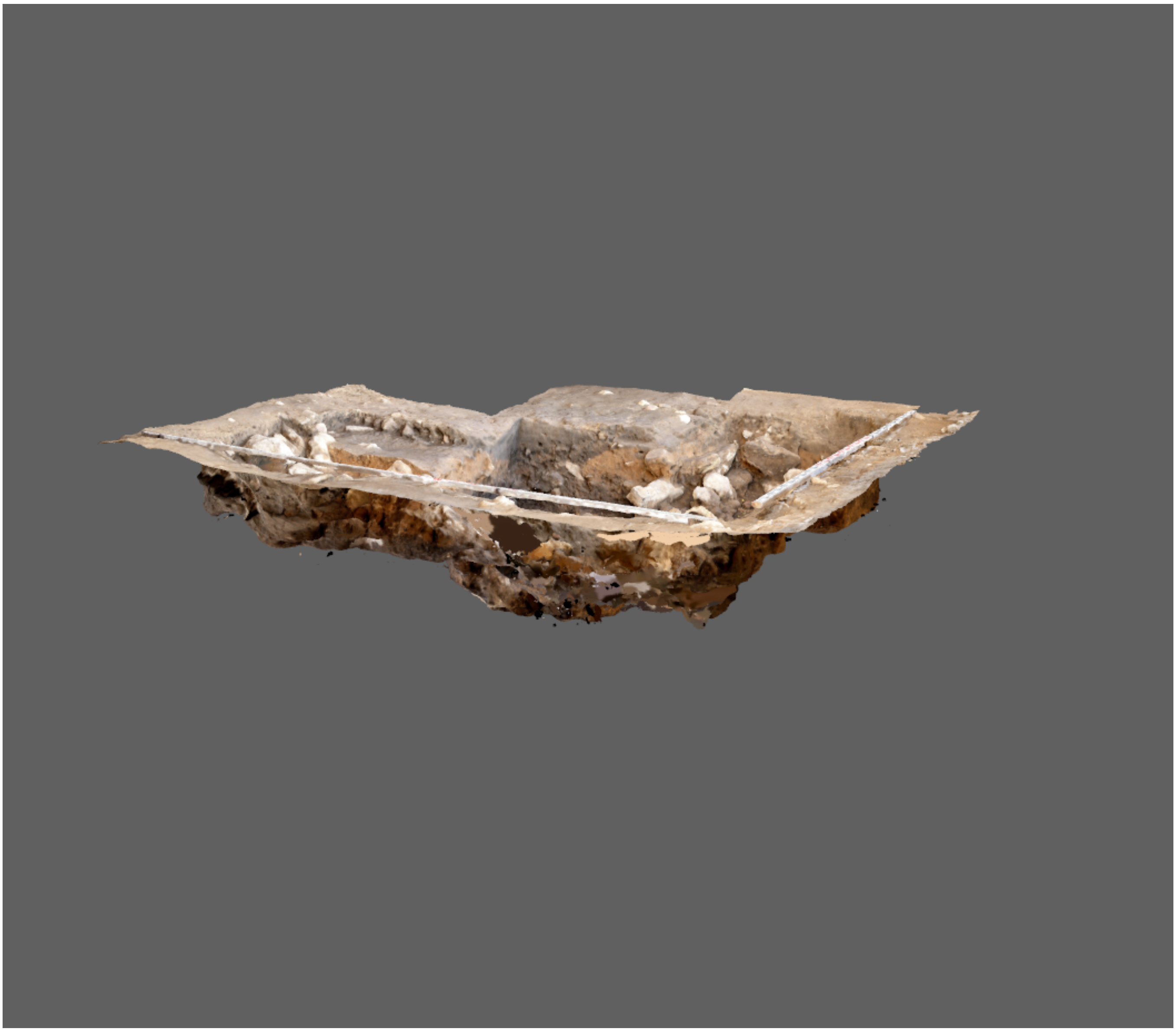

Figure S18. 3D Model of the excavation area at the base (layer 12) of Level 2b. (a PLY file containing a high-resolution model can be downloaded from https://multipaleoiberia.com/). Model generated using Agisoft Metashape Professional v.1.5.2. (https://www.agisoft.com/).

S1.3 – The modern human settlement of the Iberian Peninsula

Text S3. Criteria for considering radiometric dates and archaeological sites as valid markers for human presence at a given time

In Figure 1 of the main text we present a state-of-the-art view of the progressive modern human settlement of the Iberian Peninsula. The process of peopling has been divided in four steps, representing a north-south and outwards-inwards trend, with inland Iberia, being the focus of our research, as the latest macro-region to be populated according to current data. Considering the Aurignacian as a reasonable proxy for modern human agency, the first map (A) starts with the first record of Aurignacian assemblages in the north of Iberia at ~42 ka cal BP, representing the first arrival of modern humans to Iberia. The second step (map B) spans from~38 to 30 ka cal BP, and represents the dispersal of modern humans to the rest of Iberia along the coasts, with

only minor and sparse incursions into interior areas of the peninsula, being the Spanish plateau still totally unpopulated. The third step (map C), between ~30 and 25 ka cal BP, shows the first known settlement of inland Iberia by modern humans, with clusters of settlement in the west and north borders of the northern *Meseta*, and the single site of Peña Capón as recording the first presence of modern humans in the deep interior of the Iberian Peninsula. Finally, the fourth step (map D) represents an intensification of the human settlement of inland Iberia during Solutrean times (~25 and 20 ka cal BP).

In all four maps presented, we included only sites with reliable chronometric dates (calibrated using IntCal20; Reimer et al. 2020), secure archaeo-stratigraphic contexts and clear cultural attributions. A number of dates were excluded according to one or several of these criteria. However, considering the scarcity of data in inland Iberia, in map D we also included sites with undated Solutrean assemblages and pre-Magdalenian rock art as proxies for modern human presence in these interior territories. These two types of chronometrically undated sites could have been also included for the rest of the peninsula. However, it was not deemed necessary given that modern human presence in those areas is well established considering just radiometrically-dated sites. Since this is not yet possible for inland Iberia, for the sake of discussion we included these sites in the map, as we consider them reliable coarse-grained chronological markers.

A complete database of sites, numbered as represented in maps of Figure 1 of the main text, can be found in Supplementary datasets 1 to 4. In these datasets, associated to maps A to D respectively, we provide geographical coordinates, cultural attributions, lab codes, 14C data and calibrated dates for each layer or site considered. We also provide bibliographic references for each of them.

SI.4 – Geomorphology, sedimentology and micromorphology

Text S4. Methods: geomorphology, sedimentology and micromorphology

For a geomorphological contextualization of the site we analyzed both the most recent satellite imagery (PNOA flight 2018) and historical aerial photographs using the QGIS Geographic Information System (Open Source Geospatial Foundation Project) version 3.4 (Madeira). The latter has been especially relevant to interpret the Quaternary deposits nowadays flooded by the Beleña reservoir waters. The flights were made in 1956 and 1980 while the dam was built in 1982. To map the Quaternary deposits we conducted a detailed field survey and processed the geological maps from the Spanish Geological Survey (WMS on line access) with several GIS tools. These included maps of slopes, hill-shades, and heights based on digital elevation models (DEM) from the PNOA-LiDAR (National Plan of Aerial Ortophotography) of the Spanish National Geographical Institute, with one point every square meter and a height accuracy better than 0,2 m (RMSEz= 17.1 cm).

The sedimentological analyses have been carried out both during the fieldwork and in the Laboratory of the Institute of Geography of the University of Cologne (Germany). The sediment sequence exposed in square 2B was studied in more detail to gain a deeper insight into processes of sediment accumulation and post-depositional change as well as sediment composition. The western profile was chosen for sampling. A set of 28 sediment samples were taken along a vertical line on the right-hand side of the profile (Fig. 4 in the main text). Vertical sampling interval was 5 cm, except for levels R0 and R1, from which one sample each was extracted. Due to technical reasons, level 6 was not included in the sampling. The samples were oven-dried at 40°C. The size fraction <2mm in diameter was obtained by dry-sieving and all further analyses were conducted on this fraction.

Particle size distributions of samples were measured using a Beckman-Coulter LS 13320 PIDS laser diffraction particle size analyzer with a measuring range from 0.04- 2000 µm diameter. Removal of soil organic matter was accomplished with hydrogen peroxide (30%) and the dispersion of grains by addition of $Na_4P_2O_7$ (0.1 mol*l^{-1}) and overhead shaking for 12 h. Calculation of grain size percentages was based on Mie theory (Fluid

RI: 1.33; Sample RI: 1.55; Imaginary RI: 0.1). The granulometric parameters sand, silt and clay were calculated using the statistical package GRADISTAT v 8.0 (Blott and Pye, 2001).

Sediment color of the mechanically homogenized dry samples was recorded using a Konica Minolta CM-5 spectrophotometer following a standardized procedure (2° standard observer and illuminant C) according to Eckmeier et al. (2013). The spectral range in the visible light from 360 to 740 nm was measured and the obtained spectral information was converted to the CIE (L*, a*, b*) color space utilizing the SpectraMagic NX software (Konica Minolta). In this paper, the L* and a* values are discussed. The L* values give the luminance of the sample material ranging from L*0 (maximum extinction of light, black) to L*100 (maximum luminance, white). Positive a* values indicate redness, while negative values represent greenness.

Measurements of carbonates and total carbon were carried out after grinding part of the < 2mm size fraction in a vibrating zircon ball mill (Mixer Mill MM 400, Retsch). The carbonate content was determined by measuring the CO_2 released by gas pressure calcimetry after addition of HCl and expressed as calcium carbonate equivalent (CCE). Inorganic carbon (IC) was calculated by dividing CCE by 8.3331. Total carbon (TC) was measured via elemental analyses in a vario EL cube (Elementar Analysensysteme GmbH). The total organic carbon (TOC) was calculated by TOC = TC-IC.

Mass specific magnetic susceptibility was measured on about 15 g of bulk samples at low frequency (0.47 kHz; χlf) using a Bartington MS2B dual frequency sensor (Dearing, 1999).

The micromorphological investigations were done on thin sections subsampled from six sediment blocks taken from levels R0 down to level 5 as shown in Fig. 4B of the main text. In the field, the blocks were reinforced with gypsum bandages to avoid collapse and loss of the original structure (Fig. S20). The blocks were dried at 40°C for more than ten days and impregnated with polyester resin under vacuum. After at least six weeks of resin hardening, the blocks were subsampled using a rock saw. Eighteen uncovered thin sections, 80mm x 60mm large and approximately 25µm thick were prepared by grinding and polishing. All preparations were carried out by Th. Beckmann, Schwülper-Lagesbüttel, Germany. The micromorphological analysis of the thin sections was accomplished at different magnifications, using flatbed scans at a resolution of 1200dpi (< 20x), and a polarizing microscope (12.5x to 500x). Microscopic inspection utilized plane-polarized light (PPL), crossed polarizers (XPL) and oblique incident light (OIL). The description of thin sections followed the guidelines of Stoops (2003).

Text S5. Detailed sedimentological and micromorphological description of the archaeological deposit

The Peña Capon stratigraphic sequence is capped by a heterogeneous unit of dark grey sandy loam, containing dolomite clasts plus some quartz and mica grains and plant remains in a sandy to silty matrix with limited amounts of clay. It lies unconformably on the archaeological deposit, overlying an erosive surface. This disturbed layer has been defined as Level R and can be roughly divided into two subunits being the lower one (R0) darker, more coarse-grained, richer in quartz, mica, charcoal, bone fragments and carbonate nodules than the uppermost one (R1). The Upper Palaeolithic archaeological deposit is divided in six levels (Level 1 to Level 6).

Level 1 is found in all excavated squares, showing a maximum thickness of 28 cm and dipping westward. The upper contact with the overlaying Level R is very irregular and erosive. Level 1 consists of reddish-brown sandy silt, moderately sorted, containing scarce charcoal and bone fragments (rarely larger than few millimeters). Sub-angular to sub-rounded gravitational blocks of dolomite derived from the rock wall are abundant. This level is generally massive but in some parts of the profiles three sub-levels can be identified. The lower one contains larger limestone clasts and three to four thin black levels rich in organic matter and charcoal. The intermediate is more massive but with smaller angular clasts of carbonate. The upper one is more reddish in

color and with fewer dolomite fragments. This latter sub-unit outcrops scarcely and normally between rock blocks of dolomite derived from the cliff. The whole Level 1, but also the other levels, shows abundant bioturbation, normally vertical tubes around 1-1.5 mm in diameter that in some cases show a thin microcrystalline coat of micritic calcite. There are few carbonate crystals but carbonate cement and nodules are present, suggesting the diagenetic lixiviation of the sediment. Level 1 has a sharp to locally gradual contact with the underlying Level 2.

Level 2 is recorded homogeneously in the whole excavated area and shows a thickness ranging from 14 to 21 cm. It consists of dark brown to black color sandy silt, rich in charcoal fragments, carbonaceous matter, bone fragments and dolomite rock fragments and blocks derived from the rock shelter wall. It can be divided into two sub-levels: 2a on top, contains less amount of charcoal and shows some lamination; 2b below, has a larger amount of charcoal and organic matter, being darker in color. The whole Level shows abundant archaeological remains and charred bone fragments. Bioturbation and secondary carbonate (sometimes as micrite) are frequent. Dolomite gravitational blocks are also common and sometimes show thermal alteration. The contact with the underlying Level 3 is sharp and irregular, showing a small pit filled with very dark silty sediment, bones and charcoal, being very rich in organic matter, in the western part of the excavated area. Some burrowing probably caused by small mammals has also been identified in this contact.

Level 3 has only been recorded in the north and west part of the excavation. Its maximum thickness is just 5 cm, thinning south and westwards and gradually disappearing in north-eastward direction. It consists of reddish-brown sandy silt, very similar to that of Level 1, and it is very homogeneous and massive, with few charcoal and bone fragments. It contains a lot of diffuse secondary calcite in a micro-scale, both as porosity infill and carbonate nodules. Dolomite gravitational subangular blocks are common. The lower part of this level is richer in organic matter and shows a gradual contact with the underlying Level 4.

Levels 4 to 6 have only been excavated in the test pit conducted in square 2B. Level 4 shows an irregular thickness (4 to 17 cm) and color variations that range from reddish-brown to a more intense reddish tone, suggesting rubefaction processes, especially where it underlies Level 2. It has a high organic matter content. It looks massive in the field, but it is layered under the microscope. It contains small fragments of charcoal, iron oxide traces and abundant bioturbation tubes. The lower part has a gradual to sharp contact with the underlying Level 5.

Level 5 is very similar to Level 1 and Level 3 but lighter in color and more cemented by diffuse secondary carbonate. Its thickness varies from 12 to 27 cm. The deposit consists of reddish-brown sandy silt with scarce organic matter and a high number of fallen dolomite blocks. The lower part shows a clear increase in secondary carbonate content that makes the contact with the underlying Level 6 gradual.

Level 6 has a thickness from 5 to 20 cm and it consists of pale reddish-brown sandy silt with a high amount of secondary carbonate that most probably comes from the lixiviation of the large dolomite block or the substrate right below. Larger dolomite blocks are abundant in this Level and no internal structure has been identified in it. Its lower contact is irregular and very rich in carbonate.

The granulometric analyses of the sediment sequence at profile 2B-West displays a homogeneous textural composition dominated by sand and silt ranging from 33 to 48% and 44 to 57%, respectively (Fig. S19). The proportion of clay-size particles remains low with a maximum of 10 %. All samples are very poorly sorted and classify as very fine sandy, very coarse silt. The granulometric curve of level R0 is unimodal, whereas all other levels have bi- or trimodal frequency grain-size distributions. The D75/D25 ratio reflects the low variation in grain-size showing a slight trend of coarsening towards the top. The L*-value varies between 49.5 and 61.4 with higher values in the light-colored levels R0, L1, L2a, L3, and L5 and lower values in the darker ones (levels R1, L2b, L4). The color change with depth is well reflected by the a*-value indicating more reddish colors in

levels L1, L3, and L5. The carbonate content ranges from 10 to 32% attaining maximum values in Level R0. The comparatively high carbonate contents of L5 corroborate the field evidence. The carbonate peak in L2a probably reflects a more local enrichment with secondary carbonate. The TOC ranges from 0.3 to 2.9% and draws a mirror-inverted picture of the L*-value; all samples with high TOC values have low L* and vice versa. This shows that the color change is mainly related to variation in organic matter content. The magnetic susceptibility, χlf, is between 1.9 10^{-7} to 1.23 10^{-6} m^3 kg^{-1}. High values occur in the dark-colored, organic matter-rich levels R1, L2b and L4, i.e. levels with high amounts of charred organics. Magnetic susceptibility depends on the mixture of minerals and other components in the sample and is related to the type and grain-size of ferrimagnetic minerals present. These minerals can be of primary origin or may form by burning or soil formation (Dearing et al., 1999). At Peña Capón, processes of soil formation such as carbonate leaching, reprecipitation of secondary carbonate and precipitation of iron oxides will have affected each of the rapidly accumulated layers in a similar way. The variation in χlf over the levels makes it more likely that the magnetic signal has been induced by burning.

First observations on thin sections prepared for the sediment sequence of profile 2B-West have been presented previously (Alcaraz-Castaño et al., 2019). A summary of the micromorphological description is presented in Table S2 and figures showing selected thin section scans are included in Figure 5 of the main text). The thin sections show a mixture of natural and anthropogenic sediment components. Rock fragments consist of subangular dolostone derived from the cave wall and sub-rounded to rounded quartzite and fine siltstone gravel accumulated by fluvial transport. Few small angular pieces of chert, rock crystal and quartzite represent by-products of tool production (Angelucci 2010). Bone fragments are mostly well preserved but often show infillings or coatings of secondary calcite. Charcoal with well-preserved cell structures is common in dark-colored levels, where it occurs together with amorphous charred organic matter of unknown origin. Very few fragments of carnivore coprolite were identified. The granulometric composition of the fine material (< 2mm in diameter) is dominated by coarse silt and fine sand particles consisting of quartz, dolostone fragments, and mica. The so-called micromass, i.e. sub-microscopic particles smaller than about 5µm in diameter, is very few.

The levels show differences in groundmass color (Fig. 5 in the main text) which are mainly related to varying contents of charcoal and charred organic fines, as well as to local enrichments with secondary calcite. The uppermost level (R0) is rich in primary carbonate and contains many fresh plant remains including roots or twigs, but little charcoal. Horizontal lamination suggests sub-aqueous deposition, or at least reworking. Except for very few passage features, no post-depositional change is observed suggesting that the level represents a very recent deposit. Level R1 consists of poorly-sorted sands and silts with less calcite or dolomite sand grains than above and includes a small lens with charcoal. It has a moderately-developed platy microstructure and a high degree of compaction, which may be indicative of trampling (Rentzel et al., 2017). Thin sections of sediment from level L1 have a homogenous light orange-brown groundmass of silts and sands with little fine gravel and few small pieces of charcoal and bone (Fig. 5 A, and upper part of B). Level L1 has a low degree of compaction and abundant pores consisting of biogenic channels and burrows which form by roots or soil-dwelling mesofauna (Kooistra and Pulleman, 2018) indicating bioturbation on a microscale. Many of the pores are surrounded by micritic hypocoatings. In addition, few calcite nodules and infillings occur. These calcitic pedofeatures testify to precipitation of secondary carbonates (Durand et al., 2018) after lixiviation or carbonate leaching of larger blocks and fine sediments. Carbonate metabolism also affected the layers below in which similar calcitic pedofeatures occur. The following level L2 is more greyish in color and shows vertical and lateral variation in the contents of charcoal and charred organic matter. Level 2a has a grey-brown groundmass (Fig. 5 C) and contains several large fragments of bone and charcoal as well as chert and rock crystal fragments, clearly identified as by-products of lithic artifact production. In thin sections 6.1 and 6.2, originating from the left side of profile 2B, level 2a contains a charcoal-rich lens with abundant bone fragments (Fig. 5, B), partly showing abundant infillings of micrite. The dark-colored level 2b (Fig. 5, E and upper part of

F) has higher contents of charcoal and charred organic matter than L2a, but still shows some variation in composition and degree of compaction with depth. On a microscale, level 2 can thus be divided into five micromorphological sublevels as suggested by Alcaraz-Castaño et al. (2019), indicating that the level accumulated during a sequence of depositional events. In level 2, some fragments of limestone and quartzite occur that experienced heating, as indicated by a reddish appearance in reflected light. Calcitic pedofeatures as mentioned above occur throughout (Fig. 5, D), and locally the groundmass shows diffuse enrichment in micrite. Level 3 contains few charcoal, bone, or chert fragments and has a light-colored yellow-grey groundmass. Porosity is less than above and locally a massive microstructure is present. Diffuse micritic impregnations indicate considerable accumulation of secondary calcite (Fig. 5, F lower part). The content of charcoal increases again in level 4. Similar to level 2b, several heavily calcified pieces of bone are present. The top of level 4 is delineated by a thin line of small gravel (Fig. 5, G lower part). In its central part, oblique incident light elicits a reddish color from a 1cm-sized patch of the groundmass. This reddish patch probably represents burned soil. In addition, very few fragments of ochre, up to 2 mm in diameter, characterized by bright orange or red color under reflected light are present. The following level 5 has a lighter color and is strongly enriched with secondary calcite occurring as diffuse impregnations (Fig. 5, H upper part). Very few small ochre fragments occur. The lower part of ts 5.2 represents a level with higher contents of charcoal (Fig. 5, H lower part). Sediments from the lower part of level 5 and of level 6 could not be included in the micromorphological analyses due to high stone content and insufficient access.

In summary, sediments below level R0 share many sedimentological and micromorphological features, while a variation between different levels is mainly related to the content of burned components and bone. The dissolution of primary carbonate grains and the precipitation of secondary carbonate are the primary post-depositional processes. Very limited mobilization of phosphates is indicated by phosphatic rims on limestone fragments. The dominating channel and burrow microstructure testifies to intensive rooting and burrowing activity on a microscale. This bioturbation may have destroyed the primary depositional fabric of the fine sands and silts. Sediment boundaries between the main levels, and within level 2, are still well preserved. There is no micromorphological evidence for effects of ice-lensing or frost, which could be deduced from features such as platy or lenticular microstructure, banded fabric, silt cappings or vertically-oriented, elongated coarse grains (Kehl et al., 2013; 2018; Mallol et al., 2017; Van Vliet Lanoë and Cox, 2018).

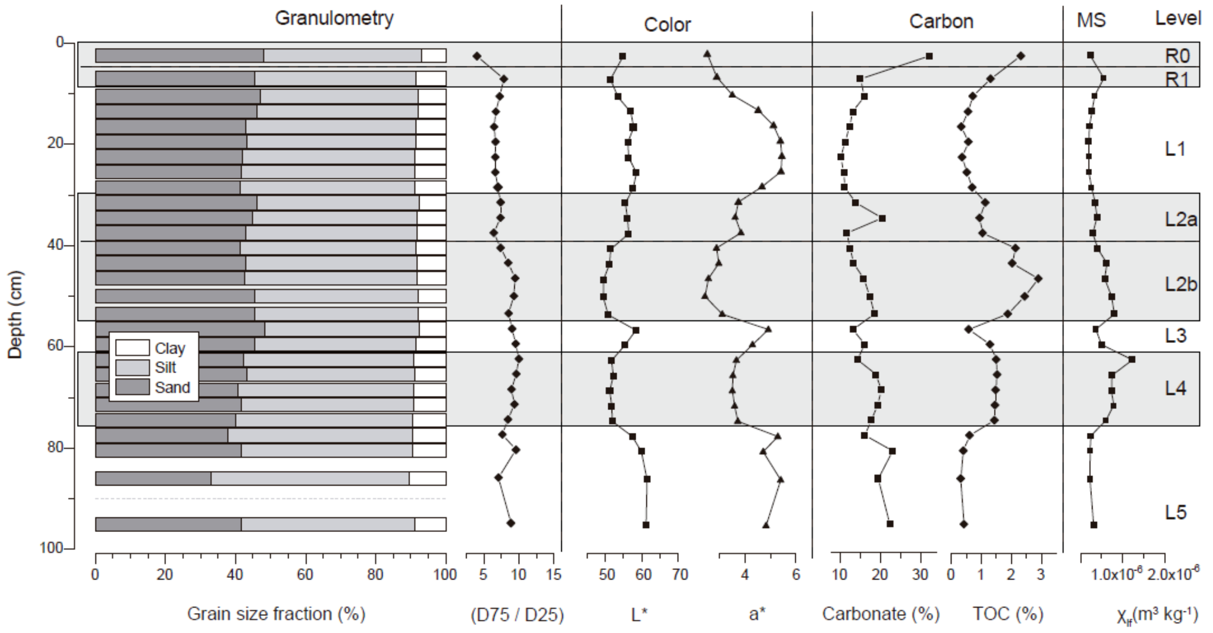

Figure S19. Sediment properties of the western profile of square 2B (for sampling location see Fig. 4 of the main text).

Level	ts #	Mineral grains	Organic materials and bone	Color	Pore space and fabric	Pedofeatures
R0	1.1	Sandy silt consisting of carbonates, quartz, few mica	Many fresh small twigs and other plant remnants, few small bone fragments, one piece of charcoal	Grey	Simple packing voids; laminated, sharp lower boundary	Very few passage features
R1	1.1l; 1.2u	Sandy silt with few mid-size limestone grit and fine dolomite and siltstone gravel	Few charcoals and bones	Grey-brown	Simple and compound packing voids; layered, moderately developed platy ms, sharp lower boundary	Many calcite hypocoatings, few calcite nodules and incomplete infillings
L1	1.2l, 2.1, 2.2, 2.3u, 3.1l, 6.1u	Sandy silt with few fine gravel of dolomite, quarzite and siltstone, very few grit increasing in proportion in ts 2.3	Very few to few small pieces of charcoal and bone	Orange-brown	Many burrows, channels, chambers, few compound packing voids; channel and burrow ms, partly subangular blocky ms with small rounded peds; large root channel in ts 2.2; clear lower boundary	Many calcite hypocoatings, few calcite nodules and incomplete infillings, large diffuse calcite impregnation in ts 6.1
L2a	2.3l, 3.1l 3.2, 3.3u 6.1lower, 6.2	Sandy silt similar to L1, but more mica, few fine grit and gravel, presence of chert fragments; heated fragment of limestone	More large fragments of charcoal and bone than in level L1; charcoal and bone-rich lenses in ts 6.1 and 6.2	Grey-brown, locally dark grey	Many burrows and channels, very few compound packing voids; channel and burrow ms, gradual lower boundary	Many calcite hypocoatings and nodules; large impregnation with secondary calcite in ts 6.2, calcite infillings in several bones; phosphatic rim on limestone
L2b	3.3l, 3.4, 4.1, 4.2u, 6.3l	Sandy silt with small to medium grit and few small gravel, rock crystal, quartzite, siltstone heated frag of quartzite	Many charcoals, few bones	Grey-brown, dark grey	Many burrows and channels; channel and burrow ms, locally spongy ms; sharp lower boundary	Many calcite hypocoatings and nodules as well as infillings in bone fragments
L3	4.2l, 4.3u	Sandy silt with one large grit, line of small gravel in the center, chert;	Very few charcoals and bones	Light yellow grey	Slightly fewer burrows and channels than above; channel and burrow, partly massive ms; clear lower boundary	Few calcite hypocoatings, several large diffuse calcite impregnations
L4	4.3l, 4.4, 5.1u	Sandy silt with many small gravel at the top, otherwise few fine grit and gravel; small fragments of chert, rock crystal, quartzite;	Many charcoal pieces, many bones, pieces of heavily calcified bone, less charcoal and bone at the base	Grey-brown	Many burrows and channels; channel and burrow ms, partly spongy ms; gradual lower boundary	Few calcite hypocoatings and diffuse impregnations; calcite infillings in bone
L5	5.1l, 5.2	Sandy silt with few fine and med gravel; reddish (OIL) groundmass suggest heating of soil	Less charcoal and bone than above, charcoal rich lens in the lower part	Light orange-brown and grey	Many burrows and channels, very few planes; channel and burrow, partly subangular blocky ms	Few calcite hypocoatings, several large diffuse calcite impregnations

Table S2: Summary table of the micromorphological description of thin sections from different levels at Peña Capón (ts #: thin section number; ms: microstructure; l = lower, u = upper part).

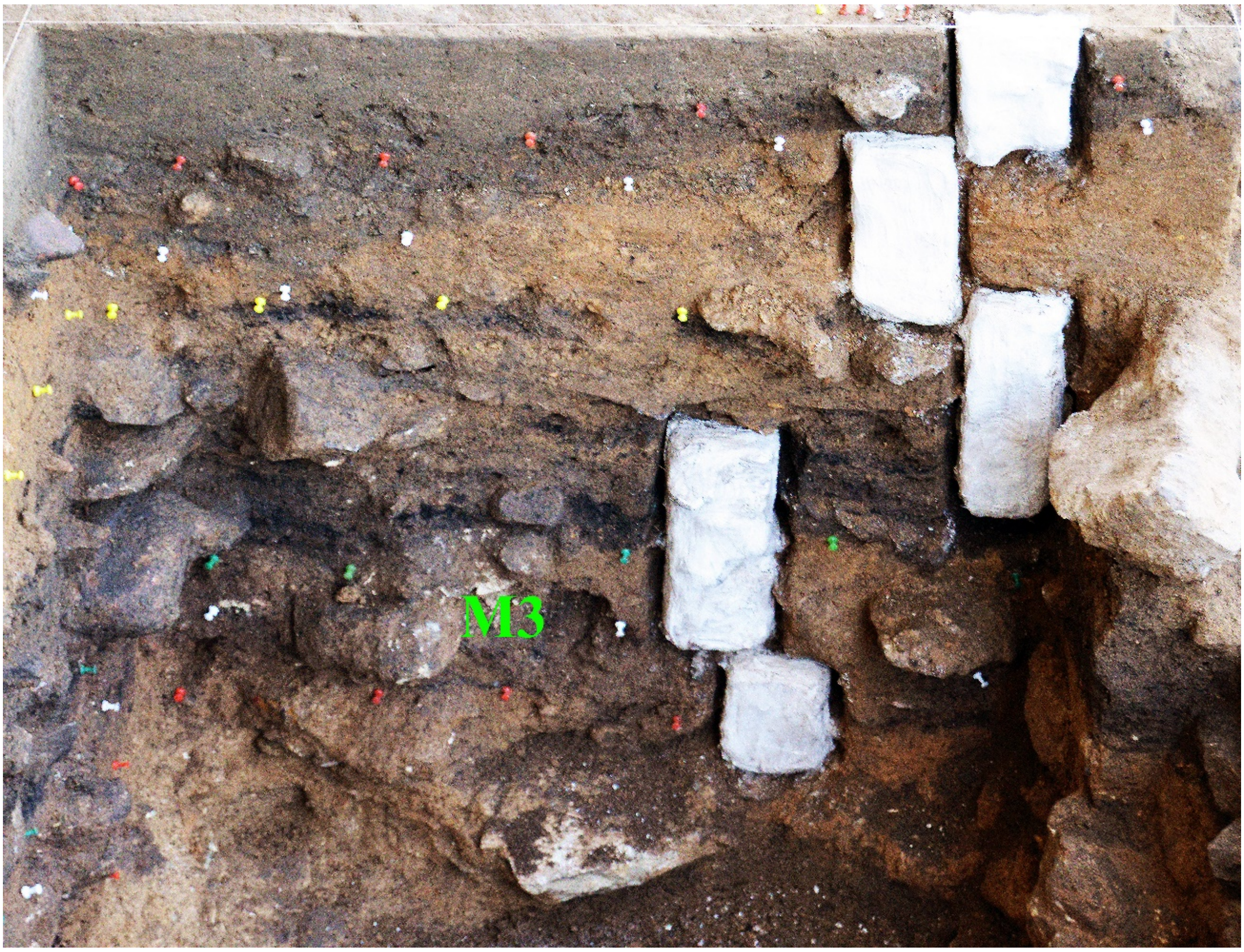

Figure S20. Western profile of square 2B showing sediment blocks reinforced with gypsum bandages prior to their extraction for micromorphological study. Location of pollen sample for level 3 (M3) is also shown.

SI.5 – Radiocarbon dating and Bayesian modeling

Level	Sample ID & material	Lab-ID	C^{14} BP	δ13C (‰)	Age cal BP (95,4%)	% C	Collagen yield (%)	Taxa
1 (Solutrean)	PCP Char 16-1	COL4210.1.1	20008 ± 112	-24.8	24371 - 23770	-	-	Angiosperm indet.
	PCP Bone 1/5	OxA-39498	19928 ± 97	-20.1	24195 - 23786	43.3	9.5	-
	PCP Bone 1/7	OxA-39505	19950 ± 110	-19.7	24220 - 23790	42.9	1	-
	PCP Char 16-2	COL4211.1.1	Modern	-25.4	-	-	-	Salix sp.
2a (Solutrean)	PCP Bone 16-1	COL4214.1.1	19987 ± 110	-17.9	24240 - 23804	41,6	5.0	-
	PCP Char 16-4	COL4213.1.1	20107 ± 111	-24.6	24468 - 23843			Angiosperm indet.
	PCP Bone 16-2	COL4215.1.1	20261 ± 111	-21.8	24675 - 23970	41,7	14.0	-
	PCP Bone 2/3	OxA-39499	20370 ± 110	-19.19	24871 - 24175	43.5	5.47	
	PCP Char 16-3	COL4212.1.1	20278 ± 107	-30.4	24700 - 24012	-		Angiosperm indet.
2b (Solutrean)	PCP Bone 2/5	OxA-X-3048-17	20308 ± 150	-19.19	24903 - 23974	43.7	0.5	-
	PCP Char 2/5	OxA-39506	20399 ± 63	-24.82	24747 - 24225	69	19.3	-
	PCP Bone 2/7	OxA-39500	20450 ± 110	-20.19	24956 - 24239	43.3	1.1	-
3 (Solutrean)	PCP Bone 16-3	COL4217.1.1	20006 ± 107	-18.1	24252 - 23811	36,9	1.0	-
	PCP Char 16-5	COL4216.1.1	20950 ± 128	-22.7	25655 - 24980	-	-	Angiosperm indet.
	PCP Bone 3/5	OxA-39501	20910 ± 110	-19.59	25608 - 24963	44.8	5.9	-
4 (Pre- Solutrean)	PCP Bone 4/Rect	OxA-39502	20930 ± 110	-19.47	25618 - 24991	44.4	4.3	
	PCP Bone 4/Rect3B	OxA-39750	20760 ± 110	-19.40	25283 - 24664	43.9	1.8	
	PCP Char 16-6	COL4218.1.1	21007 ± 118	-19.6	25671 - 25079			Indet.
	PCP Bone 16.10-1	COL-	Failed: Low yield	-	-	-	-	-
5 (Pre- Solutrean)	PCP Char 16-7	COL4219.1.1	20905 ± 118	-27.8	25623 - 24937	-	-	Angiosperm indet.
	PCP Bone 5/Rect2A	OxA-X-3058-10	20950 ± 180	-18.93	25730 - 24794	43.7	0.4	-
	PCP Bone 5/Rect3B	OxA-39749	21670 ± 130	-19.29	26280 - 25745	43.5	3.9	-
	PCP Bone 16-4	COL4220	Failed: no yield	-	-	-	-	-
	PCP Bone 16.10-2	COL-	Failed: no yield	-	-	-	-	-
	PCP Bone 5/4	P-48038	Failed: very low yield	-	-	-	-	-
	PCP Bone 5/4/1	P-48039	Failed: very low yield	-	-	-	-	-
6 (Pre-Solutrean)	PCP Char 16-8	COL4221.1.1	21591 ± 124	-27.5	26069 - 25669	-	-	Angiosperm indet.
	PCP Char 16-9	COL4222.1.1	21593 ± 121	-24.8	26065 - 25675	-	-	Conifer
	PCP Bone 6/1	P-48041	Failed: very low yield	-	-			-
	PCP Bone 6/2	P-49063	Failed: no yield	-	-	-	-	-
	PCP Bone 6/BN	P-49064	Failed: no yield	-	-	-	-	-
	PCP2 Char 6/1	P-49065	Failed: very low yield	-	-	-	-	-
	PCP2 Char 6/3	P-49066	Failed: very low yield	-	-	-	-	-

Table S3. Sampled materials and radiocarbon dates from the Peña Capón sequence. C^{14} dates were calibrated with OxCal 4.4 (Bronk Ramsey 2009a) using IntCal20 (Reimer et al. 2020). OxA-, OxA-X- and P- are ORAU refs, the last corresponding to failed samples. COL- are CologneAMS refs. Some charcoal fragments could not be identified. All bones were cut-marked shaft fragments of unidentifiable macromammals, most probably herbivores. Failed attempts are shown in red.

Name	Unmodeled (cal BP)				Modeled (cal BP)				Indices<br>Amodel= 53.2<br>Aoverall= 51.2		
	68.2% probability range		95.4% probability range		68.2% probability range		95.4% probability range				
	from	to	from	to	from	to	from	to	A	P	C
End Level 1 Boundary					23984	23782	24110	23620			99.8
COL4210.1.1	24140	23876	24260	23806	24054	23872	24150	23830	116.6	98.4	100
OxA-39498	23968	23830	24092	23806	24012	23852	24108	23826	89.8	98.2	99.9
OxA-39505	24090	23836	24220	23790	24038	23868	24142	23820	116.4	98.4	99.9
↑Level 1 Phase											
Boundary Level2a/Level 1					24170	23956	24234	23884			100
COL4214.1.1	24116	23864	24240	23804	24248	24068	24310	23948	76.5	96.9	100
COL4213.1.1	24238	23938	24466	23842	24278	24094	24380	23966	110.2	98	100
COL4215.1.1	24530	24190	24676	23970	24340	24152	24476	24050	114.9	98	100
OxA-39499	24628	24270	24878	24174	24358	24188	24506	24112	89.2	96.8	100
COL4212.1.1	24542	24210	24700	24012	24340	24162	24484	24066	109.4	97.9	100
↑Level 2a Phase											
Boundary Level 2b/Level 2a					24468	24250	24592	24192			99.9
OxA-X-3048-17	24624	24188	24904	23970	24612	24332	24830	24252	115.5	97.6	99.8
OxA-39506	24614	24340	24746	24226	24610	24370	24734	24274	108.8	98	99.8
OxA-39500	24770	24330	24958	24238	24654	24336	24874	24276	111.5	97.9	99.7
↑Level 2b Phase											
Boundary Level 3/Level 2b					25110	24384	25164	24340			99.5
COL4217.1.1<br>Posterior Outlier: 81%	24134	23878	24252	23810	25202	24450	25226	24410	4.6	18.7	99.5
COL4216.1.1	25550	25100	25654	24980	25192	25014	25272	24850	83.2	96.2	100
OxA-39501	25344	25028	25608	24962	25190	25012	25268	24868	99.8	96.8	100
↑Level 3 Phase											
Boundary Level 4/Level 3					25242	25100	25314	25006			100
OxA-39502	25514	25068	25618	24990	25286	25146	25410	25070	139.1	98.4	100
OxA-39750	25204	24902	25286	24660	25270	25136	25366	25046	74	96.2	100
COL4218.1.1	25560	25194	25672	25078	25294	25152	25432	25080	110.3	98.1	100
↑Level 4 Phase											
Boundary Level 5/Level 4					25358	25170	25534	25122			99.9
COL4219.1.1	25350	25012	25626	24932	25596	25254	25660	25190	65.9	96.2	99.9

OxA-X-3058-10	25568	25086	25730	24792	25632	25334	25730	25208	101.1	97.2	100
OxA-39749 (Posterior Outlier: 14%)	26030	25810	26276	25746	25888	25726	25948	25370	59.4	85.5	100
↑Level 5 Phase											
Boundary Level 6/5					25928	25786	26000	25578			99.9
COL4221.1.1	25964	25790	26070	25668	25954	25836	26012	25770	120.8	98.5	99.9
COL4222.1.1	25964	25794	26070	25672	25954	25836	26012	25770	120.1	98.5	99.9
↑Level 6 Phase											
Start Level 6 Boundary					26026	25854	26242	25778			99.7

Table S4. Detailed results of Bayesian Model 1 (Preliminary model) (Fig. S21). Modeled calibrated ages and Boundaries with the 68.2% and 95.4% probability ranges. Calculated with OxCal 4.4 (Bronk Ramsey et al. 2009a), using a General t-type Outlier Model (Bronk Ramsey 2009b) and IntCal20 (Reimer et al. 2020). Posterior outliers >5% and Agreement indexes <60 are shown in red; these samples were excluded before running Model 2.

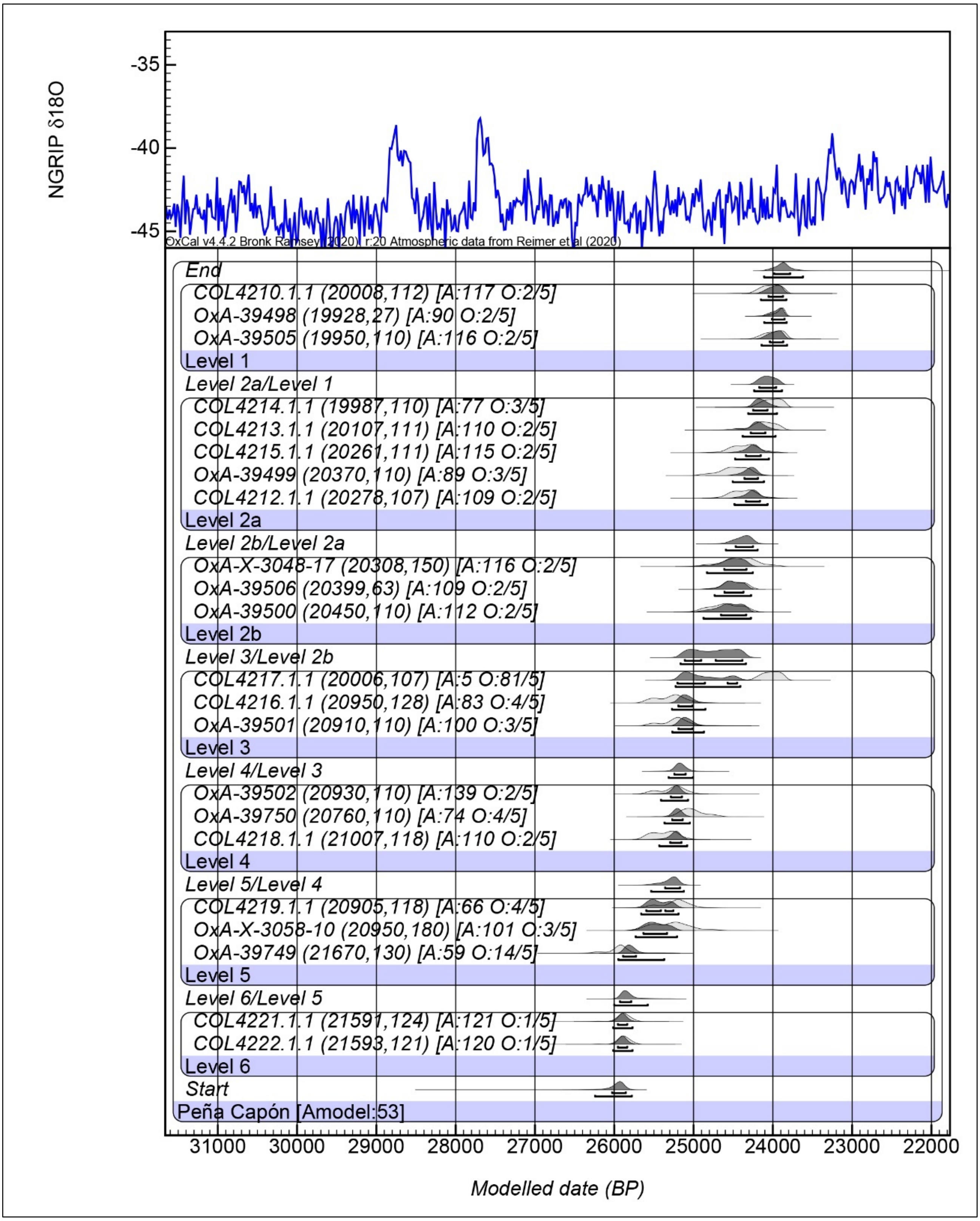

Figure S21. Bayesian Preliminary Model (1) for the Peña Capón sequence showing Probability Distribution Functions (PDFs) for all radiocarbon determinations and boundaries between archaeological levels. Radiocarbon dates were calibrated using IntCal20 (Reimer et al. 2020). The model and boundaries were calculated with OxCal 4.4 (Bronk Ramsey et al. 2009a), using a General t-type Outlier Model (Bronk Ramsey 2009b). 14C dates are shown in parentheses; Agreement indexes and Outliers' prior and posterior probabilities are shown in square brackets.

Peña Capón	Unmodeled (cal BP)				Modeled (cal BP)				Indices		
	68.2% probability range		95.4% probability range		68.2% probability range		95.4% probability range		Amodel=107.6<br>Aoverall=103.8		
	from	to	from	to	from	to	from	to	A	P	C
End Level 1 Boundary					23984	23788	24122	23628			97.1
COL4210.1.1	24140	23876	24260	23806	24058	23872	24154	23832	114.2	96.3	99
OxA-39498	23968	23830	24092	23806	24016	23852	24112	23828	86.4	96.1	98.9
OxA-39505	24090	23836	24220	23790	24042	23866	24148	23822	113.6	96.2	99
↑Level 1 Phase											
Boundary Level2a/Level 1					24170	23950	24236	23884			98.8
COL4214.1.1	24116	23864	24240	23804	24248	24068	24306	23946	75.8	95.6	99.7
COL4213.1.1	24238	23938	24466	23842	24274	24094	24374	23958	109.1	96	99.7
COL4215.1.1	24530	24190	24676	23970	24330	24150	24474	24048	111.5	96	99.8
OxA-39499	24628	24270	24878	24174	24356	24180	24506	24110	85.3	95.4	99.6
COL4212.1.1	24542	24210	24700	24012	24340	24156	24482	24062	105.8	96	99.8
↑Level 2a Phase											
Boundary Level 2b/Level 2a					24464	24234	24594	24176			99.4
OxA-X-3048-17	24624	24188	24904	23970	24658	24364	24870	24268	106.1	95.8	99.7
OxA-39506	24614	24340	24746	24226	24646	24404	24760	24290	105.4	96	99.8
OxA-39500	24770	24330	24958	24238	24732	24394	24912	24308	110	96	99.8
↑Level 2b Phase											
Boundary Level 3/Level 2b					25186	24630	25220	24470			97.3
COL4216.1.1	25550	25100	25654	24980	25196	25040	25268	24916	90.5	95.6	99.1
OxA-39501	25344	25028	25608	24962	25192	25040	25258	24934	107.6	95.8	99.2
↑Level 3 Phase											
Boundary Level 4/Level 3					25236	25110	25302	25020			99.4
OxA-39502	25514	25068	25618	24990	25270	25150	25382	25070	139.3	96.3	99.3
OxA-39750	25204	24902	25286	24660	25264	25138	25338	25062	74.7	95.2	99.3
COL4218.1.1	25560	25194	25672	25078	25282	25150	25402	25076	106.2	96.1	99.2
↑Level 4 Phase											
Boundary Level 5/Level 4					25326	25164	25514	25114			98.7

COL4219.1.1	25350	25012	25626	24932	25564	25224	25630	25184	74.9	95.6	99.5
OxA-X-3058-10	25568	25086	25730	24792	25568	25266	25684	25188	107.9	95.9	99.6
↑Level 5 Phase											
Boundary Level 6/5					25900	25594	25930	25290			98.3
COL4221.1.1	25964	25790	26070	25668	25930	25790	25996	25712	109.6	96.2	99.5
COL4222.1.1	25964	25794	26070	25672	25932	25792	25996	25712	108.6	96.2	99.4
↑Level 6 Phase											
Start Level 6 Boundary					26034	25820	26330	25736			97.3

Table S5. Detailed results of Bayesian Model 2 (Final model) (Fig. 6 in the main text). Modeled calibrated ages and Boundaries with the 68.2% and 95.4% probability ranges. Calculated with OxCal 4.4 (Bronk Ramsey et al. 2009a), using a General t-type Outlier Model (Bronk Ramsey 2009b) and IntCal20 (Reimer et al. 2020).

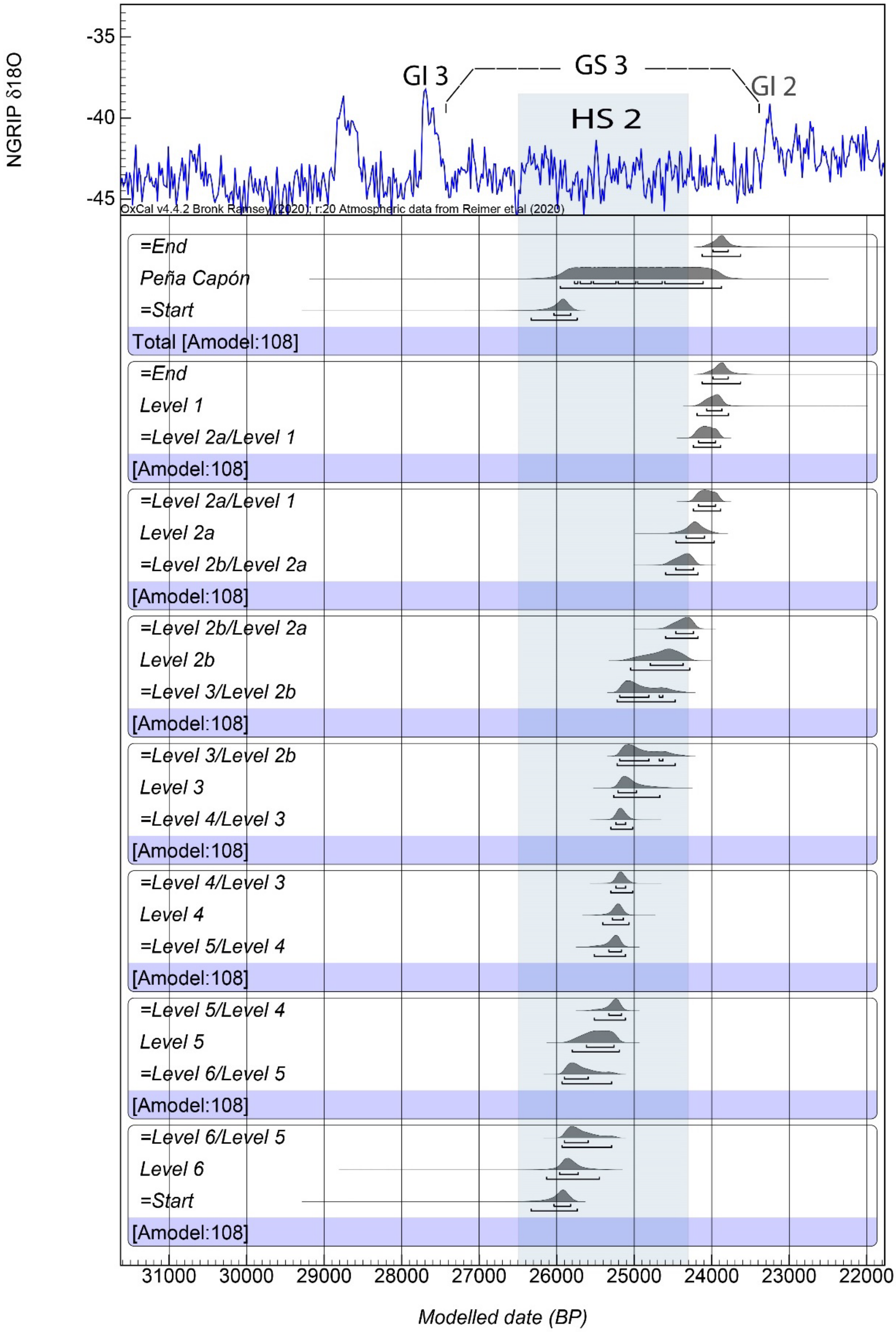

Figure S22. Probability Distribution Functions (PDFs) for the estimated duration of all archaeological levels at Peña Capón, based on the Bayesian Model 2 (FIg. 6 in the main text) as calculated by the 'date' command in Oxcal. Results are plotted against the δ18O record of the NGRIP ice core, indicating Greenland Interstadials 3 (GI 3) and 2 (GI2), Greenland Stadial 3 (Rasmussen et al. 2014), and the chronology of Heinrich Stadial 2 (blue bar) (Sánchez-Goñi and Harrison 2010).

Text S6. CQL Codes for Bayesian analyses

<u>Model 1 (Provisional Model)</u>:

```
Options()
 {
  Resolution=20;
 };
 Plot()
 {
  Outlier_Model("General",T(5),U(0,4),"t");
  Sequence("Peña Capón")
  {
   Boundary("Start");
   Phase("Level 6")
   {
    R_Date("COL4222.1.1", 21593, 121)
    {
     Outlier(0.05);
    };
    R_Date("COL4221.1.1", 21591, 124)
    {
     Outlier(0.05);
    };
   };
   Boundary("Level 6/Level 5");
   Phase("Level 5")
   {
    R_Date("OxA-39749", 21670, 130)
    {
     Outlier(0.05);
    };
    R_Date("OxA-X-3058-10", 20950, 180)
    {
     Outlier(0.05);
    };
    R_Date("COL4219.1.1", 20905, 118)
    {
     Outlier(0.05);
    };
   };
   Boundary("Level 5/Level 4");
   Phase("Level 4")
   {
    R_Date("COL4218.1.1", 21007, 118)
    {
     Outlier(0.05);
    };
    R_Date("OxA-39750", 20760, 110)
    {
```

```
  Outlier(0.05);
 };
 R_Date("OxA-39502", 20930, 110)
 {
  Outlier(0.05);
 };
};
Boundary("Level 4/Level 3");
Phase("Level 3")
{
 R_Date("OxA-39501", 20910, 110)
 {
  Outlier(0.05);
 };
 R_Date("COL4216.1.1", 20950, 128)
 {
  Outlier(0.05);
 };
 R_Date("COL4217.1.1", 20006, 107)
 {
  Outlier(0.05);
 };
};
Boundary("Level 3/Level 2b");
Phase("Level 2b")
{
 R_Date("OxA-39500", 20450, 110)
 {
  Outlier(0.05);
 };
 R_Date("OxA-39506", 20399, 63)
 {
  Outlier(0.05);
 };
 R_Date("OxA-X-3048-17", 20308, 150)
 {
  Outlier(0.05);
 };
};
Boundary("Level 2b/Level 2a");
Phase("Level 2a")
{
 R_Date("COL4212.1.1", 20278, 107)
 {
  Outlier(0.05);
 };
 R_Date("OxA-39499", 20370, 110)
 {
  Outlier(0.05);
 };
 R_Date("COL4215.1.1", 20261, 111)
```

```
   {
    Outlier(0.05);
   };
   R_Date("COL4213.1.1", 20107, 111)
   {
    Outlier(0.05);
   };
   R_Date("COL4214.1.1", 19987, 110)
   {
    Outlier(0.05);
   };
  };
  Boundary("Level 2a/Level 1");
  Phase("Level 1")
  {
   R_Date ("OxA-39505", 19950, 110)
   {
    Outlier(0.05);
   };
   R_Date ("OxA-39498", 19928, 27)
   {
    Outlier(0.05);
   };
   R_Date("COL4210.1.1", 20008, 112)
   {
    Outlier(0.05);
   };
  };
  Boundary("End");
 };
};
```

Model 2 (Final Model):

```
Options()
 {
  Resolution=20;
 };
 Plot()
 {
  Outlier_Model("General",T(5),U(0,4),"t");
  Sequence("Peña Capón")
  {
   Boundary("Start");
   Phase("Level 6")
   {
    R_Date("COL4222.1.1", 21593, 121)
    {
     Outlier(0.05);
    };
    R_Date("COL4221.1.1", 21591, 124)
    {
     Outlier(0.05);
    };
   };
   Boundary("Level 6/Level 5");
   Phase("Level 5")
   {
    R_Date("OxA-X-3058-10", 20950, 180)
    {
     Outlier(0.05);
    };
    R_Date("COL4219.1.1", 20905, 118)
    {
     Outlier(0.05);
    };
   };
   Boundary("Level 5/Level 4");
   Phase("Level 4")
   {
    R_Date("COL4218.1.1", 21007, 118)
    {
     Outlier(0.05);
    };
    R_Date("OxA-39750", 20760, 110)
    {
     Outlier(0.05);
    };
    R_Date("OxA-39502", 20930, 110)
    {
     Outlier(0.05);
    };
   };
   Boundary("Level 4/Level 3");
```

```
Phase("Level 3")
{
 R_Date("OxA-39501", 20910, 110)
 {
  Outlier(0.05);
 };
 R_Date("COL4216.1.1", 20950, 128)
 {
  Outlier(0.05);
 };
};
Boundary("Level 3/Level 2b");
Phase("Level 2b")
{
 R_Date("OxA-39500", 20450, 110)
 {
  Outlier(0.05);
 };
 R_Date("OxA-39506", 20399, 63)
 {
  Outlier(0.05);
 };
 R_Date("OxA-X-3048-17", 20308, 150)
 {
  Outlier(0.05);
 };
};
Boundary("Level 2b/Level 2a");
Phase("Level 2a")
{
 R_Date("COL4212.1.1", 20278, 107)
 {
  Outlier(0.05);
 };
 R_Date("OxA-39499", 20370, 110)
 {
  Outlier(0.05);
 };
 R_Date("COL4215.1.1", 20261, 111)
 {
  Outlier(0.05);
 };
 R_Date("COL4213.1.1", 20107, 111)
 {
  Outlier(0.05);
 };
 R_Date("COL4214.1.1", 19987, 110)
 {
  Outlier(0.05);
 };
};
```

```
 Boundary("Level 2a/Level 1");
 Phase("Level 1")
 {
  R_Date ("OxA-39505", 19950, 110)
  {
   Outlier(0.05);
  };
  R_Date ("OxA-39498", 19928, 27)
  {
   Outlier(0.05);
  };
  R_Date("COL4210.1.1", 20008, 112)
  {
   Outlier(0.05);
  };
 };
 Boundary("End");
};
Sequence()
{
 Boundary("=Start");
 Date("Level 6");
 Boundary("=Level 6/Level 5");
};
Sequence()
{
 Boundary("=Level 6/Level 5");
 Date("Level 5");
 Boundary("=Level 5/Level 4");
};
Sequence()
{
 Boundary("=Level 5/Level 4");
 Date("Level 4");
 Boundary("=Level 4/Level 3");
};
Sequence()
{
 Boundary("=Level 4/Level 3");
 Date("Level 3");
 Boundary("=Level 3/Level 2b");
};
Sequence()
{
 Boundary("=Level 3/Level 2b");
 Date("Level 2b");
 Boundary("=Level 2b/Level 2a");
};
Sequence()
{
 Boundary("=Level 2b/Level 2a");
```

```
 Date("Level 2a");
 Boundary("=Level 2a/Level 1");
};
Sequence()
{
 Boundary("=Level 2a/Level 1");
 Date("Level 1");
 Boundary("=End");
};
Sequence("Total")
{
 Boundary("=Start");
 Date("Peña Capón");
 Boundary("=End");
};
};
```

SI.6 – Pollen

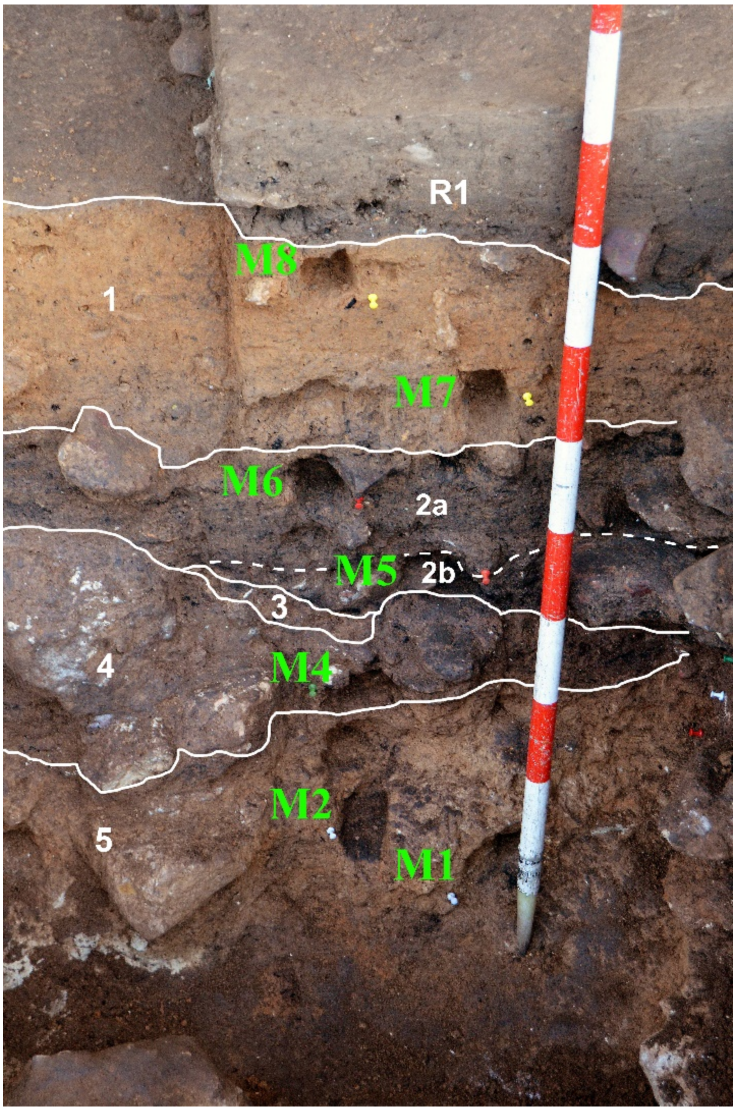

Figure S23. Stratigraphic sequence recorded in the southern profile of square 2B showing sample location for pollen analysis.

SI.7 – Microvertebrates

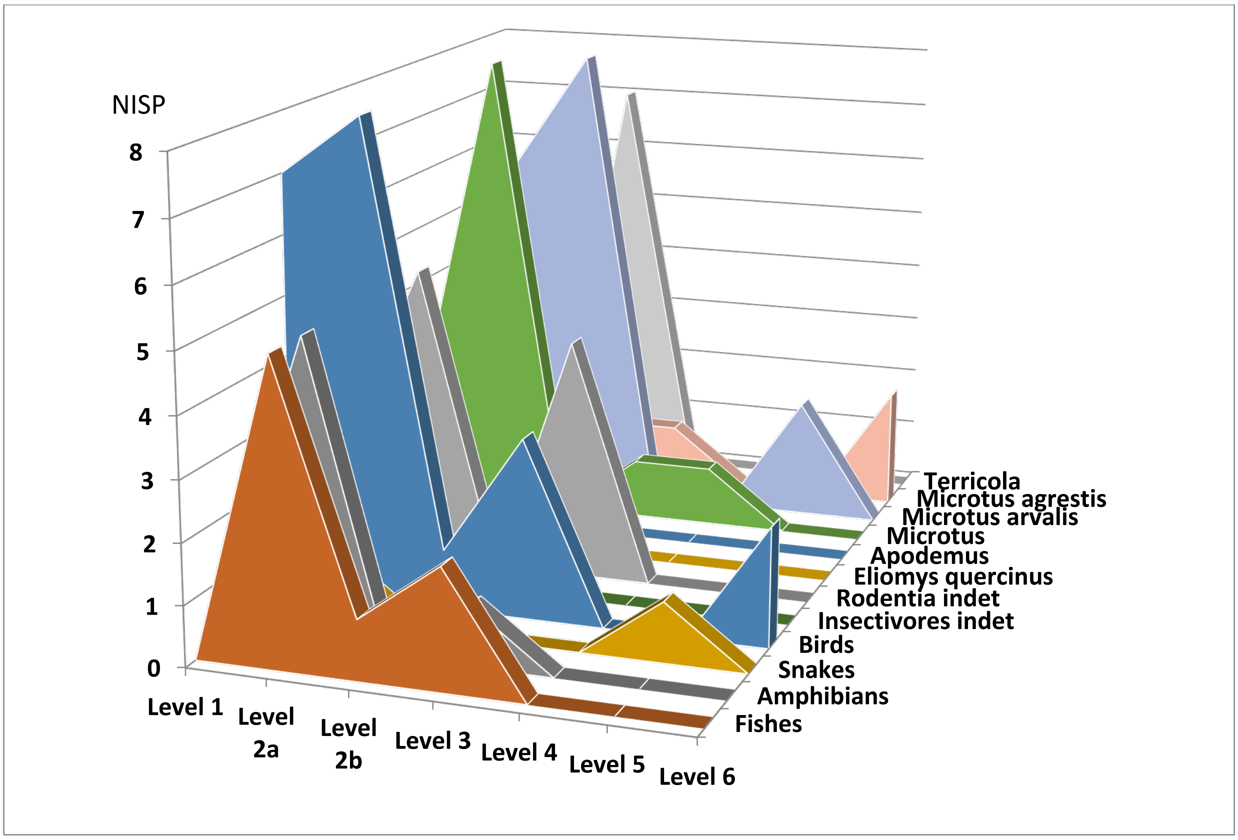

Figure S24. Distribution of small vertebrates (NISP) throughout the Peña Capón sequence (Levels 1-6). Note the high representation of Terricola in level 2b and the species of Microtus in levels 5 and 6.

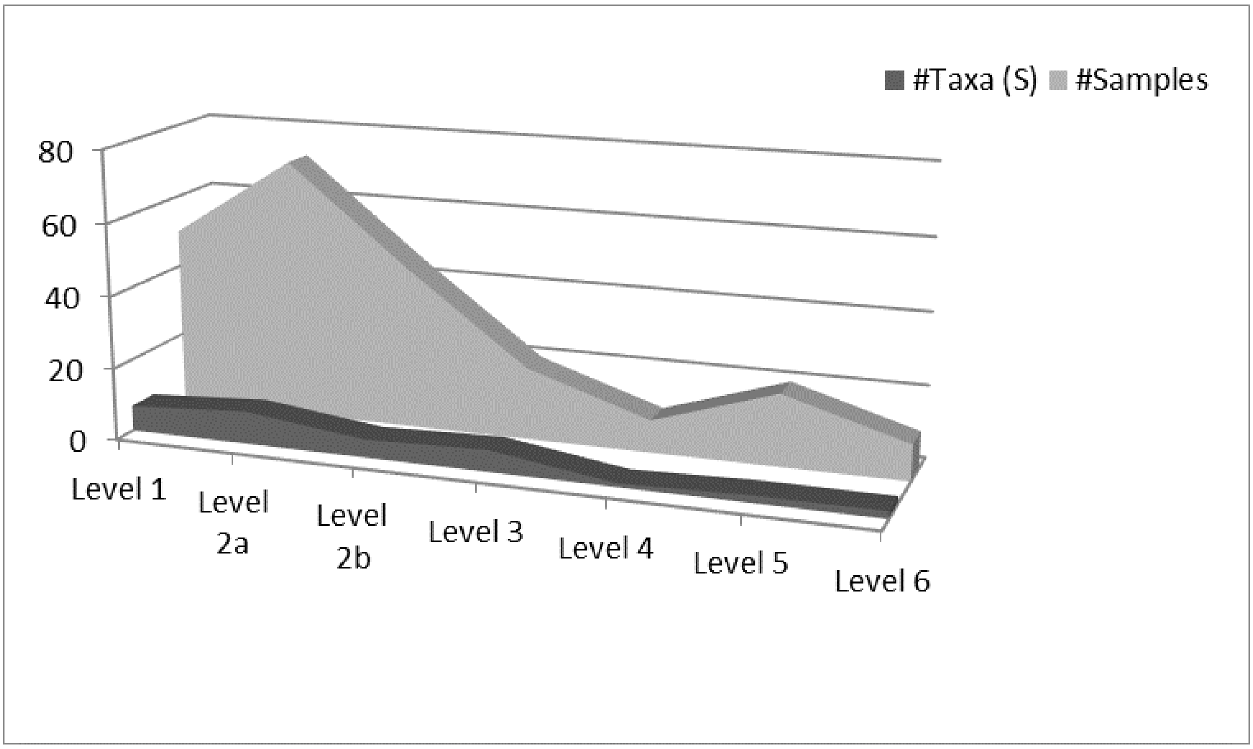

Figure S25. Relation between collected sediment samples and identified taxa of small vertebrates at Peña Capón.

SI.8 – Macrovertebrates

Text S7. Mortality patterns and taphonomic analysis

All taxa recorded in Peña Capón are dominated by adult individuals throughout the sequence, although juvenile and infant individuals are also found in some cases (Table S6). In level 1, the only species having yielded non-adult individuals is the rabbit, which has shown 1 juvenile and 1 infant. Level 2a shows juvenile and infant individuals concerning horses, chamois and rabbits, while level 2b shows one infant ibex, and level 6 one juvenile horse and one infant horse (Table 3). As for the relative abundance of infant and juvenile rabbits in the Solutrean levels (1, 2a and 2b), it should be noted that while they could represent natural deaths occurred within their burrows, no individuals have been found in anatomical connection, and their only articulated elements are those of lower appendicular limb bones (metapodials, carpals and tarsals).

**Level**	**1**	**2a**	**2b**	**3**	**4**	**5**	**6**
**MNI**	**S/A/J/I**	**S/A/J/I**	**S/A/J/I**	**S/A/J/I**	**S/A/J/I**	**S/A/J/I**	**S/A/J/I**
Bos/Bison		0/1/0/0	0/1/0/0				
Equus caballus	0/1/0/0	0/1/1/1	1/2/0/0	1/1/0/0	0/1/0/0	0/1/0/0	1/1/1/1
Cervus elaphus	0/1/0/0	0/2/0/0	0/1/0/0	0/1/0/0	0/1/0/0		
Capreolus capreolus	0/1/0/0						
Capra pyrenaica	0/1/0/0	0/1/0/0	0/1/0/1	0/1/0/0		0/1/0/0	
Rupicapra pyrenaica	0/1/0/0	1/1/1/1	0/1/0/0				
Meles meles		0/1/0/0					
Felix silvestris		0/1/0/0					
Oryctolagus cuniculus	0/6/1/1	0/5/2/1	0/2/1/0	0/2/0/0	0/1/0/0	0/1/0/0	0/1/0/0

Table S6. Mortality patterns among macrovertebrates at the Peña Capón sequence (S: Senile, A: Adult, J: Juvenile, I: Infant).

From a taphonomic perspective, the studied sample shows a high degree of breakage, given that 80% of bone remains are smaller than 3 cm and 97% smaller than 5 cm. This intense fragmentation is probably the main cause of the relatively scarce taxonomic representation identified at the site (Table 4 in the main text). Furthermore, 25% of bone remains show bad preservation of cortical surfaces, which can be related to both their small dimensions and different types of bone surface modifications. Among the physical modifications identified, the best-represented are those of water origin, as showed by minor traces of polishing and abrasion, which nonetheless affect just 11.5% of the NISP, with only slightly higher incidences in levels 3 and 2a (Table S7). This shows that impact of hydraulic jumbles, or water transportation in general, has been low throughout the sequence, and thus archaeological assemblages were not substantially altered by such processes. The presence of manganese oxide stains (especially in levels 3 and 4) and carbonate crusts (mostly in levels 1 to 3) on some bone surfaces (Table 4) is due to the secondary precipitation of mineral components during the diagenesis, as noted in the micro-scale sedimentary analysis of the deposit.

Surface modifications/levels	1	2a	2b	3	4	5	6	Total
NISP	3562	6161	4496	1368	151	1465	55	17269
NISP with good preservation excluding teeth	1997	3779	2808	361	81	636	41	9704
%NISP <3cm	84.1	74.9	3509	86.8	85.9	94.9	83.6	80.4
%NISP 3,1-5cm	12.6	21.8	893	9.6	6.2	4.2	14.5	16.8
%NISP >5,1cm	3.3	3.3	95	3.6	7.9	0.9	1.8	2.8
% NISP Bad surface preservation	25.1	22	24.1	42.8	42.4	22.1	9.1	25
% NISP Weathering	5.6	3.5	2.2	3.7	1.7	5.3	0	3.7
% NISP Manganese	9.1	6.4	5.4	21.8	54.2	0.5	0	6.1
%NISP Carbonate crust	15.2	9.1	7.4	2.5	0	0.3	0	2.5
%NISP Biochemical	12.6	11.4	7.6	11.7	6.2	1.3	7.3	10.2
%NISP Hydrological alteration	6.7	15.5	10.7	18.7	2.8	4.4	1.8	11.5
%NISP Trampling	2.9	2.4	1.7	8.6	1.2	1.3	0	2.7
%NISP Tooth mark	0.2	0.3	0.2	1.7	0	0	0	0.4
%NISP Cut Mark	2.8	1.3	1	9.1	2.5	0.5	9.8	1.8
%NISP Percussion Mark	0.2	1	0.2	1.7	9.9	0.5	0	0.6
%NISP Thermal alteration	35.8	27.5	34.7	15.4	33.3	17.8	18.2	29.5

Table S7. Bone surface modifications recorded at the Peña Capón macrovertebrates assemblages.

Subaerial weathering shows low incidences in all levels, with only 3.7% of the remains classified as Behrensmeyer's, (1978) stages 3-4 (table 4), thus suggesting that sedimentation rates were high throughout the sequence. Biochemical modifications are more frequent (10.2%), but still not relevant in general terms (Table 4). Trampling marks are also low (2.7%), with only a slightly higher incidence recorded in level 3 (8.6%). Lastly, carnivore intervention as showed by tooth marks is documented in only 0.4% of the sample, with only level 3 showing a, still low, 1.7% of bones yielded these marks (Table S7). Therefore, carnivores were not a main accumulating agent in any of the archaeological levels recorded at Peña Capón, as it was previously attested for the assemblages coming from the 1970 excavation at the site (Yravedra *et al*. 2016).

Concerning human intervention, cut marks (Fig. S27) and percussion marks, as well as thermal alterations, are found in all layers (Table 4), thus suggesting that all cooking processes related to faunal individuals, including marrow extraction, were conducted at the site. Thermal alterations are especially high in layers 1, 2a, 2b and 4, which is in consonance with the existence of small fire structures at least in levels 2a and 2b.

Anthropogenic modifications have been recorded throughout the sequence in most of the identified taxa, excepting chamois, roe deer and carnivores. All other species, including leporids in levels 1, 2a and 3, show high rates of cut marks (Table S8), thus indicating their exploitation by humans. Among processes related to meat exploitation, we have identified defleshing activities on remains of large bovids, horses, red deers, rabbits and undetermined large, medium and small-sized animals. Disarticulation marks have been observed on rabbits and undetermined small-sized animals, while evisceration evidence have been recorded on red

deers, ibex and undetermined medium and small-sized animals, as showed by the presence of cut-marks on their ribs ventral sides.

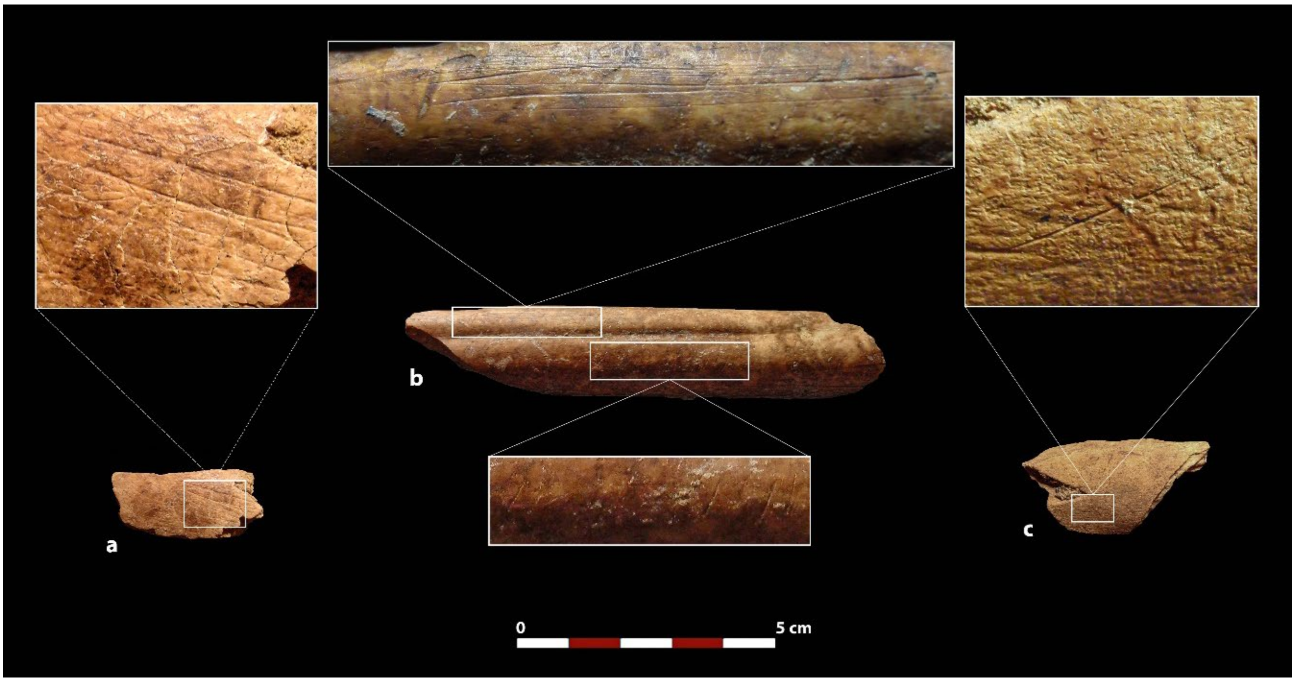

Figure S27. Examples of cut-marked shafts from level 1 (a), level 2a (b) and level 3 (c). All of them were sampled for radiocarbon dating (OxA-39498, OxA-39499, OxA-39501 respectively; see Table S3).

As for breakage patterns, we have recorded percussion marks on bone remains from ibex, red deer, horse and undetermined large, medium and small-sized animals (Table S9), thus suggesting that these bones were broken while fresh, and most likely with the purpose of seeking access to marrow. These actions, together with fire activity as observed in thermal alterations, were probably relevant causes contributing to the high degree of breakage observed in all assemblages throughout the sequence.

%NISP CM	1	2a	2b	3	4	5	6
Bos/Bison		100.0					
Equus caballus	60.0	20.0	25.0			50.0	
Cervus elaphus		25.0		12.5	50.0		
Capra pyrenaica		14.3		33.3			
Oryctolagus cuniculus	3.7	6.5		17.1			
Indet. large size	6.7	18.2	10.0	50.0			
Indet. medium size	12.3	6.4	13.6	3.8	14.3	28.6	100.0
Indet. small size	6.4	4.1	1.7	5.7			100.0
Indetermined	0.3	0.1	0.3	3.2			5.1
Birds indet.		40.0					

Table S8. Frequencies of cut marks recorded at the Peña Capón macrovertebrates assemblages (CM = Cut marks). Remains with bad preserved surfaces and teeth are not included.

%NISP PM	1	2a	2b	3	4
Equus caballus	20.0	20.0			
Cervus elaphus		25.0	20.0	25.0	100.0
Capra pyrenaica			50.0		
Indet. large size		12.1	10.0		
Indet. medium size		4.6		11.5	14.3
Indet. small size	1.0	3.4		0.5	
Indetermined		0.1		0.6	

Table S9. Frequencies of percussion marks recorded at the Peña Capón macrovertebrates assemblages (PM = Percussion marks).

Methods for mortality patterns and taphonomic analysis

Mortality patterns were divided into (1) infant, (2) juvenile-prime adult, (3) adult and (4) senile as explained in Alcaraz-Castaño et al. (2017). Age profiles were estimated from tooth crown wear and the emergence of the teeth according to Stelle (2002) for deer, Pérez Ripoll (1988) for ibex and Levine (1982) for Equus.

A systematic observation of bone surfaces to explore the presence of cut, percussion and tooth marks was also carried out with 10X-20X hand lenses and different lighting (Blumenschine, 1995). Our diagnostic criteria for cut, tooth and percussion marks are the ones defined respectively by Bunn (1982) and Potts and Shipman (1981), Blumenschine (1988, 1995), and Blumenschine and Selvaggio (1988). For comparative purposes, observation of bone surfaces includes the observation of epiphysis and shafts (Blumenschine, 1988, 1995). Modifications of bone surfaces were also quantified by types of fragments and bone sections (Domínguez-Rodrigo & Barba 2005) based on NISP values. The presence of tooth, percussion and cut marks was recorded for the whole assemblages, and percentages of tooth, percussion and cut marks included only bones with a good surface preservation. Weathering stages were also observed following Behrensmeyer (1978) to estimate the bone subaerial time exposure. Water effects on bone surfaces were estimated according to the presence of abrasion, polishing, rounded bones, and carbonates following Parsons and Brett (1991), Cáceres [2002] and Yravedra (2006). Signs of polishing, rounding or abrasion are to be expected in transported assemblages, but also in non-transported assemblages exposed to circulating water and mobile sediments, such as those encased in sand strata (Thompson et al 2011). Biochemical alterations were estimated according to Domínguez-Rodrigo & Barba (2006). To differentiate between green and dry fractures on long bones we analyzed shafts larger than 30 mm following Villa & Mahieu's (1991) criteria.

Acknowledgements

We are in debt to Antonio Alcaína for providing us with the archaeological assemblages and materials from the 1972 excavation at Peña Capón. We also acknowledge contributions by students from the Universities of Alcalá, Complutense de Madrid, Autónoma de Madrid, Valencia and Zaragoza for their valuable work in the field and the lab, as well as to the members of the speleological group GAEM (*Grupo de Actividades Espeleológicas de Madrid*).

We thank Marianne Dohms and Dr. Philipp Schulte (Physical Geography, RWTH Aachen University) for spectrophotometric and granulometric analyses. Elena Hensel and Darius Cichy (University of Cologne) conducted measurements for total carbon, carbonate and magnetic susceptibility.

Photos were taken and processed by J.J. Alcolea-González, Luis Luque, Manuel Alcaraz-Castaño, Rodrigo de Balbín-Behrmann, Alfonso Dávila and Zulema Picazo. The drone video was recorded by Alberto Alcaraz-Castaño and edited by Guillermo Jiménez-Gisbert.

Supplementary references

1. Alcaraz-Castaño, M. et al. Los orígenes del Solutrense y la ocupación pleniglaciar del interior de la Península Ibérica: implicaciones del nivel 3 de Peña Capón (valle del Sorbe, Guadalajara). *Trab. de Prehist.* **70(1),** 28-53 (2013). http://tp.revistas.csic.es/index.php/tp/article/view/637/659
2. Alcaraz-Castaño, M., Alcolea-González, J.J., Balbín Behrmann, R. de, Kehl, M., Weniger, G.C. Recurrent Human Occupations in Central Iberia around the Last Glacial Maximum. The Solutrean Sequence of Peña Capón Updated. In *Human Adaptations to the Last Glacial Maximum: The Solutrean and Its Neighbors* (eds. I. Schmidt & J. Cascalheira), 148–170 (Cambridge Scholars Publishing, Cambridge, UK, 2019).
3. Alcaína, A., **Alcaraz-Castaño, M.** & Alcolea-González, J. J. Espeleología al servicio de la arqueología. Descubrimiento e investigación de los yacimientos de Peña Cabra y Peña Capón (Muriel, Guadalajara). *ESPELEOMADRID* **8(Época III),** 94-98 (2018).
4. Alcolea-González J. J. et al. Avance al estudio del poblamiento paleolítico del Alto Valle del Sorbe (Muriel, Guadalajara). In *II Congreso de Arqueología Peninsular I, Paleolítico y Epipaleolítico* (eds. Balbín R. de & Bueno, P.), 201-218 (Fundacion Rei Afonso Henriques, Zamora, 1997).
5. Alcaraz-Castaño, M. Central Iberia around the Last Glacial Maximum. Hopes and Prospects. *J. Anthropol. Res.* **71 (4),** 565-578 (2015). https://doi.org/10.3998/jar.0521004.0071.406
6. Delibes, G. & Díez F. ¿Una Meseta desolada? Estado actual de la investigación sobre el Paleolítico Superior en las regiones interiores de la Península Ibérica. In *El Paleolítico Superior en la Meseta Norte española* (eds. Delibes, G. & Díez F.), 11-40 (Valladolid, Universidad de Valladolid, Fundación Duques de Soria, 2006).
7. Mosquera, M. Valle de las Orquídeas: un yacimiento al aire libre del Pleistoceno Superior en la Sierra de Atapuerca (Burgos). *Trab. de Prehist.* **64(2),** 143-155 (2007). https://doi.org/10.3989/tp.2007.v64.i2.113
8. Cacho, C. et al. El Paleolítico superior en el interior de la Península Ibérica. Revisión crítica y perspectivas de futuro. In *El Paleolítico Superior Peninsular. Novedades del Siglo XXI* (ed. Mangado, X.), 115-136 (SERP, Universitat de Barcelona, Barcelona, 2010).
9. Clark, P. U. et al. The Last Glacial Maximum. *Science* **325(5941),** 710-714 (2009). https://doi.org/10.1126/science.1172873
10. Yravedra, J. et al. Not so deserted…paleoecology and human subsistence in Central Iberia (Guadalajara, Spain) around the Last Glacial Maximum. *Quat. Sci. Rev.* **140,** 21-38. https://doi.org/10.1016/j.quascirev.2016.03.021
11. Zilhão, J. & Aubry, T. La Pointe de Vale Comprido et les origines du Solutréen. *L'Anthropologie* **99,** 125–142 (1995).
12. Zilhão J. *O Paleolítico Superior da Estremadura portuguesa*. (Edições Colibri, Lisboa, II vols., 1997).
13. Renard, C. Continuity or discontinuity in the Late Glacial Maximum of south-western Europe: the formation of the Solutrean in France. *World. Archaeol.* **43(4),** 726-743 (2011).

14. Belmiro, J, Bicho, N., Haws, J. & Cascalheira, J. The Gravettian-Solutrean transition in westernmost Iberia: New data from the sites of Vale Boi and Lapa do Picareiro. *Quat. Int.* **in press,** in press (2020). https://doi.org/10.1016/j.quaint.2020.08.027
15. Bronk Ramsey, C. Bayesian analysis of radiocarbon dates. *Radiocarbon* **51(1)**, 337-360 (2009). https://doi.org/10.1017/S0033822200033865
16. Reimer, P. et al. The IntCal20 Northern Hemisphere radiocarbon age calibration curve (0–55 cal kBP). *Radiocarbon* **62 (4),** 725-757 (2020). https://doi.org/doi:10.1017/RDC.2020.41
17. Almeida, F. *The terminal Gravettian of Portuguese Estremadura: Technological Variability of the Lithic Industries*. PhD Thesis, (Southern Methodist University, Dallas, Texas, 2000), https://doi.org/10.13140/RG.2.2.23479.32169
18. Blott, S.J. & Pye, K. GRADISTAT: a grain size distribution and statistics package for the analysis of unconsolidated sediments. *Earth. Surf. Process. Landf.* **26,** 1237-1248 (2001). https://doi.org/10.1002/esp.261
19. Eckmeier, E., et al. Preservation of fire-derived carbon compounds and sorptive stabilisation promote the accumulation of organic matter in black soils of the Southern Alps. *Geoderma* **159,** 147-155 (2013).
20. Dearing, J.A. *Environmental magnetic susceptibility. Using the Bartington MS2 system*. (Chi Publishing, Kenilworth, 1999). https://doi.org/10.1016/j.geoderma.2010.07.006
21. Stoops, G., 2003. *Guidelines for the analysis and description of soil and regolith thin sections* (Soil Science Society of America, Madison, WI, 2003).
22. Angelucci, D. E. The recognition and description of knapped lithic artifacts in thin section. *Geoarchaeology* **25(2),** 220–232 (2010). https://doi.org/10.1002/gea.20303
23. Rentzel, P. et al. Trampling, Poaching and the Effect of Traffic. In *Archaeological soil and sediment micromorphology* (eds. Nicosia, C. & Stoops, G.), 281-297 (John Wiley & Sons Ltd., 2017)
24. Kooistra, M. J. & Pulleman, M. M. Chapter 16 - Features Related to Faunal Activity. *In Interpretation of Micromorphological Features of Soils and Regoliths* (eds. Stoops, G., Marcelino, V. & Mees, F.), 447-469 (Elsevier, 2018, 2nd edition).
25. Durand, N., Monger, H. C., Canti, M. G. & Verrecchia, E. P. Calcium Carbonate Features. *In Interpretation of Micromorphological Features of Soils and Regoliths* (eds. Stoops, G., Marcelino, V. & Mees, F.), 205-258 (Elsevier, 2018, 2nd edition).
26. Kehl, M. et al. Late Neanderthals at Jarama VI (central Iberia)? *Quat. Res.* **80,** 218-234 (2013). https://doi.org/10.1016/j.yqres.2013.06.010
27. Kehl, M. et al. The rock shelter Abrigo del Molino (Segovia, Spain) and the timing of the late Middle Palaeolithic in Central Iberia. *Quat. Res.* **90,** 180-200 (2018). https://doi.org/10.1017/qua.2018.13
28. Mallol, C. & Goldberg, P. Caves and Rockshelter Sediments. In *Archaeological Soil and Sediment Micromorphology* (eds. Nicosia, C. & Stoops, G.), 359-381 (Wiley, Hoboken, NJ, 2017).
29. van Vliet-Lanoë, B., Cox, C.A., 2018. Frost Action. In Interpretation of Micromorphological Features of Soils and Regoliths (eds. Stoops, G., Marcelino, V. & Mees, F.), 575-603 (Elsevier, 2018, 2nd edition).
30. Bronk Ramsey, C. Dealing with outliers and offsets in radiocarbon dating. *Radiocarbon* **51(3)**, 1023-1045 (2009). https://doi.org/10.1017/S0033822200034093
31. Rasmussen, S. O. et al. A stratigraphic framework for abrupt climatic changes during the Last Glacial period based on three synchronized Greenland ice-core records: refining and extending the INTIMATE event stratigraphy, *Quat. Sci. Rev.*, **106**, 14-28 (2014). https://doi.org/10.1016/j.quascirev.2014.09.007
32. Sanchez Goñi, M. F. & Harrison, S. P. Millennial-scale climate variability and vegetation changes during the Last Glacial: Concepts and terminology. *Quat. Sci. Rev.*, **29(21-22)**, 2823-2827 (2010). https://doi.org/10.1016/j.quascirev.2009.11.014

33. Behrensmeyer, A. K. Taphonomic and ecological information from bone weathering. *Paleobiology* **4 (2),** 150-162 (1978).
34. Alcaraz-Castaño, M. et al. A context for the last Neandertals of interior Iberia: Los Casares cave revisited. PLoS ONE 12(7), e0180823 (2017). https://doi.org/10.1371/journal.pone.0180823
35. Steele, T. E. *Red deer: their ecology and how they were hunted by Late Pleistocene hominids in western Europe*. Ph.D. dissertation (Stanford University, California, 2002).
36. Pérez-Ripoll, M. Estudio de la secuencia del desgaste de los molares de *Capra pyrenaica* de los yacimientos prehistóricos. *Archivo de Prehistoria Levantina* **18,** 83-128 (1988)
37. Levine, M. A. The use of crown height measurements and eruption-wear sequence to age horse teeth. In *Aging and sexing from archaeological sites* (eds. Wilson, B., Grigson, C. & Payne S), 223-250 (Oxfordm, BAR, Archaeopress, 1982).
38. Blumenschine R. Percussion marks, tooth marks and the experimental determinations of the timing of hominid and carnivore access to long bones at FLK Zinjanthropus, Olduvai Gorge, Tanzania. *J. Hum. Evol.* **29,** 21-51 (1995). https://doi.org/10.1006/jhev.1995.1046
39. Bunn HT. *Meat-eating and human evolution: studies on the diet and subsistence patterns of Plio-Pleistocene hominins in East Africa*. Ph.D. Dissertation (University of California, Berkeley, 1982).
40. Potts, R., Shipman, P. Cutmarks made by stone tools on bones from Olduvai Gorge, Tanzania. *Nature* **291,** 577-580 (1981). https://doi.org/10.1038/291577a0
41. Blumenschine, R., Selvaggio, M. Percussion marks on bone surfaces as a new diagnostic of hominid behaviour. *Nature* **333,** 763–765 (1988). https://doi.org/10.1038/333763a0
42. Domínguez-Rodrigo, M. & Barba, R. A study of cut marks on small-sized carcasses and its application to the study of cut marked bones from small mammals at the FLK Zinj site. *Journal of Taphonomy* **3,** 121-134 (2005).
43. Parsons, K. M. & Brett, E. Taphonomic processes and biases in modern marine environments: an actualistic perspective on fossil assemblage preservation. In *The processes of fossilization* (ed. Donovan, S.K.) (Columbia University Press, New York, 1991)
44. Cáceres, I. *Tafonomía de yacimientos antrópicos en karst. Complejo Galería (Sierra de Atapuerca, Burgos), Vanguard Cave (Gibraltar) y Abric Romaní (Capalledes, Barcelona)*. Ph.D. dissertation, (Universitat Rovira i Virgili, Tarragona, 2002).
45. Yravedra, J. *Tafonomía aplicada a Zooarqueología* (Aula Abierta-UNED, Madrid 2006).
46. Thompson, C. E., Ball, S., Thompson, T. J. U. & Gowland, R. The abrasion of modern and archaeological bones by mobile sediments: the importance of transport modes. *J. Arch. Sci.* **38(4),** 784-793 (2011). https://doi.org/10.1016/j.jas.2010.11.001
47. Domínguez-Rodrigo, M. & Barba, R. New estimates of tooth mark and percussion mark frequencies at the FLK ZINJ site: The carnivore-hominid-carnivore hypothesis falsified. *J. Hum. Evol.* **50(2),** 170-194 (2006). https://doi.org/10.1016/j.jhevol.2005.09.005
48. Villa, P. & Mahieu, E. Breakage patterns of human long bones. *J. Hum. Evol.* **21(1),** 27–48 (1991). https://doi.org/10.1016/0047-2484(91)90034-S
